# Glucose-nucleobase pairs within DNA: impact of hydrophobicity, alternative linking unit and DNA polymerase nucleotide insertion studies†
†Electronic supplementary information (ESI) available. See DOI: 10.1039/c7sc04850e


**DOI:** 10.1039/c7sc04850e

**Published:** 2018-03-05

**Authors:** Empar Vengut-Climent, Pablo Peñalver, Ricardo Lucas, Irene Gómez-Pinto, Anna Aviñó, Alicia M. Muro-Pastor, Elsa Galbis, M. Violante de Paz, Célia Fonseca Guerra, F. Matthias Bickelhaupt, Ramón Eritja, Carlos González, Juan Carlos Morales

**Affiliations:** a Department of Biochemistry and Molecular Pharmacology , Instituto de Parasitología y Biomedicina López Neyra , CSIC , PTS Granada , Avda. del Conocimiento, 17, 18016 Armilla , Granada , Spain . Email: jcmorales@ipb.csic.es; b Departamento de Química Orgánica y Farmacéutica , Facultad de Farmacia , Universidad de Sevilla , C/Prof. García González 2 , 41012-Sevilla , Spain; c Instituto de Química Física ‘Rocasolano’ , CSIC , C/. Serrano 119 , 28006 Madrid , Spain; d Instituto de Química Avanzada de Cataluña (IQAC) , CSIC , CIBER – BBN Networking Centre on Bioengineering, Biomaterials and Nanomedicine , Jordi Girona 18-26 , E-08034 Barcelona , Spain; e Instituto de Bioquímica Vegetal y Fotosíntesis , CSIC – Universidad de Sevilla , Américo Vespucio 49 , 41092 , Sevilla , Spain; f Department of Theoretical Chemistry , Amsterdam Center for Multiscale Modeling , Vrije Universiteit Amsterdam , De Boelelaan 1083 , 1081 HV Amsterdam , The Netherlands; g Leiden Institute of Chemistry , Leiden University , PO Box 9502 , 2300 RA Leiden , The Netherlands; h Institute of Molecules and Materials (IMM) , Radboud University , Heyendaalseweg 135 , 6525 AJ Nijmegen , The Netherlands

## Abstract

Recently, we studied glucose-nucleobase pairs, a binding motif found in aminoglycoside–RNA recognition. DNA duplexes with glucose as a nucleobase were able to hybridize and were selective for purines. They were less stable than natural DNA but still fit well on regular B-DNA. These results opened up the possible use of glucose as a non-aromatic DNA base mimic. Here, we have studied the incorporation and thermal stability of glucose with different types of anchoring units and alternative apolar sugar-nucleobase pairs. When we explored butanetriol instead of glycerol as a wider anchoring unit, we did not gain duplex thermal stability. This result confirmed the necessity of a more conformationally restricted linker to increase the overall duplex stability. Permethylated glucose-nucleobase pairs showed similar stability to glucoside-nucleobase pairs but no selectivity for a specific nucleobase, possibly due to the absence of hydrogen bonds between them. The three-dimensional structure of the duplex solved by NMR located both, the hydrophobic permethylated glucose and the nucleobase, inside the DNA helix as in the case of glucose-nucleobase pairs. Quantum chemical calculations on glucose-nucleobase pairs indicate that the attachment of the sugar to the DNA skeleton through the OH1 or OH4 positions yields the highest binding energies. Moreover, glucose was very selective for guanine when attached through OH1 or OH4 to the DNA. Finally, we examined DNA polymerase insertion of nucleotides in front of the saccharide unit. KF^–^ polymerase from *E. coli* inserted A and G opposite glc and 6dglc with low efficiency but notable selectivity. It is even capable of extending the new pair although its efficiency depended on the DNA sequence. In contrast, Bst 2.0, SIII and BIOTAQ™ DNA polymerases seem to display a loop-out mechanism possibly due to the flexible glycerol linker used instead of deoxyribose.

## Abstract 2

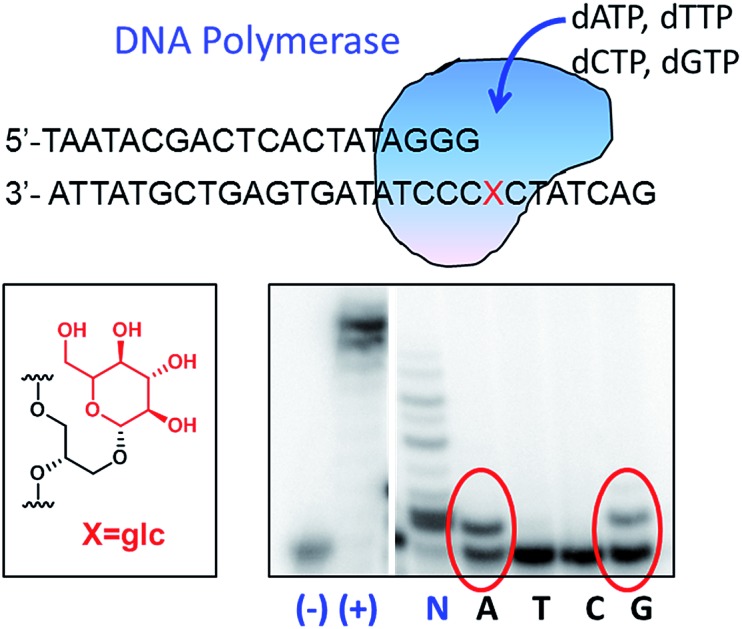
Glucose-nucleobase pairs were designed, synthesized and incorporated into duplex DNA. Their stability, structure and polymerase replication was investigated.

## Introduction

**Fig. 1:**
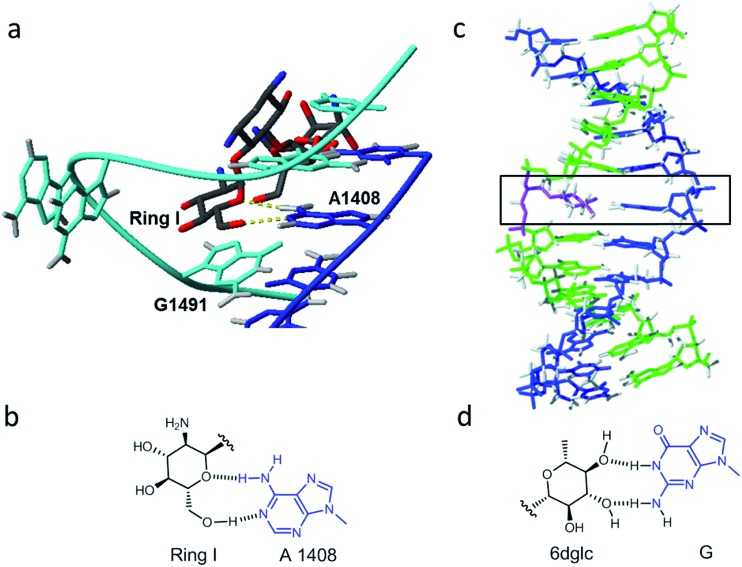
(a) Detail of the solution structure of aminoglycoside paromomycin binding a 16S RNA model sequence (pdb number 1J7T); (b) drawing of the glycoside-adenine 4108 pair showing the two hydrogen bonds formed; (c) refined solution structure of a double helix containing a 6dglc-G pair (indicated by the black rectangle) (PDB number ; 2N9F); and (d) drawing of the 6-deoxyglucose-guanine pair showing the two hydrogen bonds formed.

Molecular interactions play a key role in the communication between biomolecules, including drugs binding to their targets and the organization of supramolecular assemblies, nanostructures and biopolymers.^1^–^4^ Organic chemists keep studying these binding motifs and proposing new lego pieces for future designs.^5^,^6^ Our research group has been interested in the least studied interactions that appear during the recognition of aminoglycosides for ribosomal RNA.^7^–^9^ While electrostatic forces and hydrogen bonds between the hydroxyl and amino groups of aminoglycosides and the phosphate groups of RNA are quite apparent, other binding motifs can also be observed on the numerous X-ray and NMR structures.^8^,^10^ For example, the aminoglucose ring I of paromomycin stacks on top of guanine 1491 of 16S ribosomal RNA (Fig. 1a). By using carbohydrate-oligonucleotide conjugates as model systems, we have demonstrated this type of contact to be energetically favorable.^11^,^12^ In fact, we have also observed monosaccharide stacking on top of the guanine tetrad of a G-quadruplex DNA structure.^13^

**Fig. 2:**
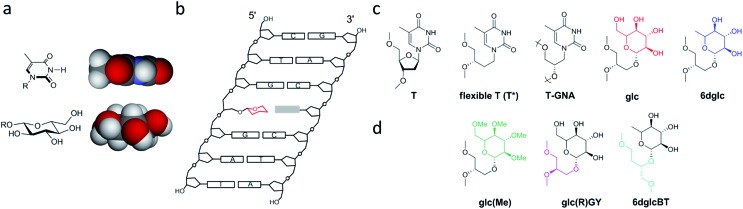
Description of the carbohydrate derivatives under study. (a) CPK models of thymine and glucose. (b) Schematic drawing of a DNA double helix containing a glycoside-nucleobase pair. (c) Structures of the modifications incorporated into the DNA duplex (from ref. 15): thymidine (T), (*S*)-3,4-dihydroxybutyl thymine or flexible T (T*), glycol T (T-GNA), (*S*)-2,3-dihydroxypropyl glucose (glc) and (*S*)-2,3-dihydroxypropyl 6-deoxyglucose (6dglc). (d) Structures of the modifications incorporated into the DNA duplex (this work): (*S*)-2,3-dihydroxypropyl permethylated glucose (glc(Me)), (*R*)-2,3-dihydroxypropyl glucose (glc(*R*)GY), and (*S*)-1,4-dihydroxybutyl-2-(6-deoxyglucose) (6dglcBT).

Another particular binding motif found in aminoglycoside–rRNA recognition was described by Westhof *et al.*^8^ The ring I of paromomycin forms a pseudo base pair with adenine 1408 (Fig. 1b). This hydrogen bond pattern was also observed between the bicyclo ring of apramycin and A1408.^14^ Recently, we have placed a 6-deoxyglucose-guanine pair inside a DNA double helix to study this type of interaction.^15^ The fully resolved NMR structure (Fig. 1c and d) showed that two hydrogen bonds are formed between H1 G-O4 6dglc and HN2 G-O3 6dglc. This pseudo base pair caused destabilization within the DNA duplex probably due to the bulky size of the pyranose ring with respect to a natural DNA base (Fig. 2a) and due to the glycerol linkage used to anchor the glucose unit to the phosphodiester DNA skeleton (Fig. 2b and c). However, a certain selectivity of glc and 6dglc to pair with purines was observed possibly due to the formation of two hydrogen bonds whereas only one was formed with pyrimidines.

Our results have opened up the possibility of designing and preparing new DNA base mimics containing non-aromatic scaffolds. The group of aromatic base analogues of natural DNA bases reported is quite large due to the work of Benner's,^16^,^17^ Kool's,^18^,^19^ Romesberg's^20^,^21^ and Hirao's^22^,^23^ groups, but to the best of our knowledge, non-aromatic base mimics have not been reported previously. Carbohydrates, such as the monosaccharide glucose, seem to be a good starting point to investigate non-aromatic base mimics where OH groups will be responsible for H-bonding with the opposite base. To do so, it is necessary to explore the relevance of their connection with the DNA skeleton, the different potential arrangements of the OH groups in order to form hydrogen bonds with the nucleobases, the possible multiple incorporation of sugar-nucleobase pairs in a DNA context and their processing by DNA polymerases. In this work, we have started to tackle several of these aspects. We have synthesized several glucose derivatives (Fig. 2d) and placed them into DNA duplexes in order to confirm the selectivity of glucose for purines, to examine the role of the anchoring unit and the relevance of its stereochemistry, and the incorporation of more than one sugar-nucleobase pair into the DNA duplex.

The influence of the linker on the stability of these pseudo base pairs has been investigated by the comparison of the (*S*)-glycerol linker in glc with its isomeric form, the (*R*)-glycerol linker, in glc(*R*)GY. We have also compared the (*S*)-glycerol linker in 6dglc with a wider version, 2-butanetriol spacer in glc. A permethylated glucose derivative, glc(Me), has been synthesized using the same glycerol linker as in glc and 6dglc. The *O*-methyl groups partially block the ability of the OH groups to form hydrogen bonds since O–Me groups can be H-bond acceptors but cannot be donors. At the same time, *O*-methyl groups present a higher steric hindrance when compared to OH groups. Thus, we would predict a partial loss of selectivity when paired with the natural DNA bases.

We have also solved the NMR structure of double helices containing glc(Me)-G and glc(Me)-T pairs in their interior. This study has allowed us to compare the geometry of these pairs with their analogues 6dglc-G and 6dglc-T in the same double helix context. Moreover, we have carried out quantum chemical calculations on pseudo pairs glc-nucleobase and 6dglc-nucleobase with different geometries of the carbohydrate to explore which sugar edge shows better pairing with the nucleobases. Finally, we have studied the processing of glc and 6dglc as potential DNA base mimics by DNA polymerases. We have performed DNA polymerase insertion experiments opposite glc and 6dglc with different enzymes to examine the potential formation of sugar-nucleobase pairs.

## Results and discussion

### Design and synthesis of glucose nucleobase mimics

The monosaccharide glucose (glc) resembles the coin-like structure of a natural base (Fig. 2a). Moreover, it possesses all its hydroxyl groups in an equatorial configuration and therefore avoids steric clash of axial OH groups with the natural bases above and below the pyranose ring when inside a DNA double helix. Connection of the glucose unit with the phosphodiester backbone of DNA was carried out through a flexible glycerol linker as a first and simple approach.^15^ We have used standard phosphoramidite chemistry for the preparation of the corresponding carbohydrate oligonucleotide conjugates (COCs). This type of conjugation is quite convenient and straight-forward and it has been reported previously for the synthesis of COCs with different applications such as improving cellular uptake of antisense oligonucleotides^24^,^25^ and siRNAs,^26^–^28^ preparation of potential anti-HIV drugs,^29^ investigation of lectin binding to carbohydrates and glycoarrays^30^ and preparation of molecular interaction probes.^11^,^13^,^31^,^32^

**Scheme 1:**
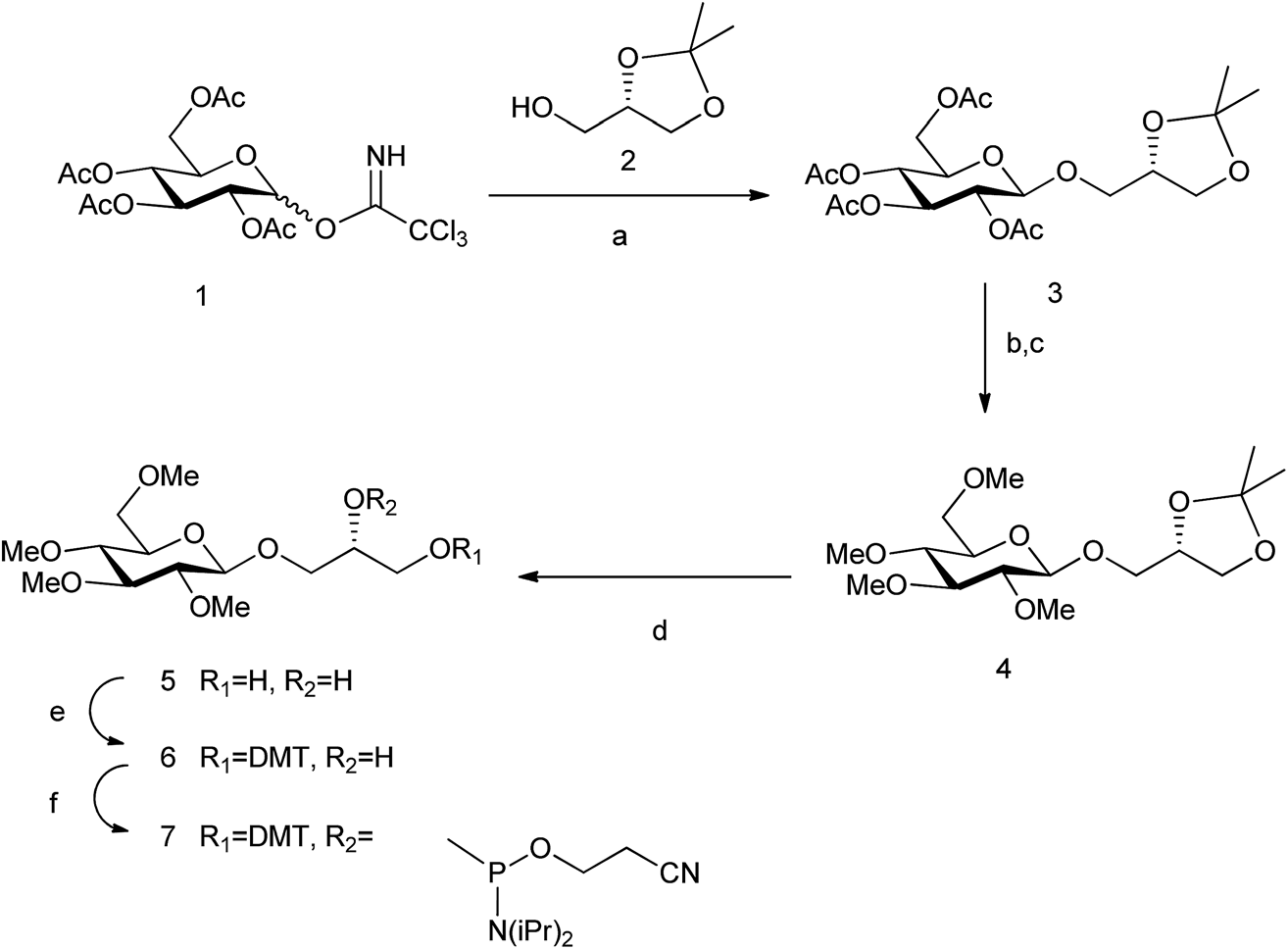
Synthesis of glc(Me) phosphoramidite. Reagents and conditions: (a) BF_3_·OEt_2_, CH_2_Cl_2_, 86%; (b) Na_2_CO_3_, MeOH; (c) MeI, NaH, DMF, 80% (both steps); (d) AcOH–H_2_O, 80 °C, 84%; (e) DMTCl, DMAP, CH_2_Cl_2_, 93%; and (f) 2-cyanoethyl-*N*,*N*-diisopropylamino-chlorophosphoramidite, DIPEA, CH_2_Cl_2_, 90%.

We have now prepared the permethylated version of glc, glc(Me), using the same spacer and chemical methodology (Scheme 1). (*S*)-(+)-1,2-Isopropylideneglycerol **2** was glycosylated using the corresponding trichloroacetimidate donor; then acetyl protecting groups were deprotected and methylation was carried out. Finally, acetal hydrolysis, followed by introduction of the DMT and phosphoramidite groups yielded derivative **7**, successively added in the required position of the oligonucleotide similarly to a standard natural DNA base phosphoramidite.

**Scheme 2:**
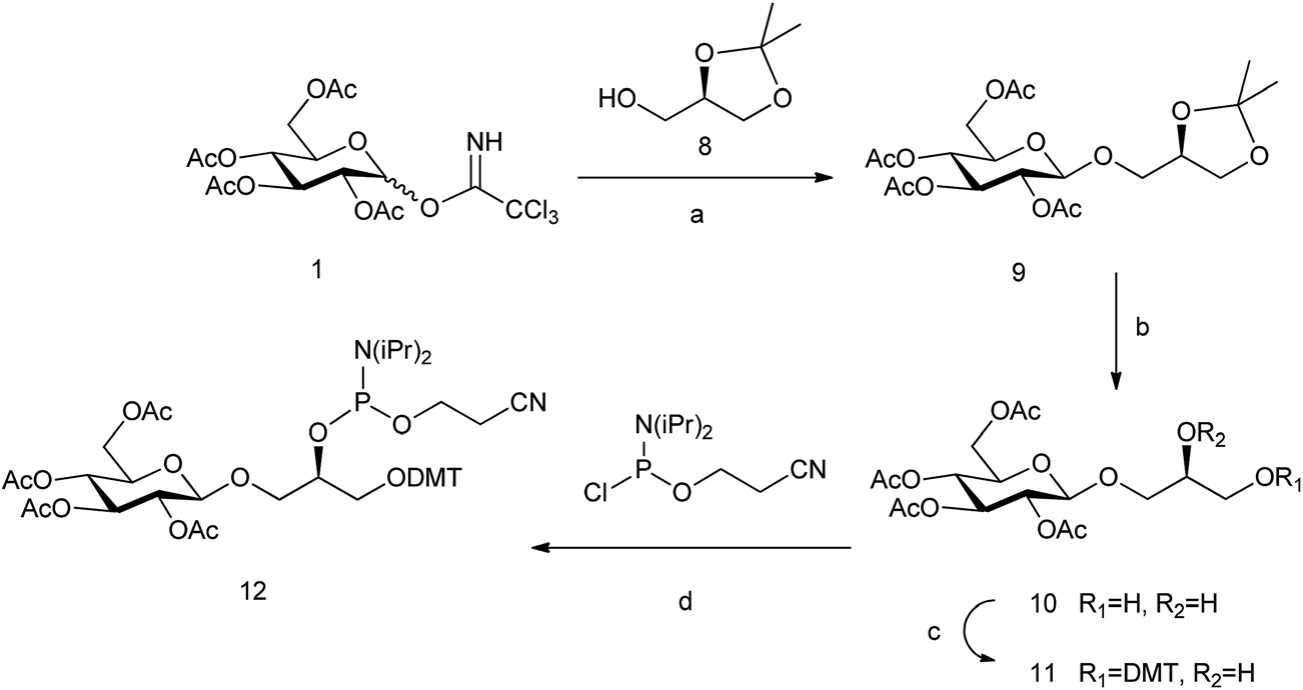
Synthesis of glc(*R*)GY phosphoramidite. Reagents and conditions: (a) BF_3_·OEt_2_, CH_2_Cl_2_, 65%; (b) AcOH–H_2_O, 80 °C, 78%; (c) DMTCl, DMAP, CH_2_Cl_2_, 80%; and (d) 2-cyanoethyl-*N*,*N*-diisopropylamino-chlorophosphoramidite, DIPEA, CH_2_Cl_2_, 85%.

The analogue of glc containing the (*R*)-glycerol linker, the glc(*R*)GY DMT-phosphoramidite derivative **12**, was synthesized following the same methodology reported previously starting from (*R*)-(–)-1,2-isopropylideneglycerol **8** (Scheme 2).^15^

**Scheme 3:**
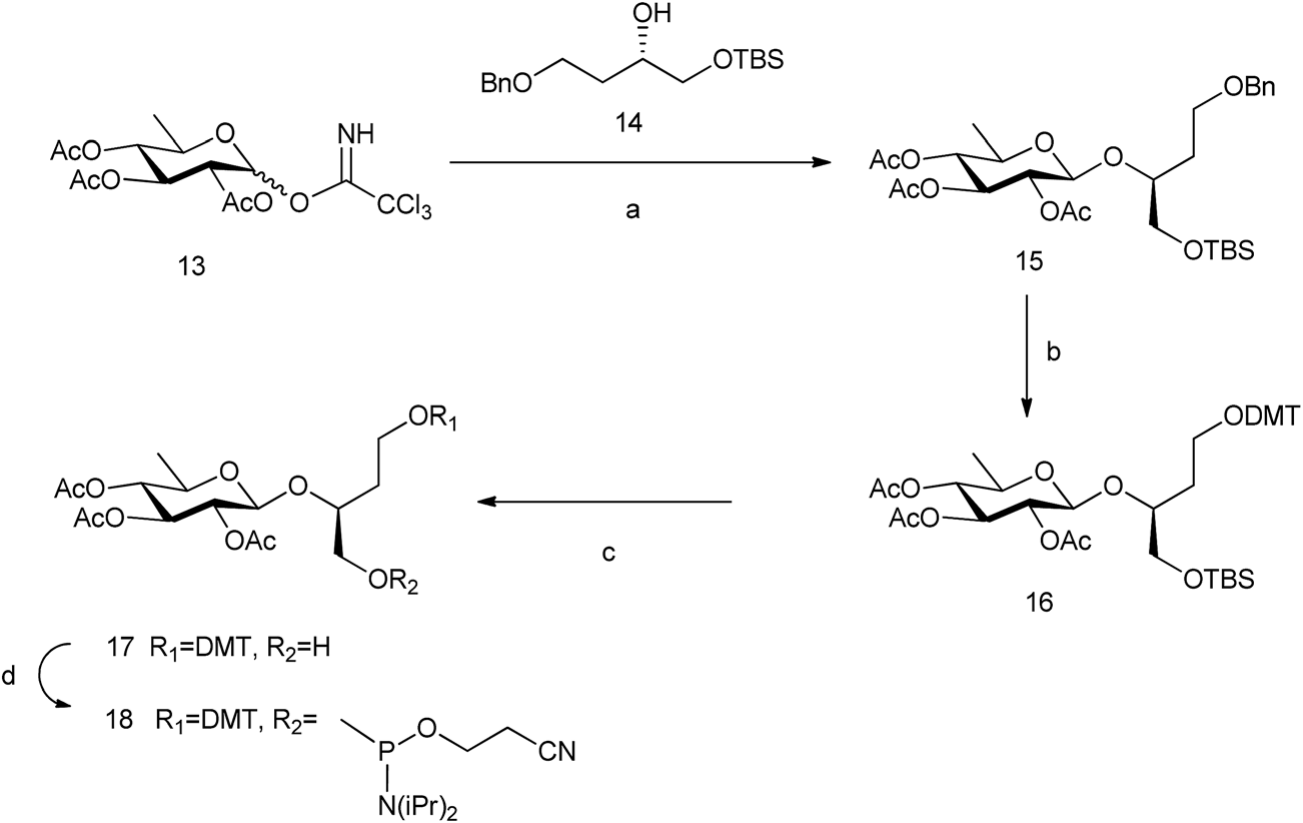
Synthesis of 6dglcBT phosphoramidite. Reagents and conditions: (a) BF_3_·OEt_2_, CH_2_Cl_2_, –10 °C, 63%; (b) H_2_, Pd(OH)_2_, ethyl acetate; DMTCl, DMAP, CH_2_Cl_2_, 84% (both steps); (c) TBAF, THF, 0 °C, 32%; and (d) 2-cyanoethyl-*N*,*N*-diisopropylamino-chlorophosphoramidite, DIPEA, CH_2_Cl_2_, 77%.

In order to study the role of the linker in the stability of these pseudo base pairs, we have prepared 6dglcBT using 1,2,4-butanetriol as a longer spacer than glycerol that could allow the thicker structure of pyranoses within a DNA double helix. (*S*)-(+)-4-Benzyloxy-1[*tert*-butyldimethylsilanyloxy]-butan-2-ol **14** 33 was glycosylated with peracetylated 6-deoxyglucosyl donor **13** to obtain compound **15 **(Scheme 3). Hydrogenation allowed the removal of the benzyl group from the primary OH, which was thus available for introduction of the DMT group. Then, silyl deprotection using TBAF in methanol and reaction with 2-cyanoethyl-*N*,*N*-diisopropylamino-chlorophosphoramidite resulted in compound **18**.

### Thermal stability studies

We have recently reported melting temperatures (*T*_m_) for DNA duplexes containing monosaccharides linked through a flexible glycerol spacer.^15^ When compared to a natural DNA base pair (T-A) in the same DNA context, we observed a decrease in *T*_m_ of 14.7–19.4 °C for pairs of glc and 6dglc with DNA bases (Table 1). Part of this loss in DNA stability can be attributed to the larger volume of the pyranose ring, but most of it seems to be due to the use of a flexible linker. In fact, when we compare natural base T with its flexible derivative T*, a descent of 10.2–15.9 °C in DNA stability was measured.

**Table 1:** Melting temperature (*T*_m_) for DNA duplexes containing T*, sugars linked to DNA through an (*S*)-glycerol (glc, 6dglc and glc(Me)), an (*R*)-glycerol (glc(*R*)GY) or an (*S*)-butanetriol spacer (6dglcBT)^*a*^

DNA duplexes						5′-d(GATGAC**X**GCTAG)^*b*^ ^,^^*c*^ 3′-d(CTACTG**Y**CGATC)					
X-Y^*d*^	*T*_m_	X-Y^*d*^	*T*_m_	X-Y	*T*_m_	X-Y^*d*^	*T*_m_	X-Y	*T*_m_	X-Y	*T*_m_
T*-A	37.7	glc-A	30.6	glc(*R*)GY-A	28.5	6dglc-A	33.2	6dglcBT-A	28.6	glc(Me)-A	32.7
T*-T	32.8	glc-T	28.5	glc(*R*)GY-T	24.9	6dglc-T	29.9	6dglcBT-T	27.0	glc(Me)-T	31.6
T*-C	32.0	glc-C	28.7	glc(*R*)GY-C	27.8	6dglc-C	29.9	6dglcBT-C	27.1	glc(Me)-C	32.8
T*-G	35.6	glc-G	31.3	glc(*R*)GY-G	31.6	6dglc-G	32.7	6dglcBT-G	30.6	glc(Me)-G	32.3
T*-T*	31.1	glc-glc	29.6	glc(*R*)GY-glc(*R*)GY	27.5	6dglc-6dglc	32.0	6dglcBT-6dglcBT	23.5	glc(Me)-glc(Me)	32.0

^*a*^ *T*_m_ values are in °C.

^*b*^The natural DNA duplex containing X-Y = T-A results in a *T*_m_ of 47.9 °C.

^*c*^Conditions for DNA duplexes: 10 mM NaH_2_PO_4_, 150 mM NaCl, pH 7.0. Estimated errors are ±0.4 °C (in DNA, except for 6dglc-6dglc: ±1.0 °C). Average value of three experiments measured at 1.2 μM concentration (DNA).

^*d*^Data from ref. 15.

The influence of the spacer on the DNA stability of sugar-nucleobase pseudo base pairs was investigated. 1,2,4-Butanetriol (BT) was compared to glycerol as a spacer. BT is larger than glycerol and allows a four atom separation between two phosphates in the DNA skeleton which could possibly accommodate the wider pyranose ring. However, *T*_m_ values decreased by 2.1–4.6 °C for duplexes containing 6dglcBT-nucleobase pairs with respect to 6dglc-nucleobase pairs (Table 1). The extra separation between phosphate groups in the DNA skeleton may distort the duplex structure, and the higher degrees of freedom in the BT spacer could also be a cause of the observed duplex destabilization. In fact, the effect of the longer BT spacer is more evident when comparing 6dglc-6dglc pairs with 6dglcBT-6dglcBT pairs, with a reduction in *T*_m_ of 8.5 °C. It is important to mention that selectivity for purines observed for glc and 6dglc (1.9 to 3.3 °C more stable than pairs with pyrimidines) is maintained for the new derivative 6dglc BT (1.6 to 3.6 °C more stable when pairing purines than pyrimidines). More rigid and conformationally constrained linkers such as natural deoxyribose or locked ribose derivatives may help avoid this loss in DNA thermal stability.

Then, we studied the effect of replacing glc with glc(*R*)GY where the (*R*)-glycerol spacer would modify the DNA skeleton geometry and would change the location of the monosaccharide inside the DNA duplex. The glc(*R*)GY-nucleobase pairs decreased the DNA stability when compared to glc-nucleobase pairs (0.9–3.6 °C) except for the glc(*R*)GY-G pair which showed a similar *T*_m_ value to that of the glc-G pair (Table 1). This result could be due to the existence of three donor or acceptor groups in guanine that could still make two hydrogen bonds with glucose even when glucose changes its original geometry in the glc-G pair. In the case of adenine this nucleobase is more limited with respect to changes in H-bond geometry since it only possesses two donor or acceptor groups.

We also measured the thermal stability of DNA duplexes containing the permethylated glucose derivative glc(Me) which uses the same glycerol linker as glc and 6dglc (Table 1). In this case, all glc(Me)-nucleobase pairs display similar *T*_m_ values (32.3–32.8 °C) except the glc(Me)-T pair which is slightly lower (31.6 °C). The selectivity for purines observed in all the glucose derivatives (glc, 6dglc and 6dglcDB) is lost for glc(Me) pairs as expected. The lack of hydroxyl groups and the bulky methyl groups on glc(Me) probably hinder the formation of hydrogen bonds that leads to the selection of purine bases on glc, 6dglc and 6dglcBT. In fact, other hydrophobic DNA base mimics such as Kool isosteric nonpolar DNA bases^34^ also show this lack of selectivity when paired with natural bases since they are not capable of hydrogen bond formation.

Finally, we examined thermal stability of GNA–DNA chimeras containing several 6dglc-nucleobase pairs in the internal GNA region. We had previously observed that a GNA duplex with a single 6dglc-nucleobase pair inside decreased its stability by 11.9 °C with respect to the GNA duplex.^15^ However, the selectivity displayed by this type of pair (6dglc-purine *vs.* 6dglc-pyrimidine) was much higher in GNA than in DNA. Since the wide pyranose ring of the monosaccharide could be highly disrupting the helix structure, we decided to incorporate two or four contiguous and alternating 6dglc-nucleobase pairs trying to counteract this effect. In all cases, the DNA–GNA chimeras were less stable than the corresponding natural DNA duplex (Table 2). Surprisingly, the chimera containing two 6dglc-A pairs was 4.3 °C more stable than the one with two T-A GNA pairs. However, we found the opposite effect when comparing chimeras with four pairs where the one containing four T-A GNA pairs was 27.4 °C more stable than that containing four alternating 6dglc-A pairs. Quantum chemical calculations are warranted to shed some light on the possible cause of this effect. It is also important to note that the previously observed selectivity for purines for 6dglc is also observed when incorporating two sugar-nucleobase pairs.

**Table 2:** Melting temperature (*T*_m_) for DNA–GNA chimeric duplexes containing sugars linked to DNA through an (*S*)-glycerol spacer and GNA nucleobases

DNA–GNA chimeras			
5′-d(GACTGA***XY***CCTGCG)^*a*^ ^,^^*b*^ ^,^^*c*^ 3′-d(CTGACT***YX***GGACGC)		5′-d(GACTG***XYXY***CTGCG)^*a*^ ^,^^*b*^ 3′-d(CTGAC***YXYX***GACGC)	
X-Y	*T*_m_ (°C)	X-Y	*T*_m_ (°C)
6dglc-*A*	45.3	6dglc-*A*	22.4
*T-A*	41.0	*T-A*	49.8
6dglc-*T*	39.8	—	—
6dglc-6dglc	32.0	—	—

^*a*^The natural DNA duplex containing XY = TA results in a *T*_m_ of 55.9 °C.

^*b*^X and Y are sugar derivatives or GNA monomers and are shown in italics.

^*c*^Conditions for DNA–GNA chimeric duplexes: 10 mM NaH_2_PO_4_, 150 mM NaCl, pH 7.0. Estimated errors are ±0.4 °C. Average value of three experiments measured at 1.2 μM concentration.

### Structural studies

**Fig. 3:**
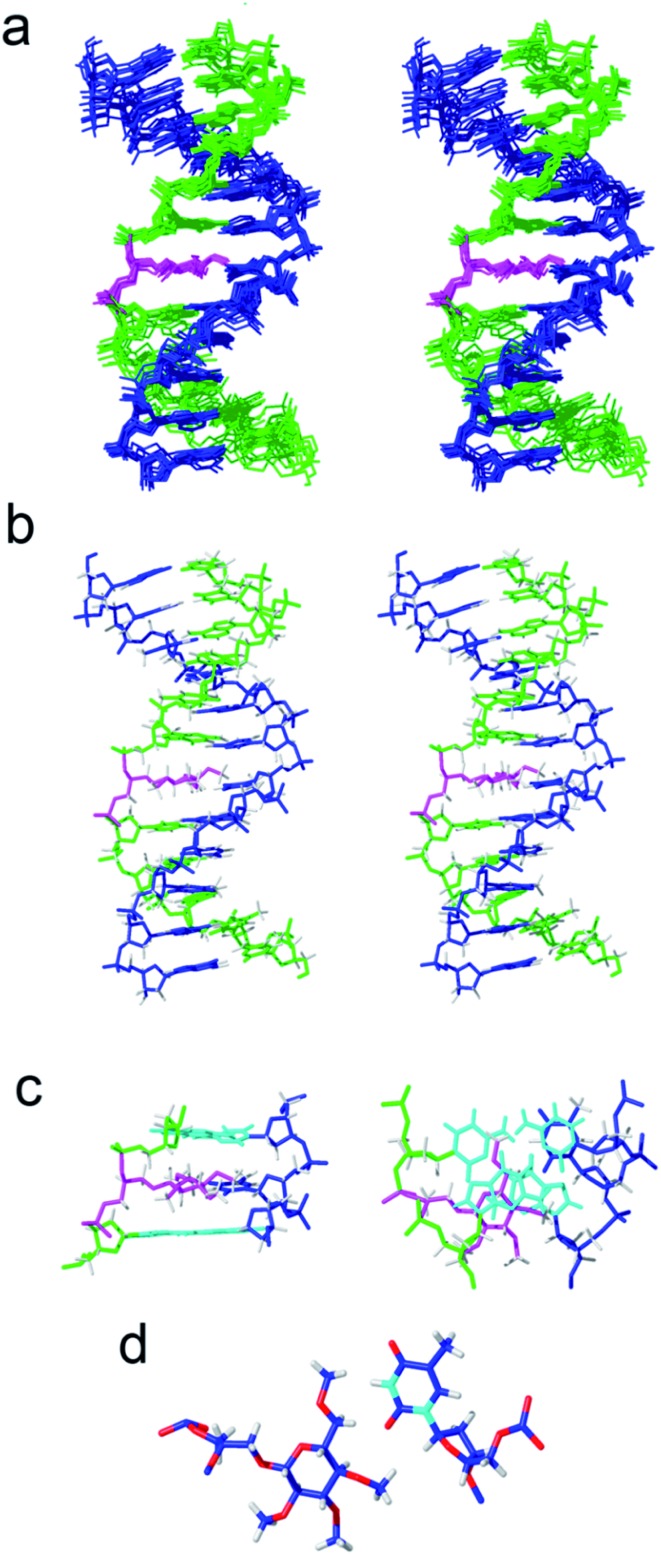
Solution structure of helix glc(Me)-T. The corresponding oligonucleotide sequences are 5′-GATGACTGCTAG and 3′-CTACTG-glc(Me)-CGATC. (a) Stereoscopic view of the ensemble of the 10 refined structures, (b) stereoscopic view of a representative structure. Color code: modified strand in green; complementary strand in blue; carbohydrate and linker in magenta; and hydrogen atoms in grey. (c) Two views showing details of the carbohydrate moiety and the surrounding base-pairs. (d) Detail of the interaction between the apolar carbohydrate and the opposite thymine.

Next, we investigated the effect of inserting an apolar carbohydrate moiety in a DNA duplex and compared it with the effect of inserting a natural sugar. We were also interested in finding out if the lack of selectivity observed for glc(Me) could be due to the presence of this bulky carbohydrate inside the helix that could be expelling the opposite base from the interior of the helix. The three-dimensional structures of helix glc(Me)-G and helix glc(Me)-T (Fig. 3 and ESI†) were determined by restrained molecular dynamics methods based on experimental NMR distance constraints. In both cases, the exchangeable proton region of the NMR spectra exhibited 11 imino proton signals between 12.5 and 14.5 ppm, a clear indication of the formation of a double helix with Watson–Crick base pairs (Fig. S3†).

The nucleotide located opposite to the apolar sugar showed an imino signal in the region of 10–11 ppm. All protons of the DNA, apolar carbohydrate units and the spacers were completely assigned with only a few exceptions (Tables S1 and S2†) following standard ^1^H NMR techniques. A comparison between the DNA chemical shifts in the conjugates and the natural DNA control duplexes revealed that the changes are restricted to the bases around the permethylated carbohydrate derivatives, showing low distortion of the double helix structure (Fig. S4†).

Both three-dimensional structures obtained are well-defined B-form helices (Fig. 3b, S6 and S7†). The apolar sugars and the opposing nucleobases (G or T) locate inside the DNA helix intercalating between the base-pairs above and below presenting extensive contacts. Accordingly, a large number of NOE cross-peaks (Fig. S5 and Table S3†) between the spacer and the permethylated glucose protons with the DNA are observed. The only exception is helix glc(Me)-G where only three carbohydrate–DNA NOEs were observed. As is commonly observed in intercalation complexes, both double helices are slightly unwound and the rise between flanking residues is increased.

**Fig. 4:**
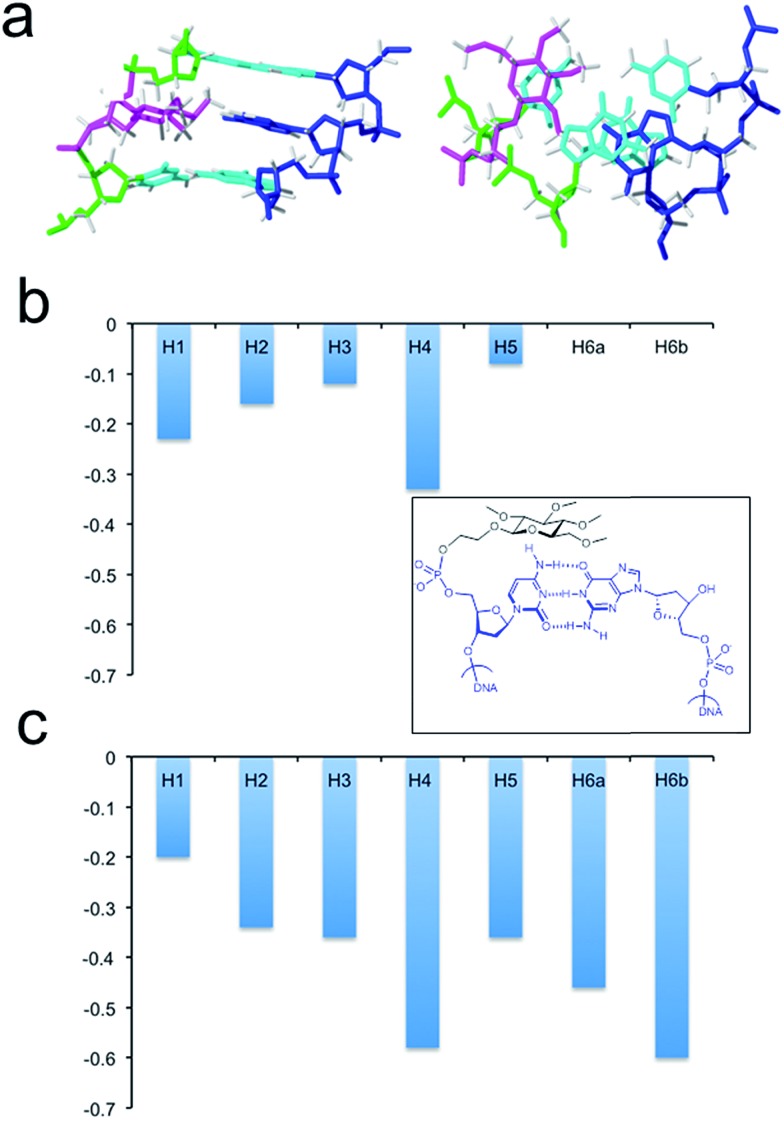
(a) Solution structure of helix glc(Me)-G. The corresponding oligonucleotide sequences are 5′-CTAGCGGTCATC and 3′-GATCG-glc(Me)-CAGTAG. Two views showing details of the carbohydrate moiety and the surrounding base-pairs. (b) Chemical shift differences of glc(Me) protons on helix glc(Me)-G with respect to control duplex glc(Me)-CGCGCG (inner picture). (c) Chemical shift differences of glc(Me) protons on helix glc(Me)-T with respect to control glc(Me)-CGCGCG duplex (inner picture).

The larger size of the permethylated carbohydrate in comparison with hydroxylated glc and 6dglc causes more distortions in the surrounding base pairs than in helices containing 6dglc-G and 6dglc-T pairs,^15^ but these distortions are not dramatic. The opposite nucleobase remains inserted in the duplex and the carbohydrate is slightly shifted towards the minor groove in helix glc(Me)-T and towards the major groove in helix glc(Me)-G (Fig. 3c and 4a). This difference may be due to the larger size of the guanine base located in the opposite position. In fact, the apolar carbohydrate in helix glc(Me)-G shows less stacking with the surrounding nucleobases (Fig. 4a) when compared to the stacking in helix glc(Me)-T (Fig. 3c). Chemical shift differences of glc(Me) when placed inside helix glc(Me)-G or helix glc(Me)-T with respect to control duplex glc(Me)-CGCGCG (Fig. 4b and c) support these differences in stacking observed in the structures of helix glc(Me)-G and helix glc(Me)-T. These differences in stacking do not correspond to the similar thermal stability observed in these two conjugates possibly because it is compensated for by a stronger stacking of G *versus* T.

### Quantum chemical calculations

In our previous work, we computationally explored our pseudo base pairs glc-X and 6dglc-X (where X was a natural DNA base) using dispersion-corrected density functional theory (DFT) at the BLYP-D3(BJ)/TZ2P level of theory.^15^ In that case, the monosaccharide was considered as if it was attached to the DNA skeleton through its anomeric (OH1) position leaving mainly OH3, OH4 and OH6 available for hydrogen bonding with the natural DNA base. We observed that the binding energies of sugar-purine pairs were in the same range of an A-T base pair (Table 3). All these pairs showed the formation of two or three H-bonds with the corresponding purine base.

**Table 3:** Hydrogen-bond energies (in kcal mol^–1^) of sugar-nucleobase pairs in the gas-phase (Δ*E*_gas_) and in aqueous solution (Δ*E*_water_)^*a*^

Attachment position	X-Y	Δ*E*_gas_	Δ*E*_water_	X-Y	Δ*E*_gas_	Δ*E*_water_
	A-T	–18.5	–9.4	G-C	–34.0	–13.5
1	6dglc-G	–23.8	–10.5	glc-G	–23.3	–12.2
	6dglc-T	–10.5	–6.1	glc-T	–15.4	–9.5
	6dglc-A	–16.7	–10.5	glc-A	–16.7	–10.7
	6dglc-C	–12.9	–6.7	glc-C	–17.7	–5.1
2	6dglc-G	–11.3	–5.1	glc-G	–18.2	–6.1
	6dglc-T	–2.1	–0.3	glc-T	–9.0	–1.3
	6dglc-A	–10.7	–8.1	glc-A	–14.0	–9.5
	6dglc-C	–13.2	–4.7	glc-C	–20.3	–5.2
3	6dglc-G	–3.1	–2.2	glc-G	–11.3	–4.2
	6dglc-T	–15.6	–2.3	glc-T	–21.0	–3.1
	6dglc-A	–8.16	–3.2	glc-A	–7.5	–4.1
	6dglc-C	–12.6	–4.0	glc-C	–20.0	–3.4
4	6dglc-G	–21.7	–10.1	glc-G	–27.6	–12.6
	6dglc-T	–15.0	–9.6	glc-T	–17.5	–5.8
	6dglc-A	–12.4	–8.8	glc-A	–13.8	–8.9
	6dglc-C	–15.7	–6.1	glc-C	–15.4	–7.1
6				glc-G	–20.1	–8.8
				glc-T	–15.5	–9.0
				glc-A	–12.4	–9.1
				glc-C	–18.3	–6.2

^*a*^Calculated at the BLYP-D3(BJ)/TZ2P level of theory using COSMO to simulate aqueous solution.

In this work, we have computed the potential binding of glc and 6dglc when attached to the DNA skeleton through other position than OH1. This was done again at the BLYP-D3(BJ)/TZ2P level of theory as implemented in the ADF program.^35^ This study has allowed us to investigate which edge of the pyranose would bind more efficiently the natural bases through hydrogen bonding. When the carbohydrate was attached to the skeleton through position OH1 and OH4, the binding energies of glc-X and 6dglc-X pairs were much higher than with the sugar attached through OH2, OH3 and OH6. In fact, the binding energies of glc-nucleobase pairs when the sugar is linked through the anomeric position (OH1) or through OH4 are in the same range of a calculated A-T base pair.

In relation to selectivity, while 6dglc-X pairs linked through OH1 showed preference for purines in water, selectivity for adenine was observed when attached through OH2, and for guanine when attached through OH4. In the case of glc-X pairs, selectivity for purines was also observed, with preference for guanine when glucose was attached through OH1 and OH4, preference for adenine when the attachment was through OH2 and no clear preference when linked through OH6.

**Fig. 5:**
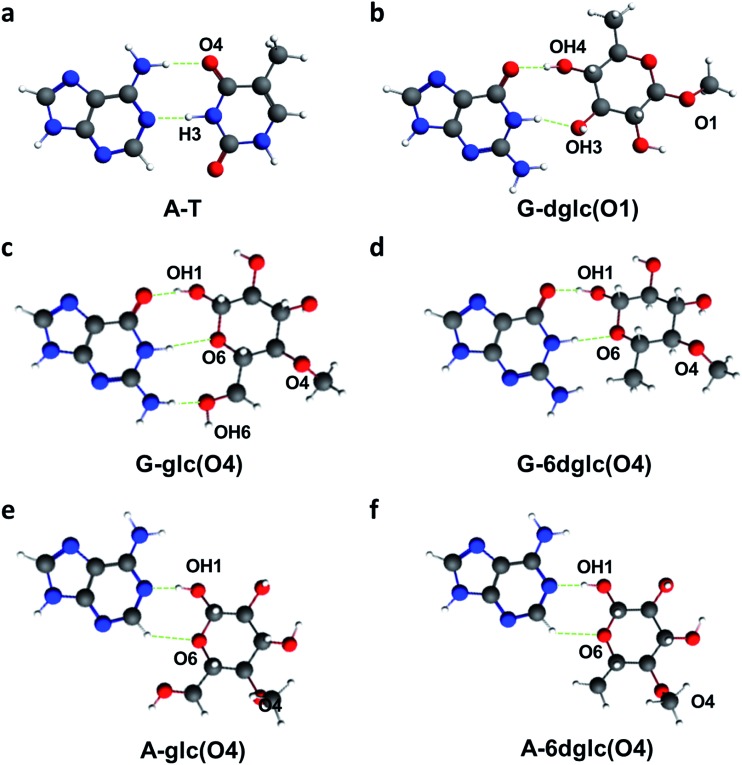
A-T, G-6dglc(O1), G-glc(O4), G-6dglc(O4), A-glc(O4) and A-6dglc(O4) pairs calculated at the BLYP-D3(BJ)/TZ2P level of theory using COSMO to simulate aqueous solution. H-bonds are shown in green.

In general, high H-bonding energies correspond to sugar-nucleobase pairs with two or three hydrogen bonds where the monosaccharide and the nucleobase arrange in the same plane as in the Watson–Crick base pairs. In fact, we had already observed the geometric similarities between A-T and G-6dglc attached through OH1 (Fig. 5a and b). Likewise, when attached to the skeleton through OH4, 6dglc and glc form two or three hydrogen bonds to G, respectively, with a quite planar disposition (Fig. 5c and d). Interestingly, A-6dglc and A-glc pairs show a potential hydrogen bond that occurs through adenine H2 (Fig. 5e and f). It is important to note that all the other pairs studied tend to form one or no hydrogen bonds and their pairing geometry is out of plane.

### DNA polymerase insertion studies

**Fig. 6:**
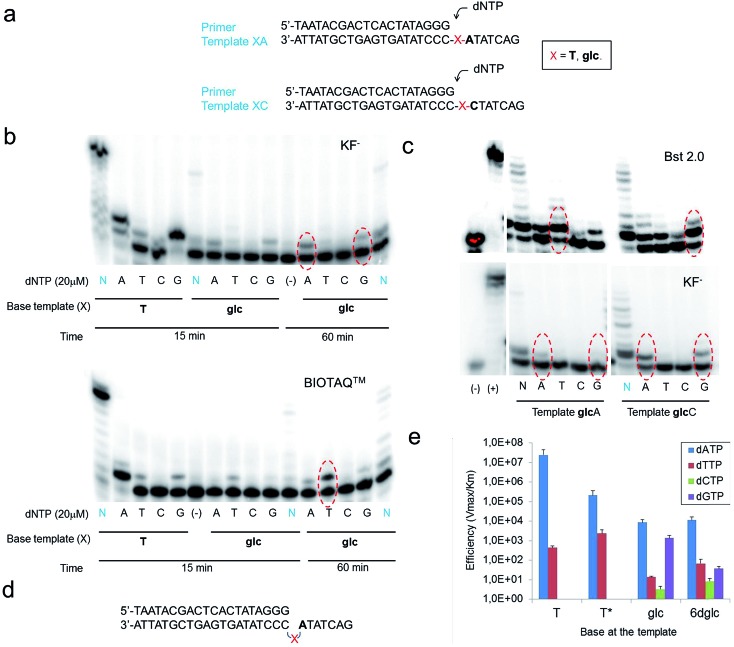
Primer single-nucleotide insertion and extension experiments opposite glc. (a) Primer and template sequences used where X = T or glc; dNTP stands for dATP, dTTP, dCTP and dGTP that are added individually (single insertion) or together (multiple insertion). (b) Denaturing polyacrylamide gels showing single nucleotide insertions with glc in the template strand XA using the four natural nucleotides. N stands for dNTP which means deoxynucleotide triphosphates; thus, all four nucleotides are present in those reactions. The data correspond to KF^–^, 37 °C using 0.2 units per μl KF^–^ (Klenow fragment (exo-)) and BIOTAQ™, 37 °C using 0.25 units BIOTAQ™ polymerase, using in all cases 5 μM primer–6 μM template duplex, 20 μM dNTP and the reactions were stopped after 15 or 60 minutes, as indicated. Red ovals indicate the line where single-nucleotide insertion is occurring with the highest efficiency. (c) Denaturing polyacrylamide gels showing single nucleotide insertions opposite glc in templates glcA and glcC using the four natural nucleotides. The data correspond to KF^–^, 37 °C using 0.2 units per μl KF^–^, Bst 2.0, 55 °C using 0.4 units per μl Bst 2.0, using in both cases 5 μM primer–6 μM template duplex, 20 μM dATP/dTTP/dCTP/dGTP, 100 μM dNTP and the reactions were stopped after 60 minutes. (d) Schematic representation of the possible loop-out mechanism to bypass the carbohydrate nucleobase. X represents the carbohydrate nucleobase modification. (e) Histogram of efficiency for nucleotide insertion of KF^–^ opposite T, T*, glc and 6dglc using a primer-template XC as shown in (a).

Five DNA polymerases (KF^–^, SIII, BIOTAQ™, Bst 2.0 and Therminator™) were screened to examine single-insertion of natural nucleotides opposite glc (Fig. 6a) in a DNA template. Despite the thicker size of a pyranose ring and the presence of OH groups in glc, selective insertion opposite glc was observed (Fig. 6b, S9 and S10†). The only exception was Therminator™ that inserted any nucleotide opposite glc and even opposite natural T in accordance with the low fidelity reported for this polymerase. Surprisingly, while KF^–^ inserted dATP (up to 20%) and also dGTP to some extent opposite glc, the other three DNA polymerases SIII, BIOTAQ and Bst 2.0 inserted dTTP (up to 50% for Bst 2.0) and then dATP and dGTP to some extent (Fig. 6b, S9 and S10†).

Potentially different interactions and spatial constraints at the binding site could be argued to explain the different selectivities found. Another possibility is that a loop-out mechanism could be operating since the following base in the template sequence after glc is an A (Fig. 6d). This mechanism is only known for the Y-family DNA polymerases, low-fidelity polymerases that replicate damaged DNA.^36^,^37^ It consists of the bending of the DNA to leave the undesired position outside the replication line. Then, the polymerase inserts the corresponding nucleotide opposite the following 3′ base after the lesion.

We investigated the possibility of a loop-out mechanism operating using templates in which the 3′ base next to glc was C instead of A (Fig. 6c). KF^–^ polymerase presented the same insertion pattern in both templates. In the case of Bst 2.0 and BIOTAQ™ the insertion preference changes from dTTP to dGTP with some insertion of dATP and dTTP (Fig. 6c and S10†). It seems that a loop-out mechanism is at least partially operating for these two polymerases. The fact that glc presents a flexible glycerol linker within the DNA skeleton may facilitate this mechanism in high fidelity polymerases such as Bst 2.0 and BIOTAQ™ although it has only been reported in low fidelity DNA polymerases.

Quantitative single-nucleotide insertion studies of natural nucleotides opposite T, T*, glc and 6dglc were carried out under steady-state conditions using KF^–^ polymerase. Results showed that dATP is preferentially inserted over T* but less efficiently than over natural T by a factor of 100 (Fig. 6e and Table S6†). Thus, the change of a deoxyribose for a flexible spacer in T* decreased considerably dATP incorporation. dATP was the best inserted nucleotide by KF^–^ opposite glc and 6dglc, only 20 fold less efficiently than opposite T* and more efficiently than mismatches T-T and T-T*. A notable selectivity was observed for insertion opposite 6dglc where dATP is preferentially inserted over the other three natural nucleoside triphosphates by a factor of 170–1400. The selectivity is similar for insertion opposite glc (a factor of 600–2600) except for dGTP which was inserted only slightly worse than dATP most probably due to the possibility of the third potential hydrogen bond through OH6 as observed in the theoretical calculations.^15^

When we carried out multiple nucleotide insertion experiments, all DNA polymerases showed a pause after the first insertion opposite the glc and 6dglc nucleobase mimics and some extension to the end of the strand could be observed in all cases (Fig. 6b and c, S9 and S10†). In fact, KF^–^ polymerase is capable of significantly extending templates T*C and 6dglcC to full-length DNA (Fig. S11†). The presence of the pyranose ring may produce a distortion in the DNA double helix hindering proper binding by the DNA polymerase to further process the modified DNA. Pausing extension could also be caused by incorrect formation of hydrogen bonds between the DNA minor groove and the DNA polymerase needed for DNA processing.^38^

Directed evolution of KF^–^ polymerase could lead to mutants with improved substrate binding and catalytic efficiency capable of managing sugar DNA base mimics. These tools would open up new possibilities to incorporate alternative DNA bases into non-standard oligonucleotides that will expand the information storage capability of natural DNA. Indeed, this sort of polymerase evolution has been performed successfully by Holliger *et al.*^39^ to replicate simple nucleic acid architectures not found in nature, such as anhydrohexitol nucleic acids (HNAs) or α-l-threofuranosyl nucleic acids (TNAs). Benner's group has also evolved polymerases that can support PCR amplification with external primers containing two nonstandard nucleotides, 2-amino-8-(1-β-d-2′-deoxyribofuranosyl)imidazo[1,2-*a*]-1,3,5-triazin-4(8*H*)-one (trivially called P) that pairs with 6-amino-5-nitro-3-(1-β-d-2′-deoxyribofuranosyl)-2(1*H*)-pyridone (trivially called Z).^40^

To the best of our knowledge, only one example of DNA synthesis of a nucleobase linked through a flexible linker to DNA has been reported. In this case, enzymatic synthesis of full-length DNA on a dodecamer GNA template was found to proceed with very low efficiency when using Bst 2.0 DNA polymerase.^41^ Only the use of diaminopurine triphosphate and MnCl_2_ instead of MgCl_2_ improved considerably DNA synthesis with Bst 2.0 and also with Superscript II reverse transcriptase.

## Conclusions

Although carbohydrate interactions with DNA and RNA are of vital importance in the mechanism of action of antibiotics, the effect of carbohydrate derivatives inside DNA duplexes is an unexplored field. Our DNA model system containing a glucose unit mimicking a nucleobase has opened the possibility of including non-aromatic DNA bases into DNA oligomers. In this study, we have confirmed that glucose-nucleobase pairs show selectivity for purines when inside a DNA double helix independently of the type of linker used. When we explored new anchoring units for attaching glc to the phosphodiester skeleton such as butanetriol to allow more space for the pyranose rings, we did not obtain any gain in thermal stability. This result indicates the requirement of a more conformationally restricted linker to improve the overall duplex stability.

When glc(*R*)GY which contains the isomeric (*R*)-glycerol linker was incorporated into DNA, we observed similar thermal stability in comparison with glc-X pairs but better selectivity. Glc-nucleobase pairs were more stable with purines but the glc(*R*)GY-G pair was the more stable of this series. Adenine may be more limited with respect to H-bonding when the sugar disposition changes since it only possesses two donor or acceptor groups whereas guanine possesses three.

We have also observed that apolar glucoside-nucleobase pairs (glc(Me)-X) show similar stability to that of our previous glucoside-nucleobase pairs (glc-X). However, glc(Me)-nucleobase pairs showed no selectivity for purines or pyrimidines possibly due to the fact that hydrogen bonds cannot be easily formed as in glc-nucleobase pairs. Structural studies by NMR showed that the nucleobase opposite to glc(Me) is not kicked out of the DNA duplex structure; it stays fully inside but the steric hindrance and low accessibility of the OMe groups do not allow the formation of hydrogen bonds.

Our quantum chemical calculations on possible glc-nucleobase and 6dglc-nucleobase pairs indicate that only if the sugars are attached through OH1 or OH4 to the DNA skeleton their binding energies seem to be high enough to obtain stable sugar-nucleobase pairs. Moreover, although glc and 6dglc showed selectivity to bind purines when attached through OH1, this changes to only A or only G (for glc) and to T and A (for 6dglc) when attached through OH4 or OH6.

Finally, KF^–^ polymerase inserted A and G to some extent opposite glc and 6dglc with notable selectivity. It was even capable of fully extending the newly formed pair especially in the case of 6dglc in the template XC. Polymerase evolution could lead to mutants with improved catalytic efficiency and selectivity capable of processing non-aromatic nucleobases such as glc or 6dglc. On the other hand, Bst 2.0, SIII and BIOTAQ™ DNA polymerases seem to display, at least partially, a loop-out mechanism when trying to replicate glc or 6dglc. The fact that the sugars are attached through a flexible linker instead of a deoxyribose must be critical in this case.

## Conflicts of interest

There are no conflicts of interest to declare.

## Supplementary Material

Supplementary information
