# Supplementary material for: Glucose-nucleobase pairs within DNA: impact of hydrophobicity, alternative linking unit and DNA polymerase nucleotide insertion studies
Source: Chem Sci. 2018 Mar 5;9(14):3544–54. doi: 10.1039/c7sc04850e (PMC5934746; doi:10.1039/c7sc04850e)
Supplement: Supplementary file 1 [file SC-009-C7SC04850E-s001.pdf]

Electronic Supplementary Material (ESI) for Chemical Science.
This journal is © The Royal Society of Chemistry 2018

Glucose-Nucleobase Pairs within DNA: Impact of Hydrophobicity, Alternative Linking Unit and DNA Polymerase Nucleotide Insertion Studies.

Empar Vengut-Climent, Pablo Peñalver, Ricardo Lucas, Irene Gómez-Pinto, Anna Aviñó, Alicia M. Muro-Pastor, Elsa Galbis, M. Violante de Paz, Célia Fonseca Guerra, F. Matthias Bickelhaupt, Ramón Eritja, Carlos González and Juan Carlos Morales*

Supplementary Information

Contents:

Supplementary materials and methods

- Synthesis materials and methods 3
- Synthesis of **glc(Me), glc(R)GY**, **6dglcBT** and phosphoramidites 3-22
- ^{1}H and ^{13}C-NMR spectra of new compounds 3-22
- Synthesis of natural and modified oligonucleotide DNA strands 22
- HPLC chromatograms of modified oligonucleotide DNA strands. 23
- Maldi-TOF mass of modified oligonucleotide DNA strands. 25
- Synthesis of oligonucleotide DNA-GNA chimeric strands 25
- HPLC chromatograms of oligonucleotide DNA-GNA chimeric strands. 26
- Maldi-TOF mass of oligonucleotide DNA-GNA chimeric strands. 26
- Thermal denaturation methods 27
- NMR methodology 27
- Structure Calculations 28
- DFT quantum chemical methods 28
- DNA polymerase primer insertion and extension reactions 29
- Steady-state kinetics 30

Supplementary Figures

- Figure S1: Melting curves for DNA double helices containing **6dglcBT** and **glc(Me)** nucleobase mimics. 31
- Figure S2. Melting curves for DNA-GNA chimeric strands. 32
- Figure S3. Imino región of the NMR spectra of **helix glc(Me)-G** and **helix glc(Me)-T** 33
- Figure S4. More significant changes in proton chemical shifts along the sequence for **helix glc(Me)-G** and **helix glc(Me)-T** 33-34
- Figure S5. Regions of NOESY spectra of **helix glc(Me)-T** in D_2O 35
- Figure S6. Solution structure of **helix glc(Me)-T** 36
- Figure S7. Solution structure of **helix glc(Me)-G** 37

- Figure S8. Structures for pairs containing **glc** and **6dglc** in *vacuo* and in water, computed at BLYP-D3(BJ)/TZ2P using COSMO to simulate aqueous solution. 38-39
- Figure S9. Gels showing DNA polymerase insertion opposite **T** and **glc** (DNA polymerases used: SIII, Bst 2.0 and Therminator) 40
- Figure S10. Gels showing DNA polymerase insertion opposite **glcA** and **glcC** templates (DNA polymerase used: BIOTAQ) 41
- Figure S11. Gel showing DNA polymerase extension opposite **T**C, **T***C, **glc** and **6dglcC** templates (DNA polymerase used KF^-) 41

Supplementary Tables

- Table S1: ^{1}H-NMR assignments of **helix glc(Me)-T**. 42
- Table S2: ^{1}H-NMR assignments of **helix glc(Me)-G**. 43
- Table S3. Structurally relevant carbohydrate-DNA NOE contacts for **helix glc(Me)-G** and **helix glc(Me)-T** 44
- Table S4. NMR structural constraints and calculation statistics. 45
- Table S5. Cartesian coordinates and ADF total energies of all stationary points in this study, computed at BLYP-D3(BJ)/TZ2P using COSMO to simulate aqueous solution. 45-69
- Table S6. Steady-State Kinetics for Single Nucleotide Insertions with KF^- Polymerase opposite **T**, **T***, **glc** and **6dglc** in the DNA template. 70

Supplementary references 70

Synthesis materials and methods

All chemicals were obtained from chemical suppliers (Sigma-Aldrich, Carbosynth) and used without further purification, unless otherwise noted. Anhydrous dichloromethane was dried in an aluminium oxide column machine, PURESOLV (Scharlab). Other anhydrous solvents were dried over molecular sieves (4 Å) for 48 h.

All reactions were monitored by TLC on precoated silica gel 60 plates F_{254} (Merck) and detected by heating after staining with H_2SO_4:EtOH (1:9, *v/v*), anisaldehyde (450 ml ethanol, 25 ml anisaldehyde, 25 ml H_2SO_4 and 1 ml AcOH) or Mostain (500 ml of 10% H_2SO_4, 25g of $(NH_4)_6Mo_7O_{24}$•$4H_2O$, 1g $Ce(SO_4)_2$•$4H_2O$). Products were purified by flash chromatography with silica gel 60 (200-400 mesh). Eluents are indicated for each particular case.

NMR spectra were recorded on either a Bruker Avance 300 or ARX 400 MHz or Bruker Avance DRX 500 MHz [300, 400 or 500 MHz (^{1}H), 75, 100, 125 MHz (^{13}C), at room temperature for solutions in $CDCl_3$ or CD_3OD] spectrometer. Chemical shifts are referred to the solvent signal and are expressed in ppm. 2D NMR experiments (COSY, TOCSY and HMQC) were carried out when necessary to assign the corresponding signals of the new compounds. Low resolution electrospray mass spectral analyses were obtained on a Bruker Esquire 6000 ion trap mass spectrometer. High resolution FAB (+) mass spectral analyses were obtained on a Micromass AutoSpec-Q spectrometer.

Synthesis of glcBT, 6dglcBT and glc(Me) phosphoramidites

General procedure for isopropylidene removal. A solution of the 2,2-dimethyldioxolane derivative (1.93 mmol) was dissolved in 40 ml of acetic acid 80% and the solution was stirred at 80 ºC for 1 to 2 hours. The solvent was removed and coevaporated with toluene. The reaction crude was then purified by flash chromatography.

General procedure for primary hydroxyl protection with 4,4-dimethoxytritylgroup. DIPEA (1.941 mmol) and DMAP (0.097 mmol) were added to a solution of the unprotected compound (0.971 mmol) in pyridine/CH_2Cl_2 (1:1, 10 ml). The temperature was lowered to 0 ºC and DMTCl (1.456 mmol) was slowly added. The reaction was stirred for 15 minutes at 0 ºC and 3 h at room temperature. Then, reaction was stopped by adding MeOH and the solvent was removed in *vacuo*. The reaction mixture was purified by flash chromatography.

General procedure for synthesis of carbohydrate phosphoramidites. To a solution of DMT protected compound (0.546 mmol) in dry CH_2Cl_2 (5 ml), DIPEA (0.382 ml, 2.185 mmol) and 2-cyanoethyl-*N*,*N*'-diisopropylamino-chlorophosphoramidite (0.183 ml, 0.819 mmol) were added at room temperature under argon atmosphere. After 1h the solvent was evaporated to dryness. The

product was purified by silica gel column chromathography using a mixture of hexane, ethyl acetate and triethylamine.

(*S*)-2,2-dimethyl-4-(2,3,4,6-tetra-*O*-methyl-β-D-glucopyranos-1-ylmethyl)dioxolane (4)

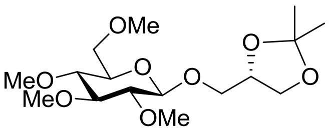

4

(*S*)-2,2-dimethyl-4-(2,3,4,6-tetra-*O*-acetyl-β-D-glucopyranos-1-ylmethyl)dioxolane **3**[1] (0.759 g, 1.641 mmol) was dissolved in dry MeOH (30 ml) and Na_2CO_3 (41.4 mg, 0.492 mmol) was then added. The reaction mixture was stirred for 2 h and IR-120 was added to neutralize. Solvent was removed and the crude was used for next step without any further purification. To a solution of the latter crude (521 mg, 1.770 mmol) in anhydrous DMF (50 ml) at 0ºC, NaH (255 mg, 10.62 mmol) was added. The reaction mixture was stirred for 10 min and MeI (0.886 ml, 14.16 mmol). After 24 h isopropanol was added dropwise and NH_4Cl sat (50 ml). The combined organic phases were extracted with ethyl acetate (2x200 ml) and washed with sodium bisulfate solution (100 ml) and brine (100 ml). The crude was purified by silica gel column chromathography using as eluent (Hex/EtOAc, from 1:1 to 1:3) to give **4** (505 mg, 80%) as a syrup.^{1}H NMR (400 MHz, $CDCl_3$) δ (ppm): 4.28-4.23 (m, 1H, -$CH_{\text{-isopropylidene}}$), 4.21 (d, *J* =7.6 Hz, 1H, H_1), 4.03-3.99 (m, 1H, -CH*H*O-), 3.93 (dd, *J* =4.4/10.0 Hz, 1H, -C*H*HO-), 3.84-3.80 (m, 1H, -CH*H*O-), 3.62-3.57 (m, 1H, H_6), 3.58 (s, 3H, -OCH_3), 3.53-3.43 (m, 2H, $H_{6'}$, -C*H*HO-), 3.58, 3.52, 3.48, 3.36 (4s, 12H, 4x-OCH_3), 3.25-3.19 (m, 1H, H_5), 3.13-3.07 (m, 1H, H_3, H_4), 2.97-2.93 (m, 1H, H_2), 1.37, 1.31 (2s, 6H, -$C(CH_3)_2$) ^{13}C NMR (101 MHz, $CDCl_3$) δ (ppm): 109.3 (-*C*$(CH_3)_2$), 103.7 (C_1), 86.4, 83.6, 79.2, 74.6, 74.4, 74.3, 71.2, 70.0, 66.8, 60.8, 60.4, 60.4, 59.3, 26.8, 26.7, 25.3. HRMS (FAB$^+$) Calcd. for $C_{16}H_{30}NaO_8$ (M+Na): 373.1838, found; 373.1844.

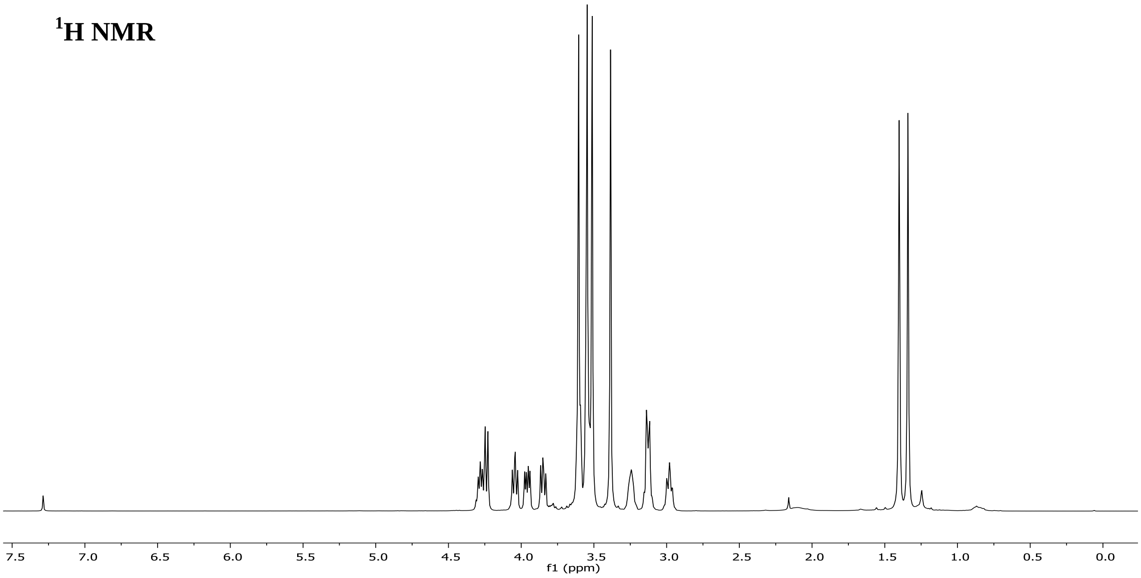

^{13}C NMR

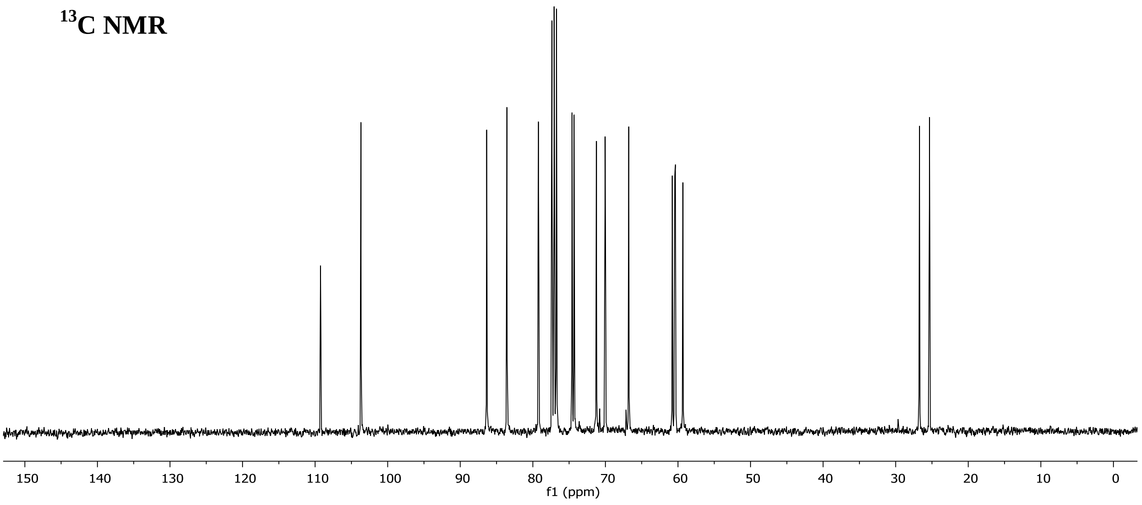

(*R*)-2,3-dihydroxypropyl 2,3,4,6-tetra-*O*-methyl-β-D-glucopyranoside (5)

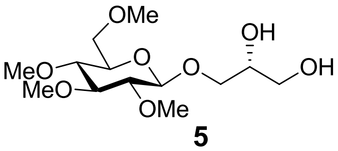

5

Compound **4** (314 mg, 0.896 mmol) was dissolved in a mixture of CH_3COOH/H_2O (15 ml, 4;1). The reaction mixture was reacted following the general isopropylidene removal procedure. The crude was purified by silica gel column chromathography (EtOAc/MeOH, from 1:0 to 10:1) to give **5** (234 mg, 84%) as a syrup. ^{1}H NMR (400 MHz, $CDCl_3$) δ (ppm): 4.26 (d, J =8.0 Hz, 1H, H_1), 3.89-3.75 (m, 3H, -CH_2O-, -CH-), 3.70-3.51 (m, 2H, H_6, $H_{6'}$), 3.61, 3.57, 3.51, 3.39 (4s, 12H, -OCH_3), 3.35-3.32 (m, 1H, C*H*HO-) 3.18-2.98 (m, 6H, H_5, H_3, H_4, H_2, -CH*H*O-), 1.37, 1.31 (2s, 6H, -$C(CH_3)_2$). ^{13}C NMR (101 MHz, $CDCl_3$) δ: 103.9 (C_1), 86.4, 83.5, 79.5, 76.7, 74.2, 73.3, 71.4, 71.1, 70.9, 63.4, 60.8, 60.5, 60.4, 59.2. HRMS (FAB^+) Calcd. for $C_{13}H_{26}NaO_8$ (M+Na): 333.1525, found; 333.1538.

^{1}H NMR

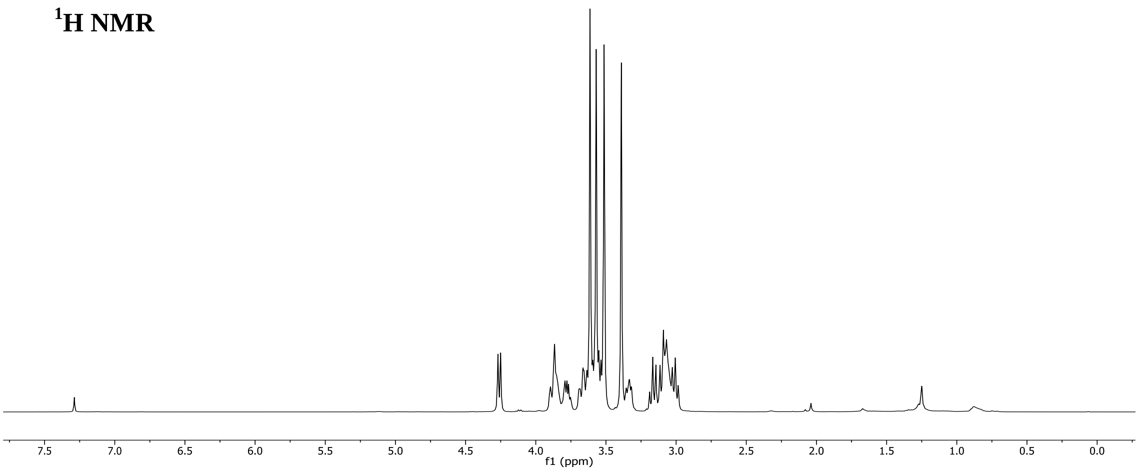

^{13}C NMR

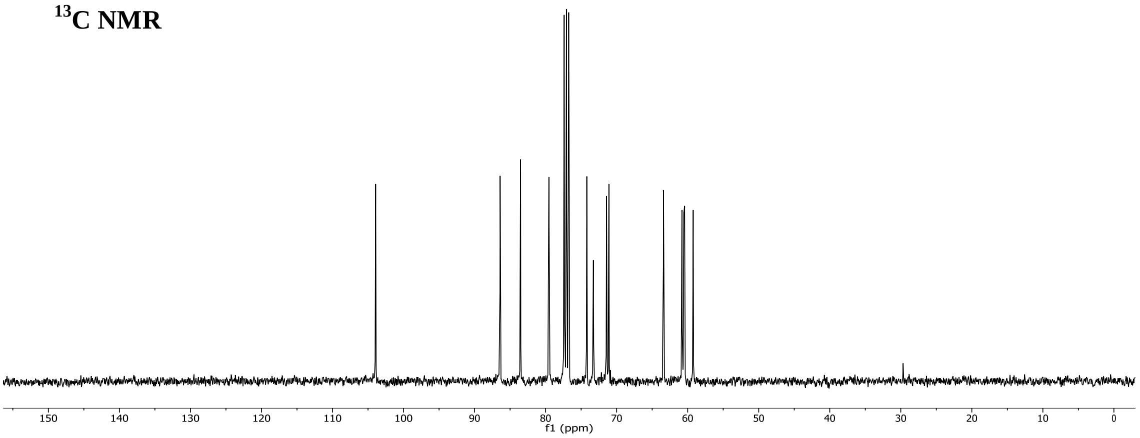

(*R*)-3-(4,4'-Dimethoxytrityloxy)-2-hydroxyproyl 2,3,4,6-tetra-*O*-methyl-β-D-glucopyranoside (6).

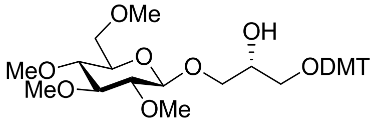

6

To a solution of compound **5** (209 mg, 0.673 mmol) in dry pyridine-CH_2Cl_2 (1:1, 6 ml) was reacted following the general procedure for hydroxyl protection. The crude was purified by silica gel column chromathography (Hex/EtOAc, from 2:1 to 1:2) to give **6** (384 mg, 93%) as a syrup. ^{1}H NMR (400 MHz, $CDCl_3$) δ (ppm): 7.34-7.28 (m, 6H, H_{arom}), 7.20-7.18 (m, 3H, H_{arom}), 6.86-6.82 (m, 4H, H_{arom}), 4.25 (d, 1H, *J*= 8.0 Hz, H_1), 3.97-3.81 (m, 1H, -CH-), 3.87-3.69 (m, 10H, H_6, $H_{6'}$, -CH_2O-, 2x-OCH_3), 3.62, 3.57, 3.51, 3.39 (4s, 12H, -OCH_3), 3.39-3.32 (m, 2H, -CH_2O-) 3.21-2.95 (m, 4H, H_5, H_3, H_4, H_2). ^{13}C NMR (101 MHz, $CDCl_3$) δ (ppm): 158.6, 147.3, 139.5, 130.1, 129.1, 127.8, 127.1, 113.2, 104.1 (C_1), 86.5, 83.6, 81.4, 79.6, 74.4, 73.7, 71.5, 71.1, 63.4, 60.9, 60.5, 59.3, 55.2. HRMS (FAB$^+$) Calcd. for $C_{34}H_{34}O_{10}Na$ (M+Na): 635.2848, found; 635.2832.

^{1}H NMR

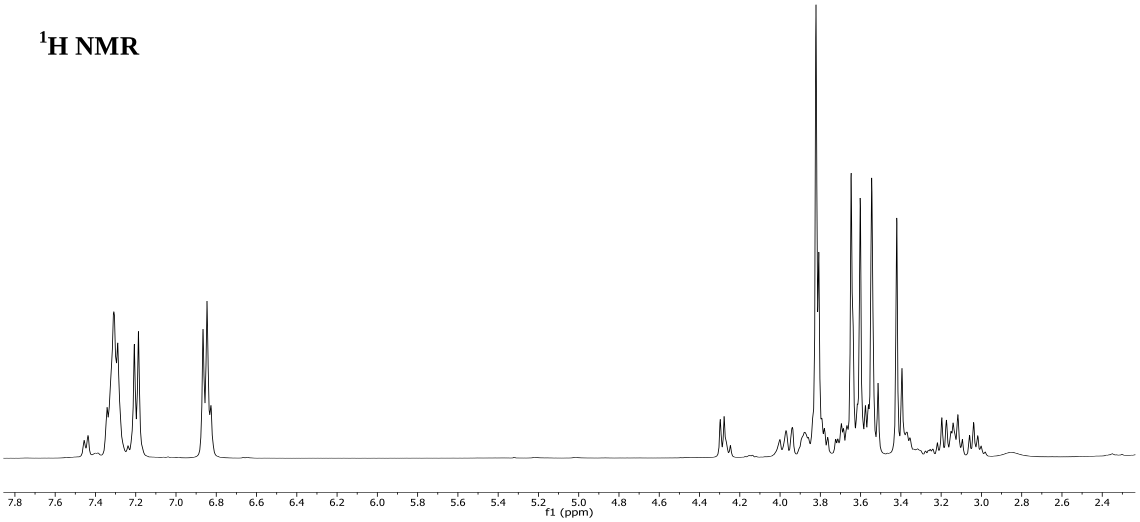

^{13}C NMR

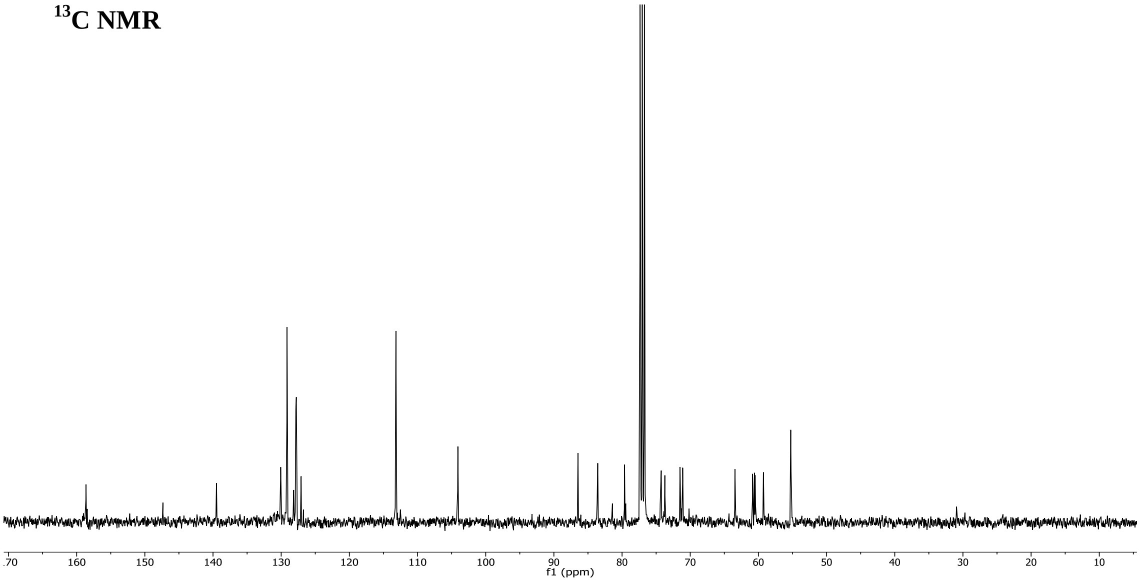

2-(*R*)-1-(4,4'-dimethoxytrytiloxy)-3-(2,3,4,6-tetra-*O*-methyl-β-D-glucopyranosyloxy)propyl (2-cyanoethyl) (*N*,*N'*-diisopropyl) phosphoramidite (7)

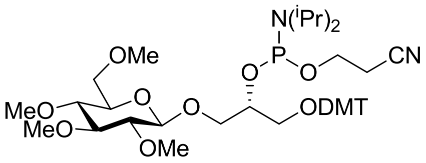

7

A solution of compound **6** (150 mg, 0.245 mmol) in anhydrous CH_2Cl_2 (5 ml) was reacted following the general procedure for phosphoramidite synthesis. The crude was purified by silica gel column chromatography (Hex/EtOAc, 3:2 with 5% of NEt_3) to give compound **7** (180 mg, 90%) as a colourless syrup. ^{1}H NMR (400 MHz, $CDCl_3$) δ (ppm) (mix of isomers): 7.49-7.45 (m, 2H, H_{arom}), 6.37-7.26 (m, 6H, H_{arom}), 7.24-7.19 (m, 1H, H_{arom}), 6.83 (t, 4H, *J* =7.2 Hz, H_{arom}), 4.27-4.14 (m, 2H, $H_{1,}$ -CH-), 4.12-3.86 (m, 2H, -O*CH*$_2$CH$_2$CN), 3.85-3.74 (m, 8H, 2x-OCH_3, -CH_2O-), 3.67-3.54 (m, 10H, 2x-OCH_3, H_6, $H_{6',}$ 2x-C*H*(CH_3)$_2$), 3.31-34 (m, 6H, 2x-OCH_3), 3.28-3.10 (m, 5H, H_3, H_4, $H_{5,}$ -CH_2O-), 2.93-2.87 (m, 1H, H_2), 2.68-2.64 (m, 1H, -*C*H*H*CN), 2.48-2.43 (m, 1H, -*CH*HCN), 1.35-1.07 (m, 12H, 4x-$CH_{3isopropyl}$).; ^{13}C NMR (101 MHz, $CDCl_3$) δ (ppm): 158.4, 144.9, 136.2, 136.1, 130.2 (2), 130.1, 129.1, 128.3, 128.2, 127.7, 126.6, 118.0, 117.7, 113.0, 103.5, 103.4, 86.3, 86.2, 86.0, 85.9, 83.7, 83.6, 79.3, 76.7, 74.6, 74.5, 72.6, 72.4, 71.3, 70.2, 69.7, 64.5, 64.0, 60.8, 60.5, 60.3, 59.4, 59.3, 58.4, 55.2, 43.2, 43.1, 24.7, 24.7, 24.6, 24.5, 20.4, 20.2, 20.2. ^{31}P NMR (162 MHz, $CDCl_3$) δ (ppm): 149.9, 149.6. HRMS (FAB$^+$) Calcd. for $C_{34}H_{34}O_{10}Na$ (M+Na): 835.3911, found; 835.3978.

^{1}H NMR

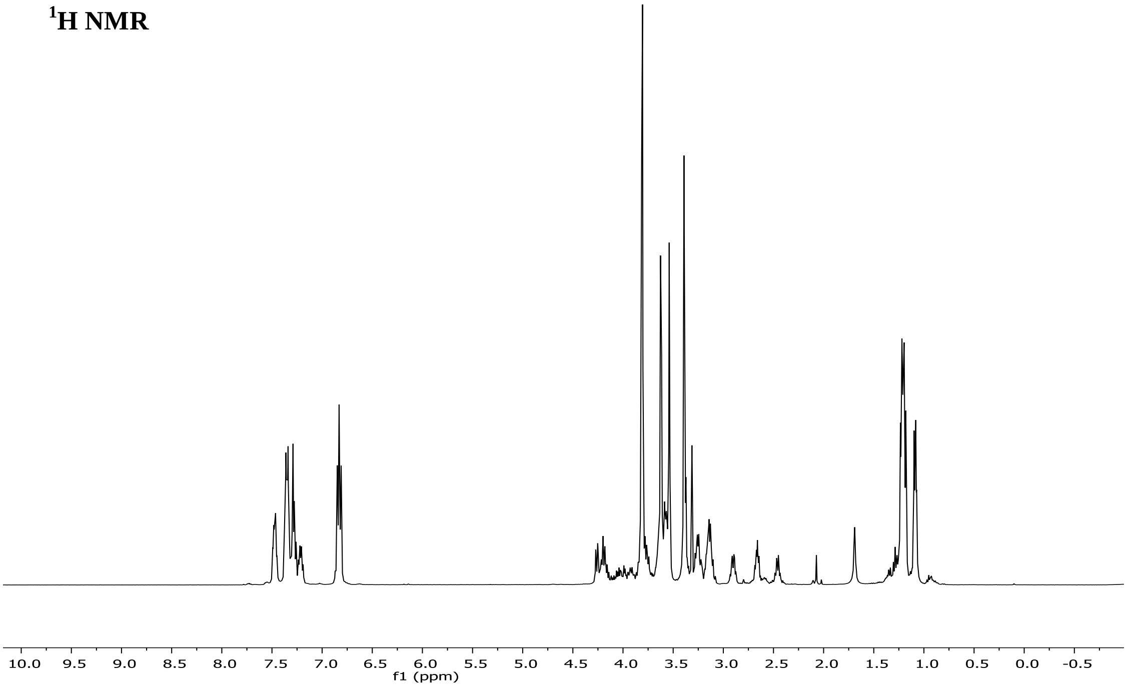

^{13}C NMR

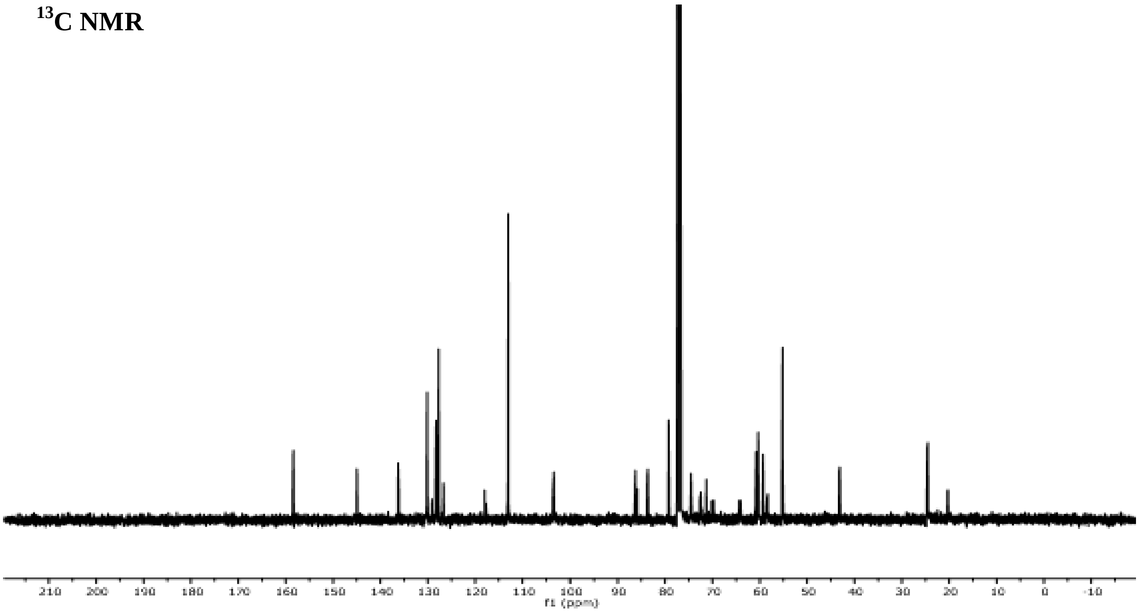

^{31}P NMR

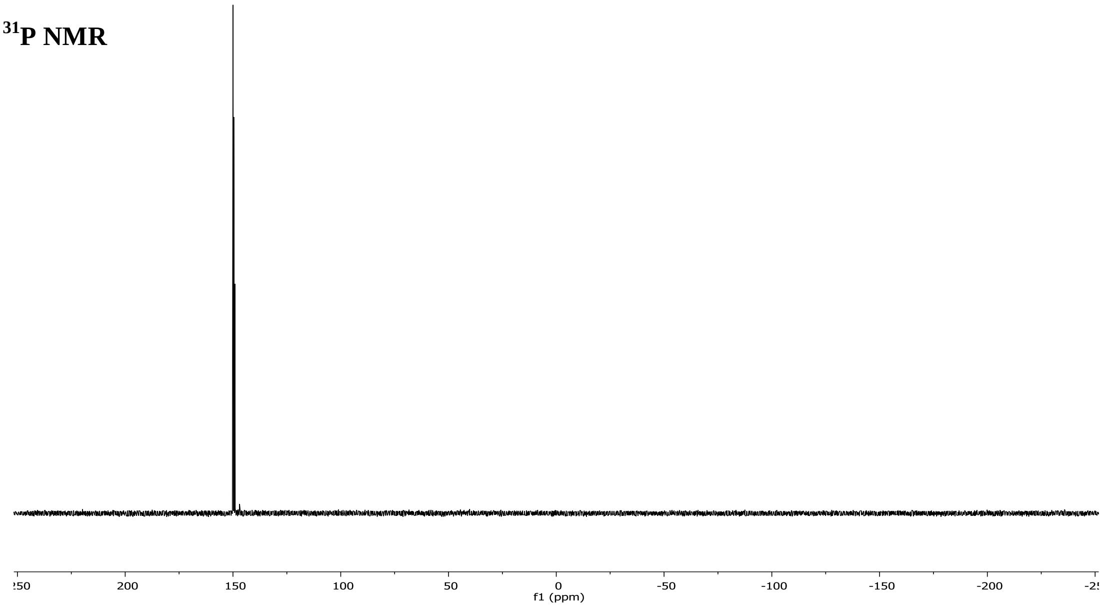

(*R*)-2,2-dimethyl-4-(2,3,4,6-tetra-*O*-acetyl-β-D-glucopyranos-1-ylmethyl)dioxolane (9)

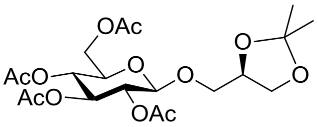

9

To a solution of 2,3,4,6-tetra-O-acetyl-α,β-D-glucopyranosyl trichloroacetimidate **1** (2.3 g, 4.67 mmol) and (*R*)-(+)-1,2-isopropylideneglycerol **8** (1.75 mL, 14 mmoL) in anhydrous CH_2Cl_2 (20 mL) was added $BF_3.OEt_2$ (58 µl, 0.47 mmol). The reaction was then stirred for 30 min and NEt_3 (0.5 mL) was then added. The solvent were then removed and crude was purified by flash column chromatography (hexane: ethyl acetate from 2:1 to 1:2) to afford **9** (1415 mg, 65%); ^{1}H NMR (300 MHz, $CDCl_3$) δ ppm : 5.14 (t, 1H, *J* = 9.44 Hz), 5.01 (t, 1H, *J* = 9.4 Hz), 4.92 (t, 1H , *J* = 9.2 Hz), 4.52 (d, 1H, *J* = 7.8 Hz), 4.25 – 4.12 (m, 2H), 4.12 – 4.02 (m, 1H), 4.01 – 3.90 (m, 1H), 3.83 – 3.50 (m, 5H), 2.02, 1.98, 1.96, 1.94 (4s, 12H, 3 x $OCOCH_3$), 1.34, 1.27 (2s, 6H, $C(CH_3)_2$); ^{13}C NMR (100 MHz, $CDCl_3$): δ = 170.6, 170.2, 169.4, 169.3, 109.5, 100.8, 74.4, 72.7, 71.8, 71.1, 70.5, 68.4, 66.7, 61.9, 26.7, 25.3, 20.7, 20.63, 20.59, 20.57; HRMS (FAB$^+$) Calcd. for $C_{20}H_{30}O_{12}Na$: (M+Na) 485.1635, found; 485.1623.

^{1}H NMR

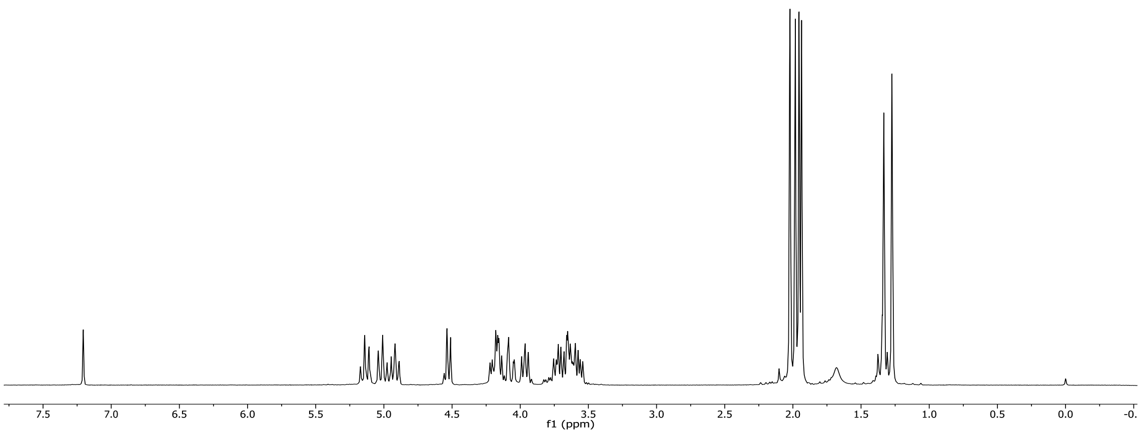

^{13}C NMR

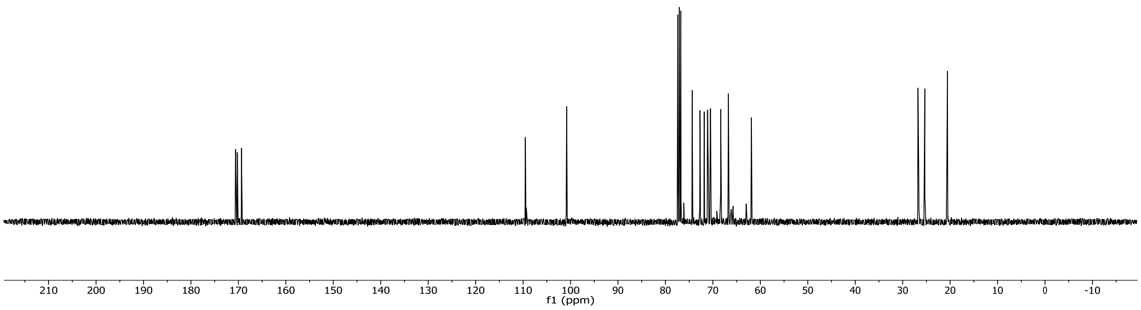

(*S*)-2,3-dihydroxypropyl 2,3,4,6-tetra-*O*-acetyl-β-D-glucopyranoside (10)

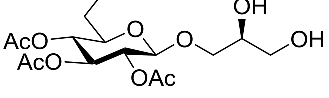

10

Compound **9** (950 mg, 2.05 mmol) was dissolved in a mixture of CH_3COOH/H_2O (10 mL, 4;1). The reaction mixture was stirred for 2 h at 80 ºC. Solvent was then removed and the mixture was co-evaporated with toluene. The crude was purified by silica gel column chromathography using as eluent

(Hexane:AcOEt 1:6 to AcOEt:MeOH 10:1) to give **10** (670 mg, 78%) as a syrup. ^{1}H NMR (400 MHz, $CDCl_3$) δ ppm : 5.22 (t, 1H, *J* = 9.5 Hz,), 5.07 (t, 1H, *J* = 9.7 Hz), 5.00 (t, 1H , *J* = 9.7 Hz), 4.56 (d, 1H, *J* = 7.9 Hz, 1H), 4.32 – 4.05 (m, 2H), 3.91 – 3.56 (m, 6H), 2.68 (br. S, 1H, OH), 2.10, 2.07, 2.04, 2.01 (4s, 12H, 3 x $OCOCH_3$); ^{13}C NMR (101 MHz, $CDCl_3$): δ = 170.7, 170.2, 169.6, 169.4, 101.2, 72.6, 71.9, 71.88, 71.3, 70.5, 68.3, 63.3, 61.9, 60.4, 20.7, 20.6; HRMS (FAB^+) Calcd. for $C_{17}H_{26}O_{12}Na$: (M+Na) 445.1322, found; 445.1320.

^{1}H NMR

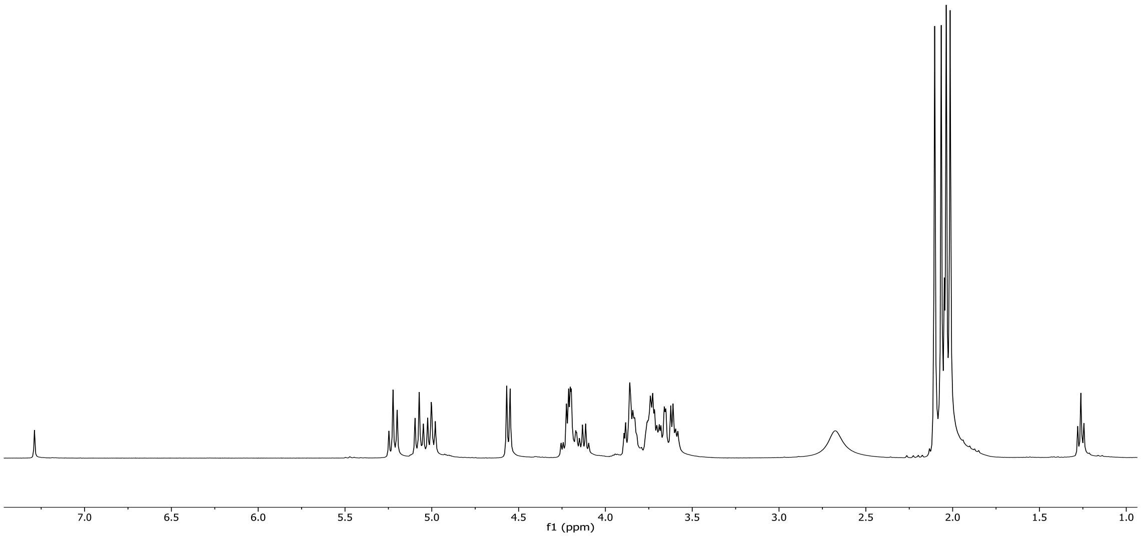

^{13}C NMR

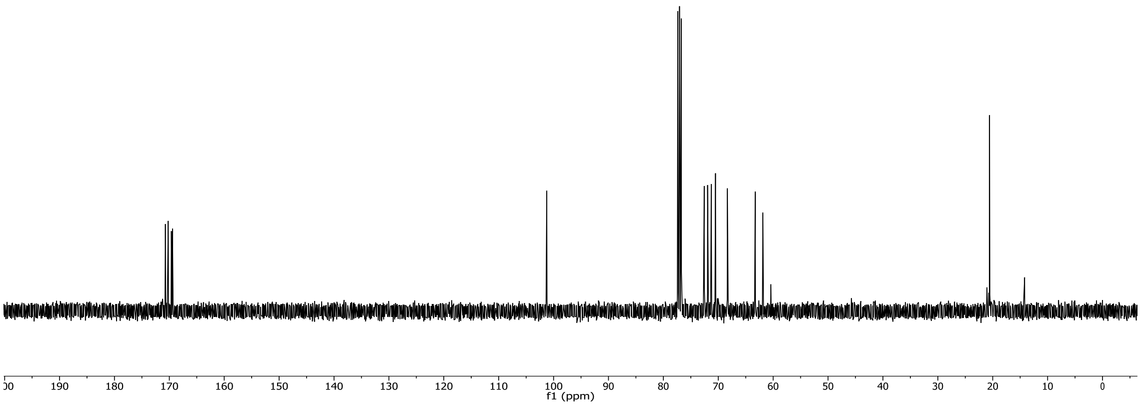

(*S*)-3-(4,4'-dimethoxytrityloxy)-2-hydroxypropyl 2,3,4,6-tetra-*O*-acetyl-β-D-glucopyranoside (11)

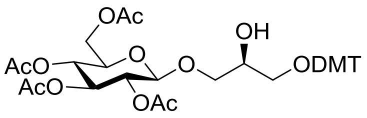

11

To a solution of compound **10** (270 mg, 0.64 mmol) in dry CH_2Cl_2-Py (1:1, 10mL) was added DIPEA (167 μL, 0.96 mmol), DMAP (7.8 mg, 0.06 mmol) and DMTCl (281 mg, 0.83 mmol). The reaction mixture was stirred at 0ºC for 30 min and then 2 h at room temperature. MeOH was then added, the solvents were removed and the crude was purified by silica gel column chromathography using as eluent (Hex:ethyl acetate 1:1-1:3) to give **11** (360 mg, 80%) as a syrup. ^{1}H NMR (400 MHz, $CDCl_3$) δ ppm : 7.44 (d, 2H, *J* = 8.0 Hz), 7.36 – 7.20 (m, 7H), 6.85 (d, 2H, *J* = 8.0 Hz), 5.22 (t, 1H, *J* = 9.5 Hz), 5.08 (t, 1H, *J* = 9.6 Hz), 5.00 (t, 1H, *J* = 9.6 Hz), 4.56 (t, 1H, *J* = 8.0 Hz), 4.27 – 4.09 (m, 2H), 3.98 – 3.86 (m, 2H), 3.81 (2 s, 6H), 3.80-3.70 (m, *2*H), 3.26 – 3.10 (m, 2H), 2.07, 2.05, 2.03, 2.00 (4s, 12H, 4 x $OCOCH_3$); ^{13}C NMR (101 MHz, $CDCl_3$): δ = ^{13}C NMR (101 MHz, $CDCl_3$) δ 170.6, 170.2, 169.4, 169.35, 158.5, 144.8, 135.9, 130.0 (2C), 128.1, 128.05, 127.85, 126.8, 113.2(C2), 101.4, 101.3, 86.15, 72.7, 72.3, 71.85, 71.3, 69.8, 68.4, 63.9, 61.9, 60.4, 55.2, 20.7, 20.6; HRMS (FAB^+) Calcd. for $C_{38}H_{44}O_{14}Na$: (M+Na) 747.2629, found; 747.2738.

^{1}H NMR

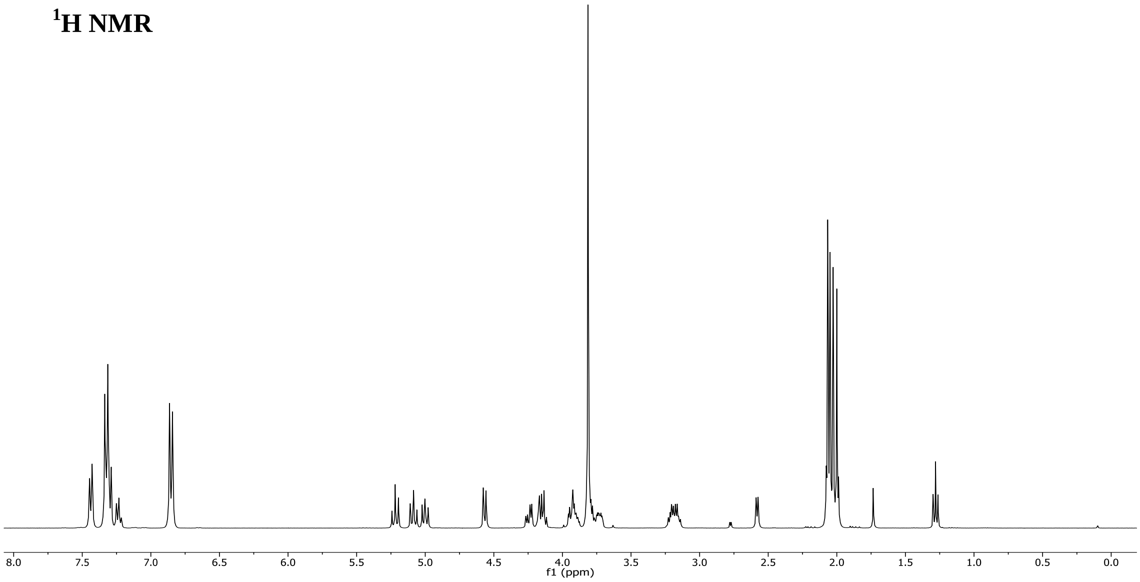

^{13}C NMR

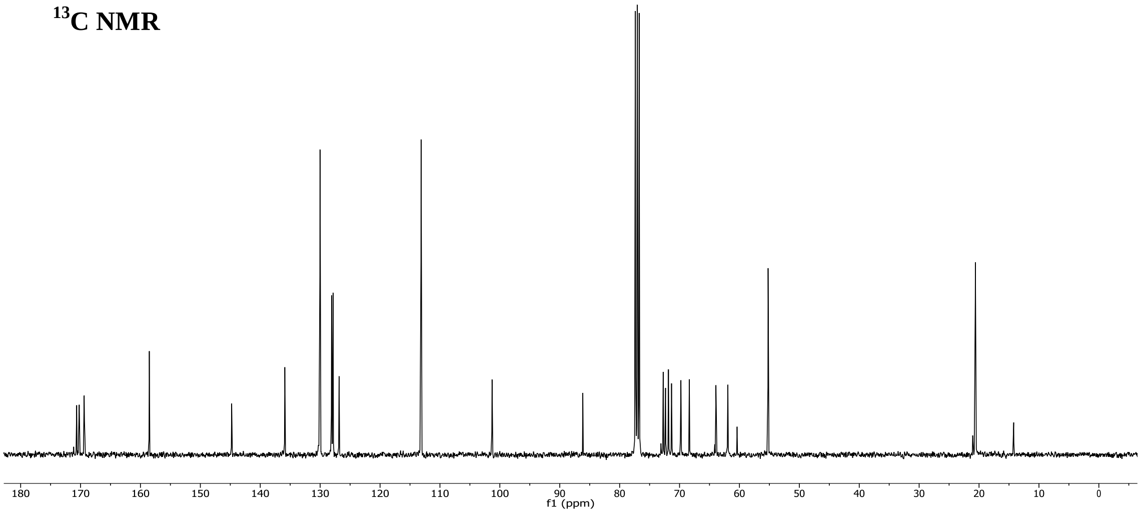

2-(*S*)-1-(4,4'-dimethoxytrytiloxy)-3-(2,3,4,6-tetra-*O*-acetyl-β-D-glucopyranosyloxy)propyl (2-cyanoethyl) (*N*,*N'*-diisopropyl) phosphoramidite (12)

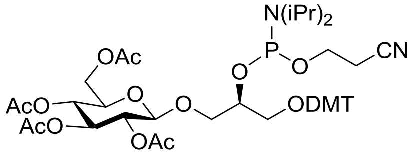

12

DIPEA (117 µL, 0.66 mmol) and 2-cyanoethyl-N,N'-diisopropylamino-chlorophosphoramidite (55 µL, 0.245 mmoL) were added to a solution of compound **11** (118 mg, 0.16 mmol) in anhydrous CH_2Cl_2 (5 mL) at room temperature under an argon atmosphere. After 20 min no starting material was observed. Solvent was then removed and the crude was purified by silica gel column chromatography by using Hex/EtOAc (3:1-1:1 with 5% of NEt_3) to give compound **12** (128 mg, 85%) as a white foam. ^{1}H NMR (400 MHz, $CDCl_3$) δ ppm : ^{1}H NMR (400 MHz, $CDCl_3$): δ = 7.44 (t, *J* = 6.2 Hz, 2H), 7.31 (dt, *J* = 14.2, 7.3 Hz, 6H), 7.26 – 7.20 (m, 1H), 6.84 (t, *J* = 7.1 Hz, 4H), 5.25 – 5.02 (m, 2H), 4.95 (q, 1H), 4.70 – 4.52 (m, 1H), 4.28 (td, *J* = 11.6, 4.7 Hz, 1H), 4.20 – 3.91 (m, 3H), 3.91 – 3.77 (m, 7H), 3.77 – 3.44 (m, 5H), 3.25 (m, 2H), 3.15 – 3.00 (m, 1H), 2.64 (q, 1H), 2.45 (q, 1H), 2.09 – 1.98 (m, 9H), 1.92 (m, 2H), 1.27 - 1.19 (m, 12H), 1.04 (d, *J* = 7.0 Hz, 3H); ^{13}C NMR (101 MHz, $CDCl_3$): δ = ^{13}C NMR (101 MHz, $CDCl_3$) δ 170.6, 170.2, 169.4, 169.1, 158.5, 144.8, 136.0, 130.1, 130.00, 128.2, 128.1, 127.8, 126.8, 118.05, 117.6, 113.1, 100.8, 100. 6, 86.1, 73.0, 72.9, 72.3, 72.2, 72.0, 71.8, 71.7, 71.3, 71.2, 70.1, 69.9, 68.4, 63.5, 63.4, 61.9, 58.45, 58.3, 55.2, 43.2, 24.7, 24.6, 24.5, 20.7, 20.6, 20.4,

20.2; ^{31}P NMR (CDCl3, 161.9 MHz): δ ppm 149.0 (d, *J*= 8 Hz). HRMS (FAB$^+$) Calcd. for $C_{47}H_{61}N_2KO_{15}P$: (M+K) 963.3447, found; 963.3789.

^1H NMR

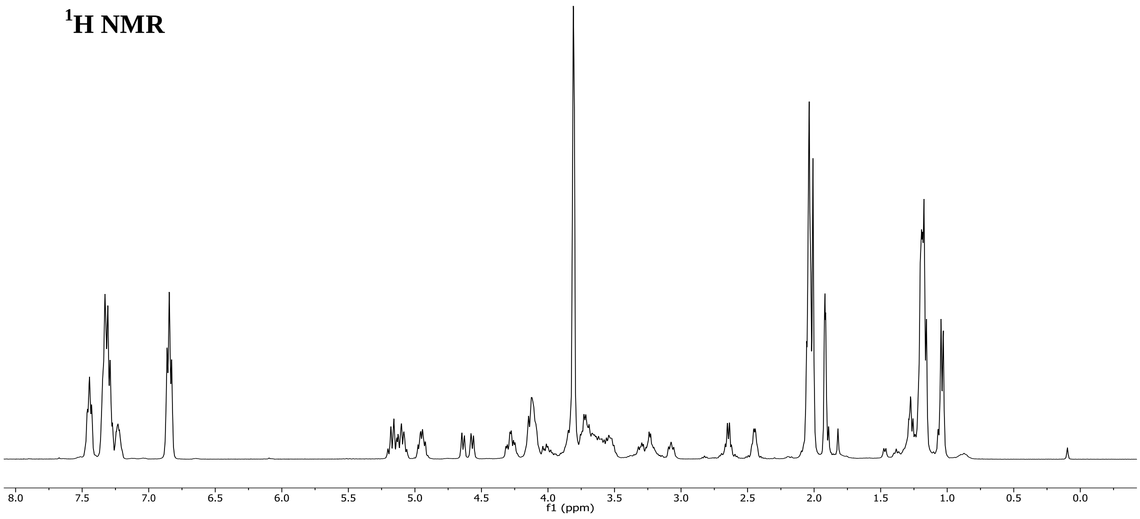

^{13}C NMR

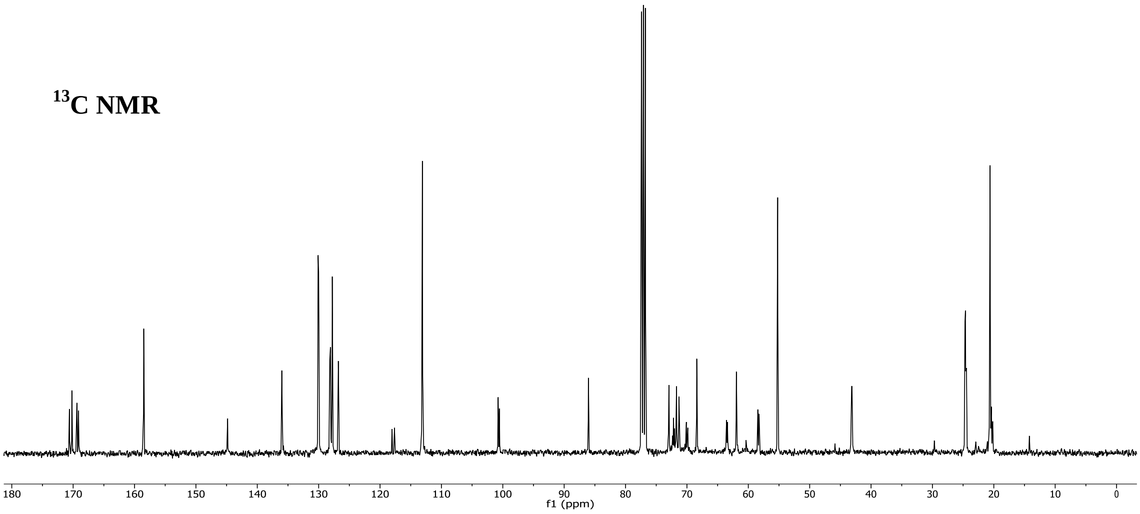

^{31}P NMR

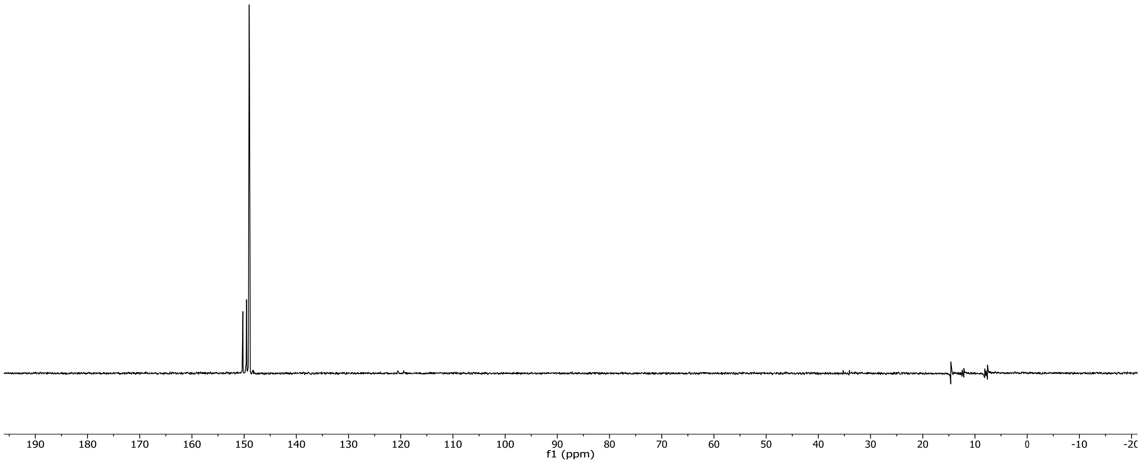

(S)-2-(2,3,4-tri-*O*-acetyl-6-deoxy-β-D-glucopyranosyl)-4-*O*-benzyl-1-*O*-tert-butyldimethylsilyl-butanetriol 15

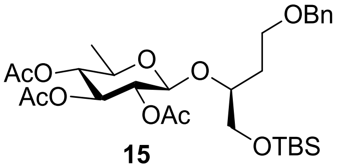

15

To a solution of 2,3,4-tetra-*O*-acetyl-6-deoxy-α,β-D-glucopyranosyl trichloroacetimidate **13**[1] (1.4 g, 3.22 mmol) and (*S*)-(+)-4-Benzyloxy-1[tert-butyldimethylsilanyloxy]- butan-2-ol **14**[2] (0.833 mg, 2.68 mmol) in anhydrous CH_2Cl_2 (40 ml) at -10ºC, $BF_3.OEt_2$ (42 μl, 0.32 mmol) was then added. The reaction was then stirred for 15 min and NEt_3 (0.2 ml) was then added. The solvent were removed and the crude was purified by flash column chromatography (Hex/EtOAc, from 6:1 to 2:1) to afford **15** (980 mg, 63%); ^{1}H NMR (500 MHz, $CDCl_3$) δ (ppm): 7.34-7.31 (m, 5H, Ph), 5.12 (t, *J* =9.6 Hz, 1H, H_3), 4.93 (dd, *J* = 8.0, 9.6 Hz, 1H, H_2), 4.82 (t, *J* = 9.60 Hz, 1H, H_4), 4.77 (d, *J* =8.0 Hz, 1H, H_1), 3.94-3.93 (m, 1H, -CH-), 3.61-3.47 (m, 5H, H_5, -OCH*H*), 2.03, 2.02, 1.99 (3s, 12H, 3x-$OCOCH_3$), 1.83-1.67 (m, 2H, -CH-), 1.19 (d, 3H, J = 6.0 Hz, CH_3), 0.90 (s, 9H, -$C(CH_3)_3$), 0.05 (s, 6H, $Si(CH_3)_2$); ^{13}C NMR (75 MHz, $CDCl_3$): δ = 170.2, 169.5, 169.3 (CO), 138.6, 128.1, 127.4, 127.3 (Carom), 99.8 (C_1), 76.5, 73.4, 72.9, 72.8, 71.9, 69.6, 66.5, 66.1, 32.0, 25.7, 20.6, 20.5, 18.1, 17.2-5.59. HRMS (FAB^+) Calcd. for $C_{29}H_{46}NaO_{10}Si$ (M+Na): 605.2758, found; 605.2767.

^{1}H NMR

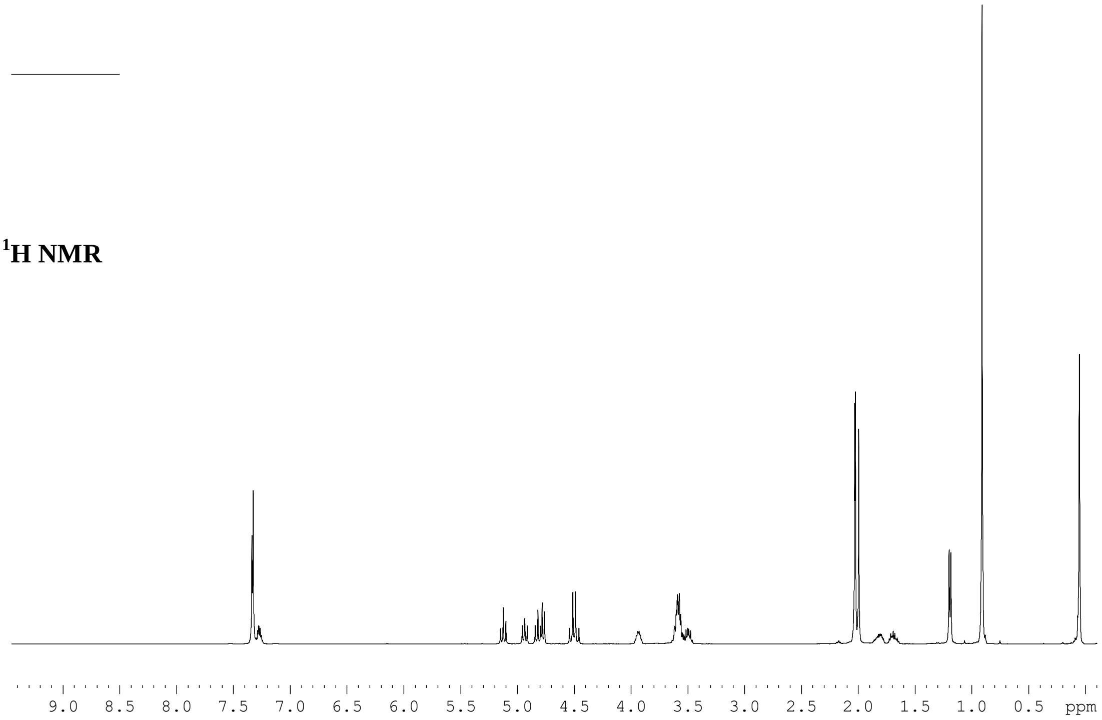

^{13}C NMR

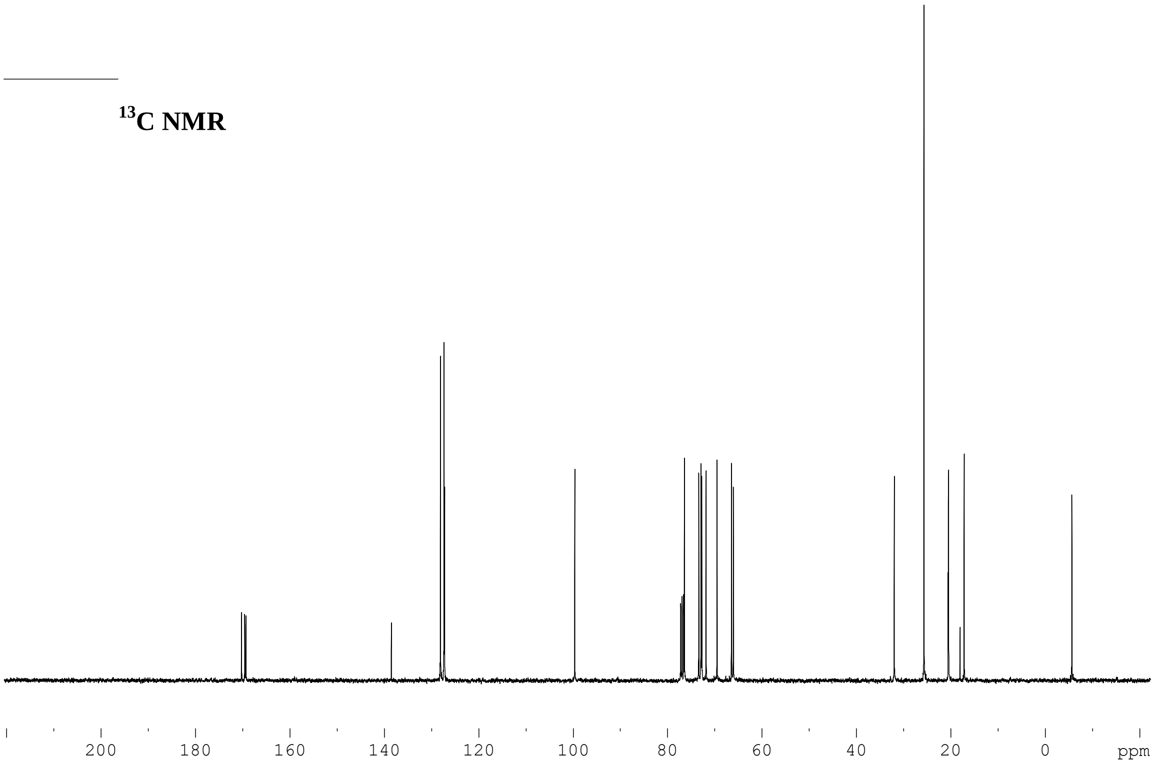

(S)-2-(2,3,4-tri-*O*-acetyl-6-deoxy-β-D-glucopyranosyl)-1-*O*-tert-butyldimethylsilyl-4-(4,4'-dimethoxytrityloxy)-butanetriol 16

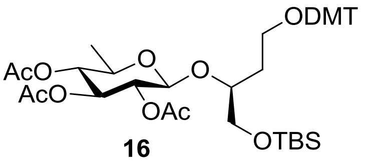

16

A solution of compound **15** (460 mg, 0.72 mmol) in ethyl acetate (10 mL) and $Pd(OH)_2$ in catalytic amount was stirred under an atmosphere of hydrogen for 1 h. The mixture was filtered off over celite and solvents were removed. TLC showed the deprotected compound was pure and it was used for the next step without any further purification. To a solution of the deprotected compound (240 mg, 0.48 mmol) in dry pyridine-CH_2Cl_2 (1:1, 8 ml) was reacted following the general procedure for primary hydroxyl protection. The crude was purified by silica gel column chromathography (Hex/EtOAc, from 3:1 to 1:1) to give **16** (325 mg, 84%). ^{1}H NMR (500 MHz, $CDCl_3$) δ (ppm): 7.48-7.47 (d, 2H, H_{arom}), 7.37-7.19 (m, 7H, H_{arom}), 6.86-6.84 (d, 4H, H_{arom}),5.16 (t, *J* =9.5Hz, 1H, H_3), 4.90 (dd, *J* =7.8 and 9.20 Hz, 1H, H_2), 4.81 (t, *J* =9.60 Hz, 1H, H_4), 4.78 (d, *J* =8.0 Hz, 1H, $H_{1,}$), 3.97-3.96 (m, 1H, -CH-), 3.79 (s, 9H, MeO), 3.62-3.60 (m, 2H, -CH-), 3.48-3.45 (m, 1H, -CH-), 3.22 (t, 2H, H_5, -CH-), 2.05, 2.03, 2.02 (3s, 12H, 3x-$OCOCH_3$), 1.85-1.79 (m, 2H, CH_2), 1.15 (d, 3H, J = 6.0 Hz, CH_3), 0.94 (s, 9H, $C(CH_3)_3$), -0.08 (s, 6H, 2x-$Si(CH_3)_2$); ^{13}C NMR (75 MHz, $CDCl_3$): δ = 170.4, 169.7, 169.4 (CO), 158.6, 158.4 147.5, 145.5, 139.6, 136.7, 130.1, 130.0, 129.2, 128.2, 127.8, 127.7, 127.0, 126.0, 113.1, 113.0, 99.7, 85.9, 81.4, 73.6, 73.2, 72.1, 69.7, 66.1, 60.1, 55.2, 55.1, 32.7, 25.9, 20.8, 20.7, 20.6, 18.3, 17.4, -5.4. HRMS (FAB$^+$) Calcd. for $C_{43}H_{58}NaO_{12}Si$ (M+Na): 817.3595, found; 817.3582.

^{1}H NMR

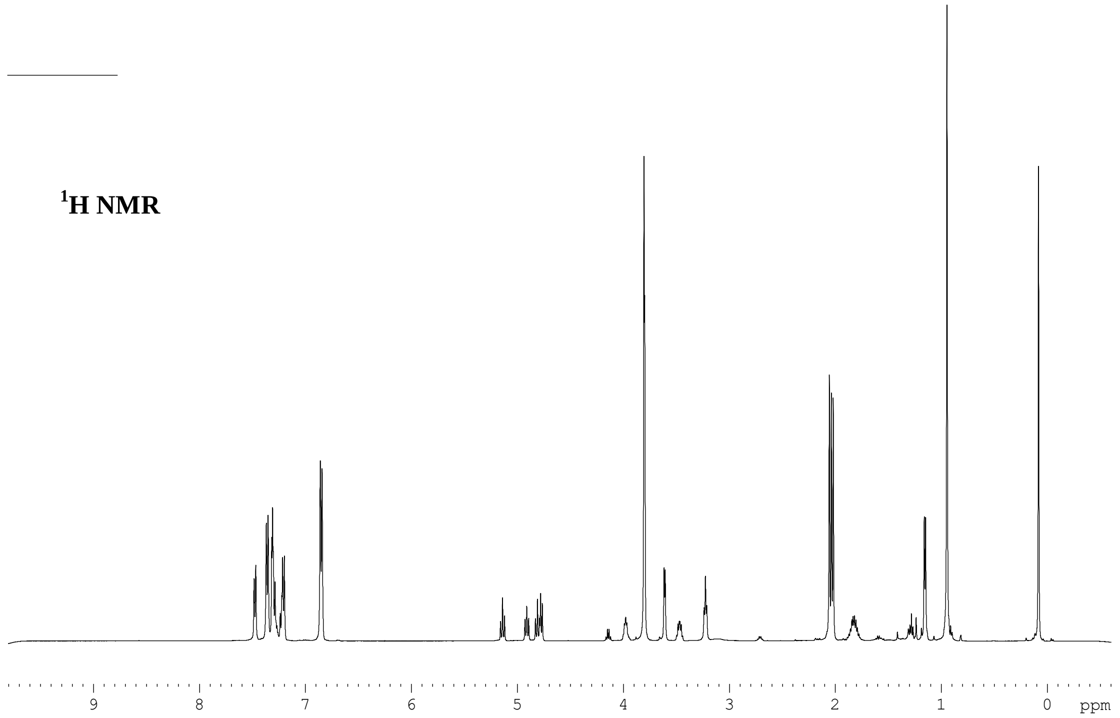

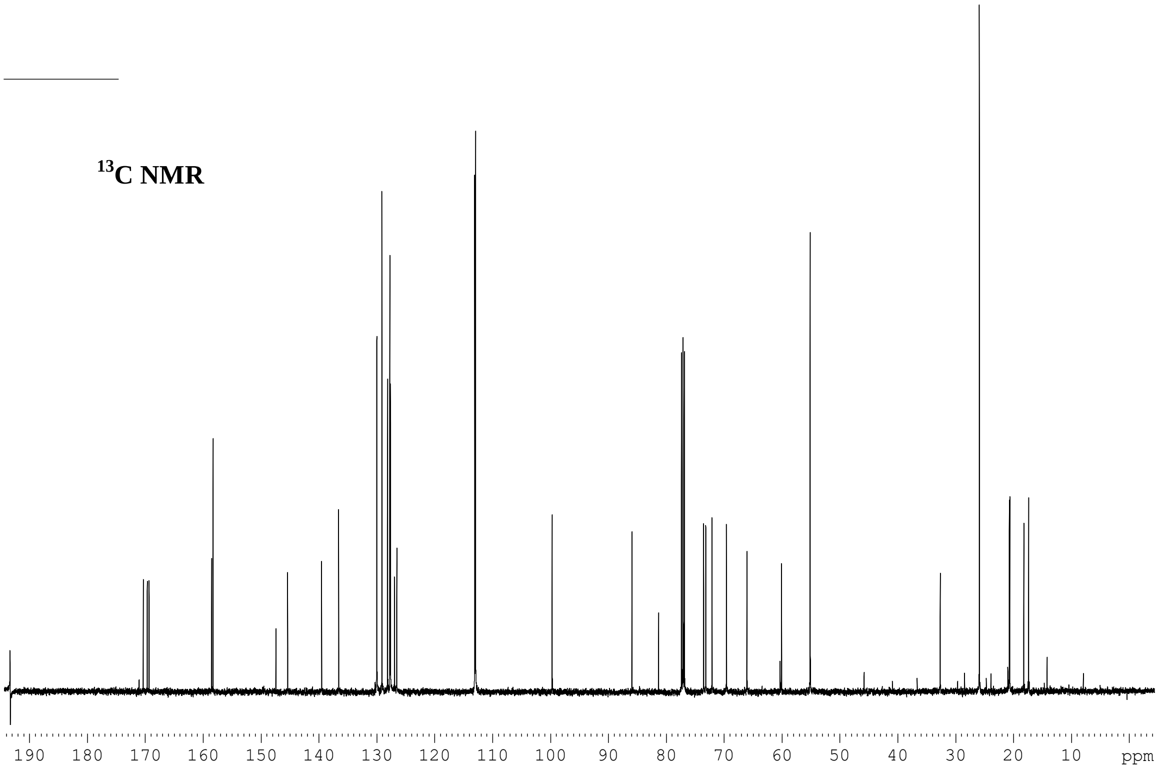

(S)-2-(2,3,4-tri-O-acetyl-6-deoxy-β-D-glucopyranoside)-4-(4,4'-dimethoxytrityloxy)-butanetriol 17

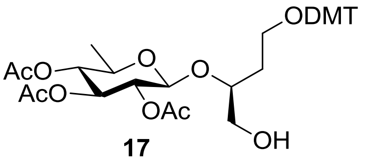

To a solution of compound **16** (110 mg, 0.138 mmol) in methanol (10 mL) was added tetrabutyl ammonium fluoride (TBAF, 145 mg, 0.55 mmol) and the mixture was stirred overnight at room temperature. Solvent was then removed and the mixture was resuspended in ethyl acetate and extracted with saturated NH_4Cl (2x 50 ml). The crude was purified by silica gel column chromathography (Hex/EtOAc, from 3:1 to 1:2) to give compound **17** (30 mg, 32%). ^{1}H NMR (300 MHz, $CDCl_3$) δ (ppm): 7.49 – 7.40 (m, 2H, H_{arom}), 7.37 – 7.17 (m, 7H, H_{arom}), 6.92 – 6.77 (m, 4H, H_{arom}), 5.15 (t, *J* =9.5Hz, 1H, H_3), 4.94 (dd, *J* =9.7 and 8.0 Hz, 1H, H_2), 4.81 (t, *J* =9.60 Hz, 1H, H_4), 4.59 (d, *J* =8.0 Hz, 1H, $H_{1,}$), 4.01-3.89 (m, 1H, -CH-), 3.83 (s, 6H, MeO), 3.69-3.50 (m, 2H, -CH-), 3.50-3.37 (m, 1H, -CH-), 3.23 (t, 2H, H_5, -CH-), 2.07, 2.04, 2.02 (3s, 9H, 3x-$OCOCH_3$), 1.97-1.72 (m, 2H, CH_2), 1.16 (d, 3H, J = 6.2 Hz, CH_3); ^{13}C NMR (75 MHz, $CDCl_3$): δ = 170.3, 169.7, 158.5, 145.1, 136.3, 136.3, 130.0, 128.1, 127.8, 126.8, 113.2, 113.1, 100.1, 86.3, 79.6, 76.6, 73.2, 72.9, 72.3, 69.9, 64.9, 59.9, 55.2, 32.4, 29.7, 22.7, 20.7, 20.7, 17.4; HRMS (FAB$^+$) Calcd. for $C_{37}H_{44}NaO_{12}$ (M+Na): 703.2730 found; 703.2742.

^{1}H NMR

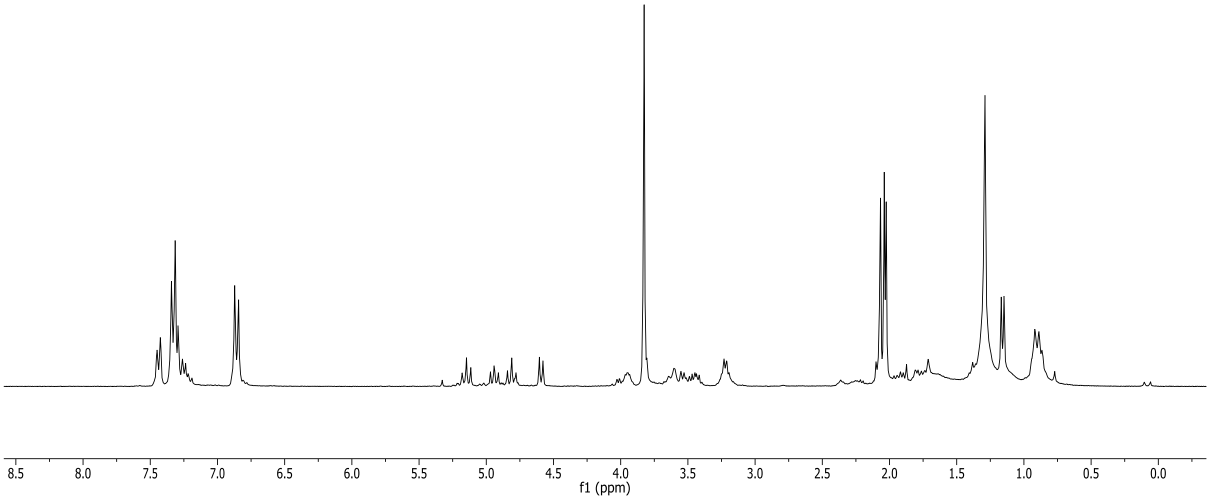

^{13}C NMR

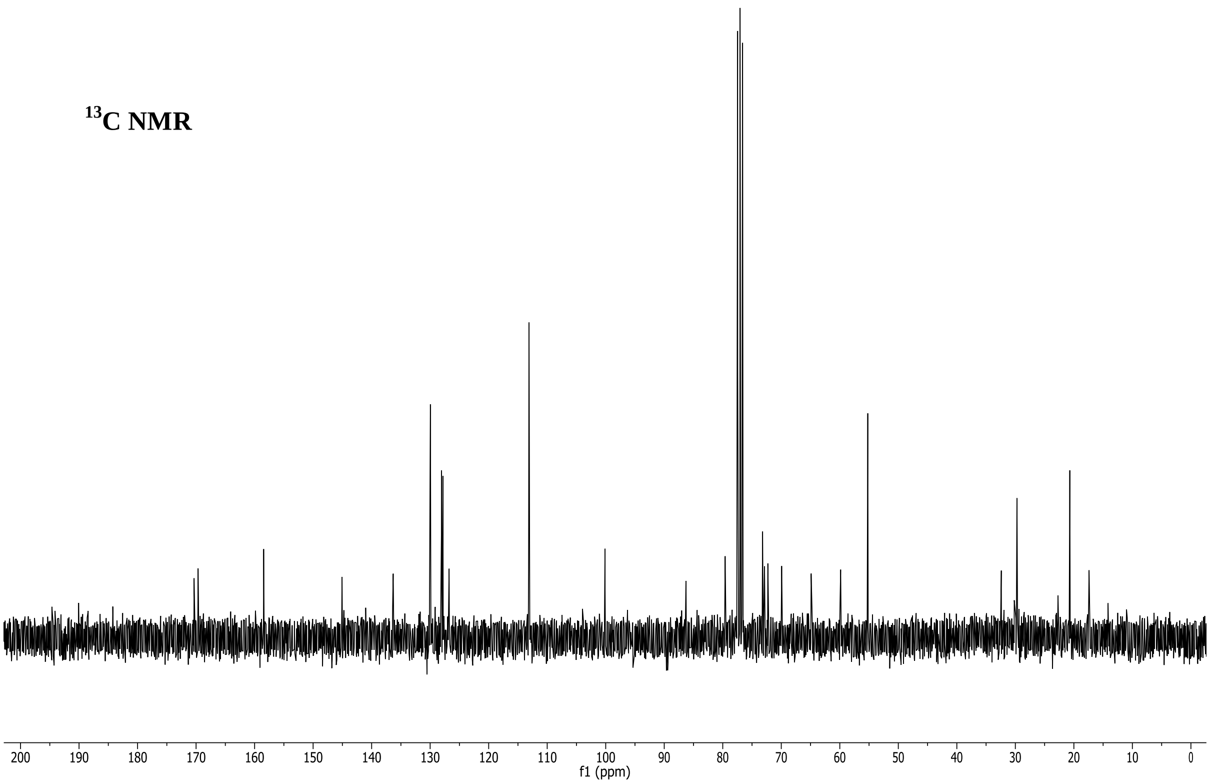

(S)-2-(2,3,4-tri-O-acetyl-6-deoxy-β-D-glucopyranoside)-4-(4,4'-dimethoxytrityloxy)-butyl (2-cyanoethyl) (*N,N'*-diisopropyl) phosphoramidite 18

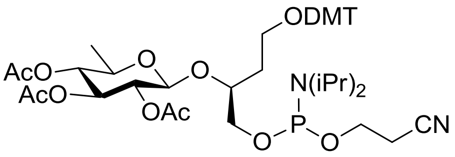

18

DIPEA (314 μL, 0.176 mmol) and 2-cyanoethyl-N,N'-diisopropylamino-chlorophosphoramidite (15 μL, 0.066 mmoL) were added to a solution of compound **17** (30 mg, 0.044 mmol) in anhydrous CH_2Cl_2 (10 mL) at room temperature under an argon atmosphere. After 45 min no starting material was observed. Solvent was then removed and the crude was purified by silica gel column chromatography by using Hex/EtOAc/NEt_3 (3:1:0.1) to give compound **18** (30 mg, 77%) as a white foam. ^{1}H NMR (500 MHz, $CDCl_3$) δ (ppm): 7.47 – 7.38 (m, 2H, H_{arom}), 7.35 – 7.16 (m, 7H, H_{arom}), 6.88 – 6.74 (m, 4H, H_{arom}), 5.12 (t, *J* = 9.6 Hz, 1H), 4.99 – 4.84 (m, 1H), 4.77 (t, *J* =9.60 Hz, 1H), 4.65 (d, *J* = 8.1 Hz, 1H), 4.03 – 3.97 (m, 1H), 3.80 (s, 6H, MeO), 3.70 – 3.56 (m, 3H), 3.55 – 3.41 (m, 2H), 3.22 – 3.18 (m, 2H), 2.74 – 2.54 (m, 2H), 2.34 (m, 1H), 2.04, 2.03, 2.00 (3s, 9H, 3x-$OCOCH_3$), 1.82 – 1.77 (m, 2H), 1.68 (m, 1H), 1.30 – 1.24 (m, 12H), 1.18 (d, *J* = 6.6 Hz, 3H, CH_3). ^{13}C NMR (126 MHz, $CDCl_3$): δ = ^{13}C NMR (126 MHz, $cdcl_3$) δ 173.51, 170.34, 169.67, 169.39, 158.33, 145.35, 136.58, 130.08, 130.02, 130.00, 129.97, 128.16, 127.66, 126.59, 113.08, 113.04, 112.97, 112.92, 99.87, 99.81, 85.99, 73.46, 73.43, 73.00, 72.94, 71.97, 69.70, 66.79, 60.01, 55.18, 43.05, 38.74, 34.00, 32.78, 31.92, 30.41, 29.69, 29.36, 28.91, 24.65, 24.63, 24.48, 23.79, 20.70, 20.65, 17.36; ^{31}P NMR (202 MHz, $CDCl_3$) δ (ppm): 148.6, 148.0; HRMS (FAB^+) Calcd. for $C_{46}H_{61}NaO_{13}P$ (M+Na): 903.3809, found; 903.3798.

^{1}H NMR

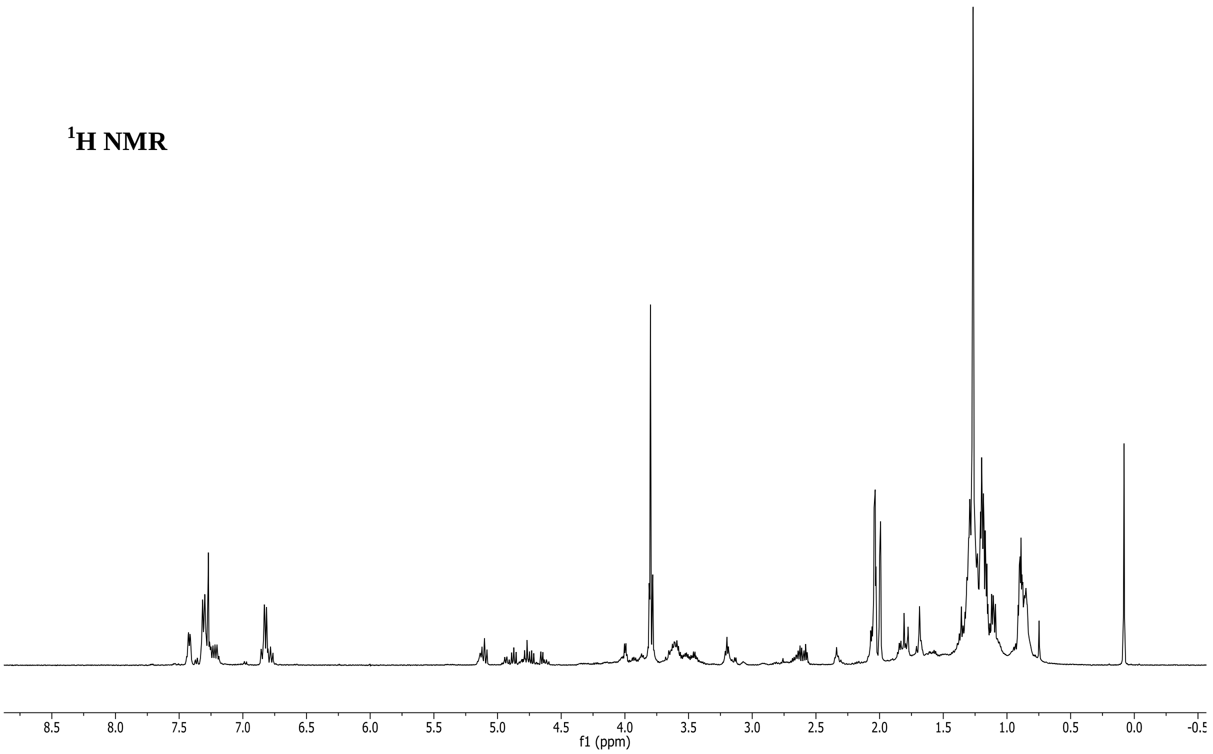

^{13}C NMR

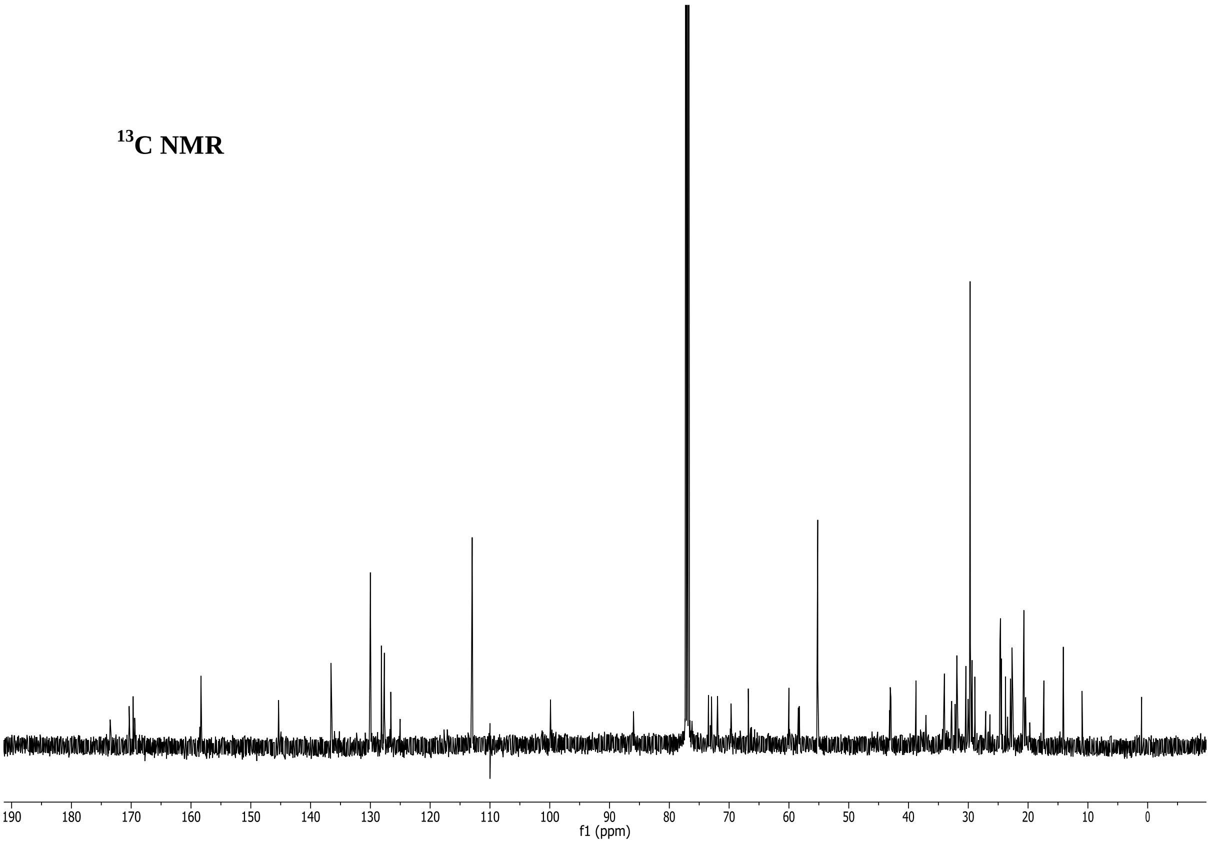

^{31}P NMR

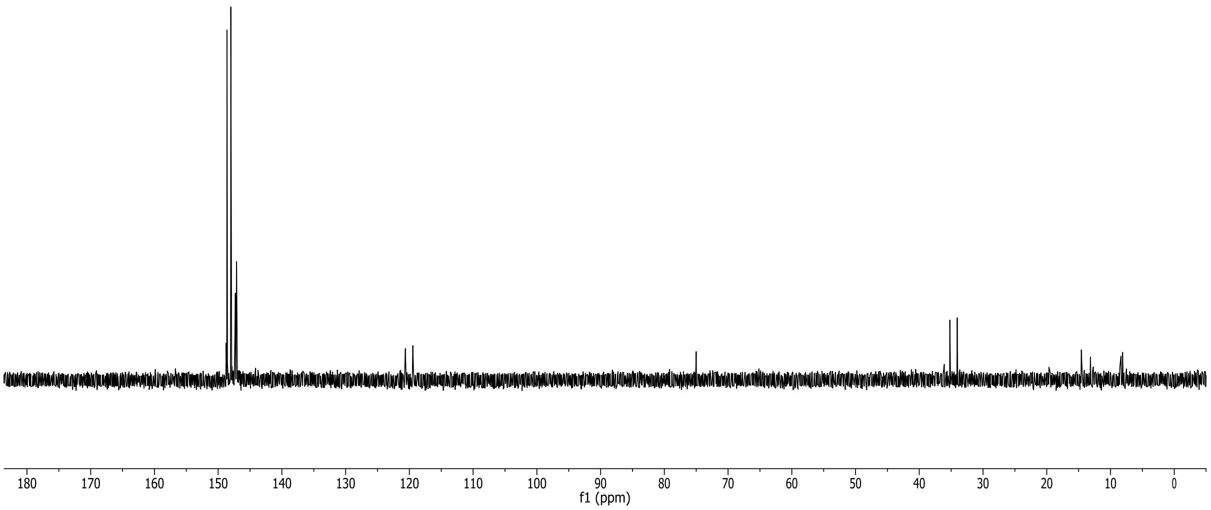

Synthesis of natural and modified oligonucleotide DNA strands

All natural and modified oligonucleotide DNA strands were synthesized by *Biomers* following standard β-cyanoethylphosphoramidite chemistry on 200 nmol or 1 umol scale and using the DMT-off procedure. Oligonucleotide supports were treated with 33% aqueous ammonia for 16 h at 55 ºC, then ammonia solutions were evaporated to dryness.

HPLC purification was carried out in a Waters Aliance 2690 RP-HPLC with a UV-Vis Photodiode Array (Waters) and using a Nucleosil 120 C18 (250 x 8 mm, 10 μm) column; 27 min linear gradient from 0 to 20% B (DMT off conditions); flow rate, 3 ml/ min; solution A was 5% acetonitrile in 0.1 M aqueous triethylammonium acetate (TEAA, pH 6.5) buffer and solution B was 70% acetonitrile in 0.1 M aqueous TEAA (pH 6.5).

5’-GATGAC-**glc(Me)**-GCTAG

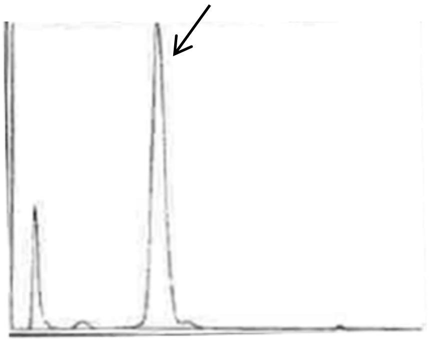

5’-CTAGC-**glc(Me)**-GTCATC

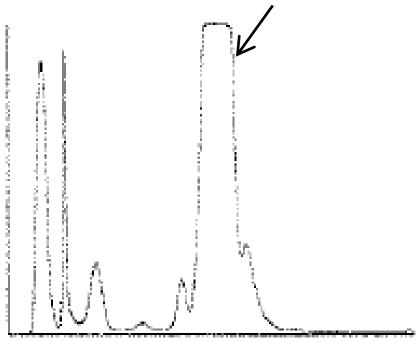

5’-GATGAC-**6dglcBT**-GCTAG

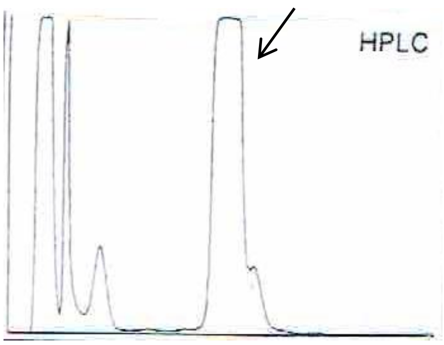

5’-CTAGC-**6dglcBT**-GTCATC

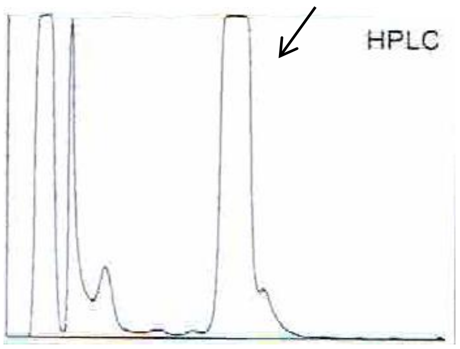

5’-GATGAC-**(R)glc**-GCTAG

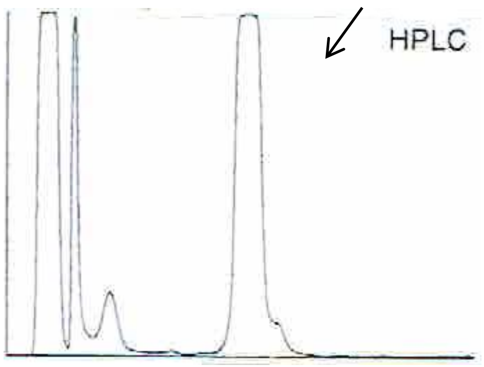

5’-CTAGC-**(R)glc**-GTCATC

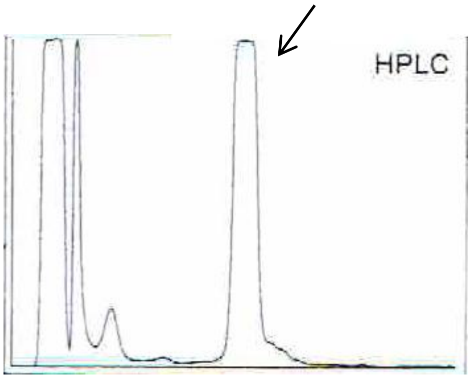

5’-GACTATA-**glc**-CCCTATAGTGAGTCGTATTA

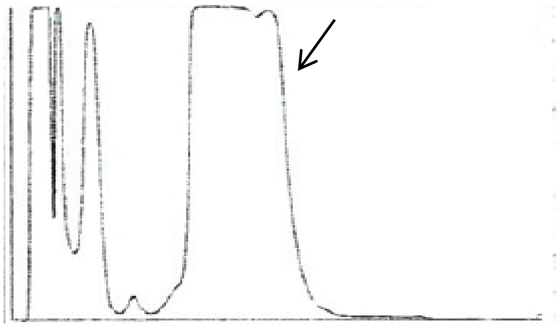

5’-GACTATA-**T***-CCCTATAGTGAGTCGTATTA

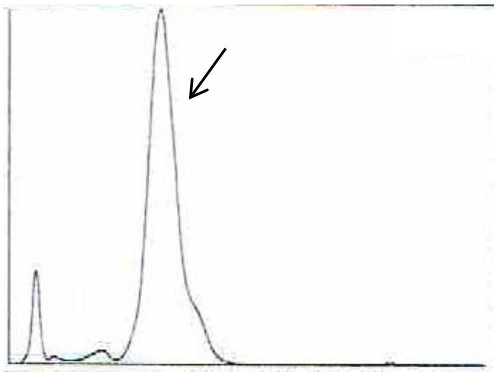

5’-GACTAT**C**-**glc**-CCCTATAGTGAGTCGTATTA

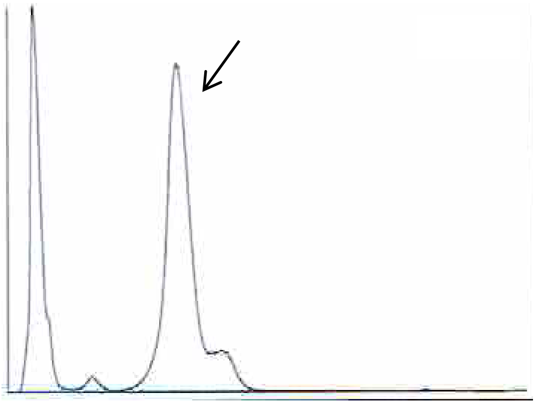

5’-GACTAT**C**-**6dglc**-CCCTATAGTGAGTCGTATTA

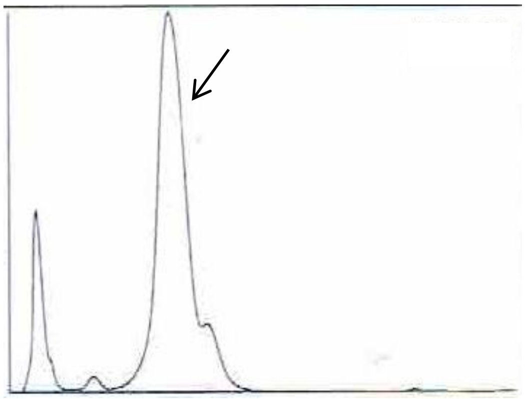

MALDI-TOF data of modified oligonucleotide DNA strands

Carbohydrate oligonucleotide conjugates	[M-H] calc.	[M-H] exp.
GATGAC-glc(Me)-GCTAG	3755	3755
CTAGC-glc(Me)-GTCATC	3666	3667
GATGAC-6dglcBT-GCTAG	3683	3681
CTAGC-6dglcBT-GTCATC	3594	3593
GATGAC-(R)glc-GCTAG	3698	3697
CTAGC-(R)glc-GTCATC	3609	3607
GACTATA-glc-CCCTATAGTGAGTCGTATTA	8591	8590
GACTATA-T*-CCCTATAGTGAGTCGTATTA	8551	8555
GACTATC-glc-CCCTATAGTGAGTCGTATTA	8454	8460
GACTATC-**6dglc**-CCCTATAGTGAGTCGTATTA	8249	8252

Synthesis of oligonucleotide DNA-GNA chimeric oligonucleotides

a. Synthesis of GNA Phosphoramidites.

The synthesis of DMT-phosphoramidite glycidol A and T followed the reported procedure described by Meggers et al.[3]

b. Synthesis and purification of DNA-GNA chimeric oligonucleotides. DNA oligonucleotides were synthesized on the 0.2 µmol scale on an Applied Biosystems 3400 synthesizer following standard conditions. Modified amidites were dissolved in anhydrous acetonitrile (0.1 M) and all oligonucleotides were synthesized in DMT-ON mode. Then the solid support treated twice with 0.1 M DBU/ACN for five minutes and washed with 1% Et_3N/ ACN. The solid support was transferred to a screw-cap glass vial and incubated at room temperature for four hours with 1.5 mL of NH_3 solution (33%).The ammonia solutions were concentrated to dryness and the product was desalted on NAP-10 (Sephadex G-25) columns eluted with water. All the oligonucleotides were purified by HPLC. Semipreparative column: X-bridge™ OST C_{18}(10x50 mm, 2.5 µm); 30 min linear gradient from 0 % to 70%, flow rate 2 mL/min; solution A was 5% ACN in 0.1 M aqueous TEAA and B 70% ACN in 0.1 M aqueous TEAA. The desired DMT-ON product corresponds to the main peak. The pure fractions were combined and evaporated to dryness. The residue that was obtained was treated with 200µL of 10% AcOH solution and incubated at room temperature for 5 min. The aqueous solution was neutralized with TEAA and, finally, the deprotected oligonucleotide was desalted in a NAP-10 column. All oligonucleotides were quantified by absorption at 260 nm and confirmed by MALDI mass spectrometry. Matrix-assisted laser desorption ionization time-of-flight (MALDI-TOF) mass spectra were recorded on a Voyager-DETMRP spectrometer (Applied Biosystems) in negative mode (2,4,6-

trihidroxyacetophenone matrix with ammonium citrate as an additive), (Servei d'Espectrometría de Masses, Universitat de Barcelona).

Representative purification HPLC chromatograms of DNA-GNA chimeric oligonucleotides

In bold letters, A and T GNA derivatives and 6dGlc:

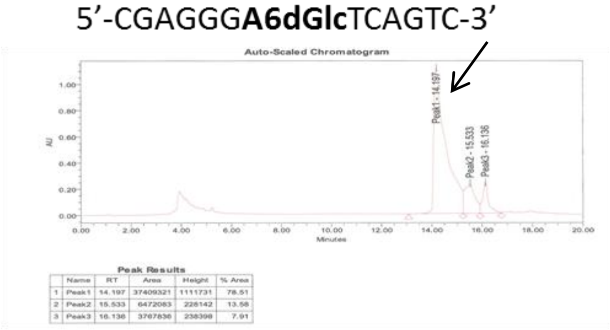

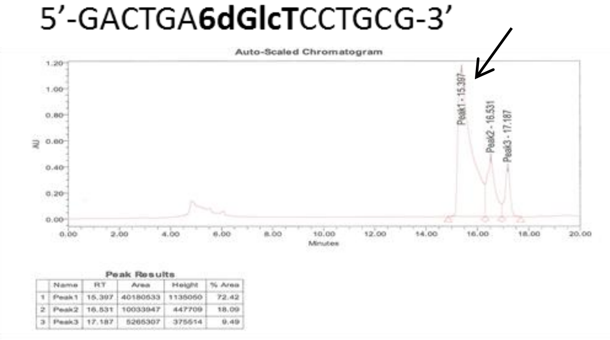

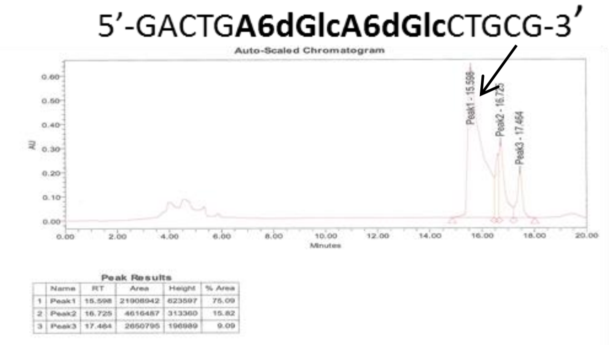

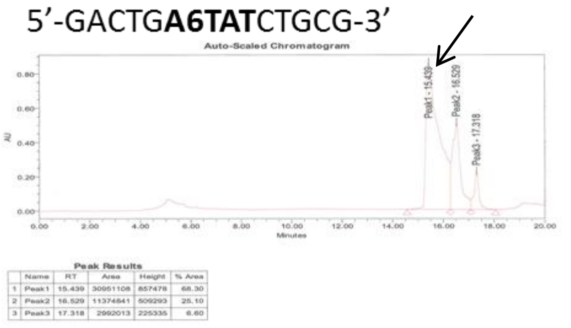

MALDI-TOF data of oligonucleotide DNA-GNA chimeric strands

In bold letters, A and T GNA derivatives and 6dGlc:

DNA-GNA chimeric oligonucleotide sequence	[M-H] calc	[M-H] found
5'-GACTGA**6dGlcA**CCTGCG-3'	4216.8	4218.2
5'-CGCAGG**6dGlcA**TCAGTC-3'	4216.8	4218.0
5'-GACTGA**TA**CCTGCG-3'	4178.4	4179.6
5'-CGCAGG**TA**TCAGTC-3'	4178.4	4179.9
5'-GACTGA**6dGlcT**CCTGCG-3'	4208.4	4204.5
5'-CGCAGG**6dGlcT**TCAGTC-3'	4208.4	4213.1
5'-GACTGA**6dGlc6dGlc**CCTGCG-3'	4246.4	4250.4
5'-CGCAGG**6dGlc6dGlc**TCAGTC-3'	4246.4	4250.6
5'-GACTG**A6dGlcA6dGlc**CTGCG-3'	4186.8	4190.2
3'-CGCAG**A6dGlcA6dGlc**CAGTC-3'	4155.8	4159.5
5'-GACTG**ATAT**CTGCG-3'	4110.8	4112.9
3'-CGCAG**ATAT**CAGTC-3'	4079.8	4083.2

Thermal denaturation methods.

- **DNA duplexes.**

UV-melting curves were measured on a Perkin–Elmer Lambda 750 UV/Vis spectrophotometer. Absorbance of duplexes in a 1:1 stoichiometric ratio were monitored at 260 nm and the heating rate was set to 1.0 ºC·min^{-1} from 10 to 80 ºC. The extinction coefficients of the natural oligonucleotide parts, ε_{nat}, was calculated using a *oligo calculator* (www.ambion.com). The total absorption coefficient was then calculated by simple addition: $\varepsilon = \varepsilon_{nat1} + \varepsilon_{nat2}$. The first derivative of the melting curves was obtained using *Origin 8.0* software. To avoid air water condensation samples were measured in a nitrogen atmosphere.

- **GNA duplexes.**

The absorbance versus temperature curves of duplexes were measured at 3.2 µM strand concentration in 10 mM sodium phosphate buffer at pH 7 implemented with 100 mM NaCl. Thermal UV experiments were performed in Teflon-stopped 1 cm path length quartz cells on a JACSO V-650 spectrophotometer equipped with thermoprogrammer. The samples were heated to 90ºC, allowed to slowly cool down to 20ºC, and then warmed during the denaturation experiments at a rate of 0.5 ºC/min from 10 to 85 ºC, monitoring absorbance at 260 nm. Melting temperatures (T_m) were determined by computerfit of the first derivative of absorbance with respect to 1/T.

NMR Spectroscopy.

Samples of all the conjugates and control duplexes were suspended in 500 µL of either D_2O or H_2O/D_2O 9:1 in phosphate buffer 10 mM , 150 mM NaCl, pH 7. NMR spectra were acquired in Bruker Avance spectrometers operating at 600 or 800 MHz and were processed with Topspin software. DQF-COSY, TOCSY, and NOESY experiments were recorded in D_2O. The NOESY spectra were acquired with mixing times of 150 and 300 ms, and the TOCSY spectra were recorded with standard MLEV-17 spin-lock sequence, and 80 ms mixing time. NOESY spectra in H_2O were acquired with 100 ms mixing time. In 2D experiments in H_2O, water suppression was achieved by including a WATERGATE module in the pulse sequence prior to acquisition. Two-dimensional experiments in D_2O were carried out at temperatures ranging from 5 to 25 °C, whereas spectra in H_2O were recorded at 5 °C to reduce the exchange with water. The spectral analysis program Sparky was used for semiautomatic assignment of the NOESY cross-peaks and quantitative evaluation of the NOE intensities. Distance constraints with their corresponding error bounds were incorporated into the AMBER potential energy by defining a flat-well potential term.

Structure Calculations.

Structures were calculated with the SANDER module of the molecular dynamics package AMBER. Starting models of the conjugate duplexes were built using the program SYBYL. The DNA moieties in the starting models were set to a standard B- canonical structure. These structures were taken as starting points for the AMBER refinement, which started with an annealing protocol in vacuo (using hexahydrated Na^+ counterions placed near the phosphates to neutralize the system). The resulting structures from *in vacuo* calculations were placed in the center of a water-box with around 4000 water molecules and 22 sodium counterions to obtain electroneutral systems. The structures were then refined including explicit solvent, periodic boundary conditions and the Particle-Mesh-Ewald method to evaluate long-range electrostatic interactions. Force field parameters for the carbohydrate moieties were taken from GLYCAM. The TIP3PBOX model was used to describe water molecules. The protocol for the constrained molecular dynamics refinement in solution consisted of an equilibration period of 160 ps using a standard equilibration process, followed by four independent 500 ps runs. Averaged structures were obtained by averaging the last 20 ps of individual trajectories and further energy minimization of the structure. Analysis of the representative structures as well as the MD trajectories was carried out with the program MOLMOL and the analysis tools of AMBER.

DFT quantum chemical methods.

All calculations were performed with the Amsterdam density functional program (ADF 2013.1) (http://www.scm.com/).[4] Calculations were initiated from the coordinates of the base pairs of interest in the PDB structures (2N9F and 2N9H) we obtained previously from NMR studies and molecular dynamics. The original guanine and thymine bases in the obtained structures were changed to adenine and cytosine, respectively, in order to evaluate the four possible pairs with the natural DNA bases. Bases, base-base pairs, sugar-base pairs and sugar-sugar pairs were optimized at the BLYP-D3(BJ)/TZ2P level of dispersion-corrected density functional theory (DFT) without any symmetrical constraints. Benchmarks have shown that dispersion-corrected DFT reproduces well high level ab initio results for weakly interacting systems (that is hydrogen bonding or stacking interactions).[5] Solvent effects in aqueous solution are described with the COnductor-like Screening MOdel (COSMO), which takes effectively into account solute–solvent interactions, cavitation, internal energy and entropy effects of the solvent and yields an estimate of the Gibbs free energies.[6]

DNA polymerase primer insertion and extension reactions

a. Materials and methods

T4 Polynucleotide Kinase was purchased from Fermentas (1 U of the enzyme transfers 1 nmol of γ-phosphate from ATP to 5'-OH DNA in 30 min at 37 °C). BIOTAQ™ DNA polymerase was purchased from Bioline (1 U is defined as the amount of enzyme that incorporates 10 nmol of total dNTPs into acid-insoluble form within 30 min at 72 °C). Therminator™, *Bst* 2.0 DNA polymerases were purchased from New England Biolabs (1 U is defined as the amount of enzyme that incorporates 10 nmol of total dNTPs into acid insoluble material in 30 min at 75 °C for Therminator and at 65 °C for *Bst* 2.0). Superscript III reverse transcriptase was purchased from Invitrogen (1 U is defined as the amount of enzyme that incorporates 1 nmol of dTTP into acid-precipitable material in 10 min at 37 °C using poly(A)·oligo$(T)_{25}$ as template/ primer). Klenow Fragment (exo$^-$) DNA polymerase from E.coli was purchased from Thermo Scientific (1 U is defined as the amount of enzyme that incorporates 10 nmol of deoxyribonucleotides into a polynucleotide fraction (adsorbed on DE-81) in 30 minutes at 37 °C, using poly(dA-dT)·poly(dA-dT) as a template/primer). Deoxynucleoside Triphosphate Set, PCR grade was purchased at Roche Life Science.

b. General procedure

Primer 5' termini was labelled using [γ-^{32}P]ATP and T4 polynucleotide kinase following the instructions of the manufacturer for T4 polynucleotide kinase. The labelled primer was annealed to the template in the corresponding polymerase buffer. Polymerase reactions were started by mixing equal volumes of solution A containing the DNA-enzyme complex and solution B containing dNTP substrates. Solution A was made by adding each polymerase diluted in annealing buffer to the annealed duplex DNA and incubating 2 min at 37 °C for Klenow Fragment, BIOTAQ™ and Therminator™ or 55 °C for *Bst* 2.0 and SuperScript III polymerase. Solution B contained 200 or 40 μM of dNTPs or 40 μM of dA/dC/dG/dT in polymerase buffer. The reaction mixture was incubated at 37 °C for Klenow Fragment, BIOTAQ™ and Therminator™ or 55°C for *Bst* 2.0 and SuperScript III polymerase, and terminated by adding one volume of stop buffer (95% formamide, 20 mM EDTA, 0.05% xylene cyanol and bromophenol blue). For single nucleotide insertions, the final concentrations used were: primer/template 5/6 μM; Klenow fragment (exo-) 0.2 units·μl^{-1}, SuperScript III 10 units·μl^{-1}, BIOTAQ™ 0.25 units·μl^{-1}, Therminator™ 0.1 units·μl^{-1} and *Bst* 2.0 0.4 units·μl^{-1}; and 20 μM of dA/dC/dG/dT. For multiple nucleotide insertion experiments the final concentrations used were: primer/template 5/6 μM, Klenow fragment (exo-) 0.2 units·μl^{-1}, SuperScript III 10 units·μl^{-1}, BIOTAQ™ 0.25 units·μl^{-1}, Therminator™ 0.1 units·μl^{-1} and *Bst* 2.0 0.4 units·μl^{-1}; dNTP 20 μM or 100 μM, as indicated. The reactions were incubated 15 or 60 min and the products of the reactions were visualized by running quenched reaction samples on a 20% denaturing polyacrylamide gel.

Positions of the oligodeoxynucleotides were located by high efficiency storage phosphor screens and scanned into a Perkin-Elmer Cyclone Plus Phosphorimager.

Steady-state kinetics. Steady-state kinetics for standing-start single nucleotide insertions were carried out as described.[7] The conditions used were the same as for the qualitative insertion and extension studies. The final DNA (duplex) concentration was 5 μM. Amount of polymerase used (3.35-134 nM), nucleotide concentrations (0.1μM - 2mM) and reaction times (0.5-90 min) were adjusted to give extents of reaction of 20% or less. For each insertion the primer-template was in excess over enzyme by 37 to 1500-fold in all cases. Extents of reaction were determined by running quenched reaction samples on a 15% denaturing polyacrylamide gel. Relative velocities were calculated as extent of reaction divided by reaction time and normalized to the lowest enzyme concentration used (3.35 nM) and to the highest primer-template concentration (5 μM).

Figure S1: Melting curves for carbohydrate oligonucleotide conjugates **7** and **14** in DNA duplex.

5'-d(GATGAC**X**GCTAG)
3'-d(CTACTG**Y**CGATC)

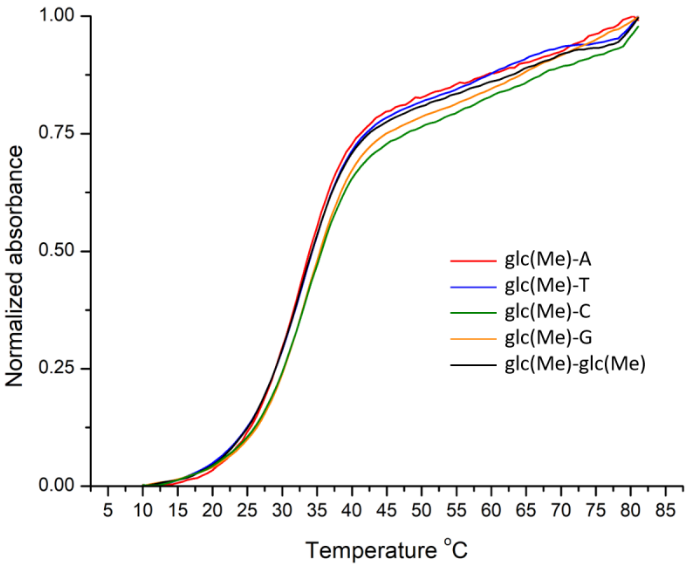

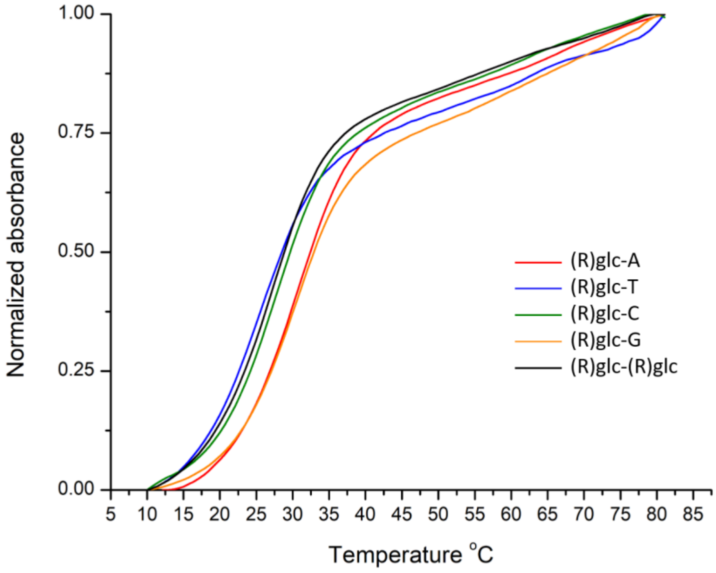

Figure S2: Melting curves for GNA-DNA chimeric double helices

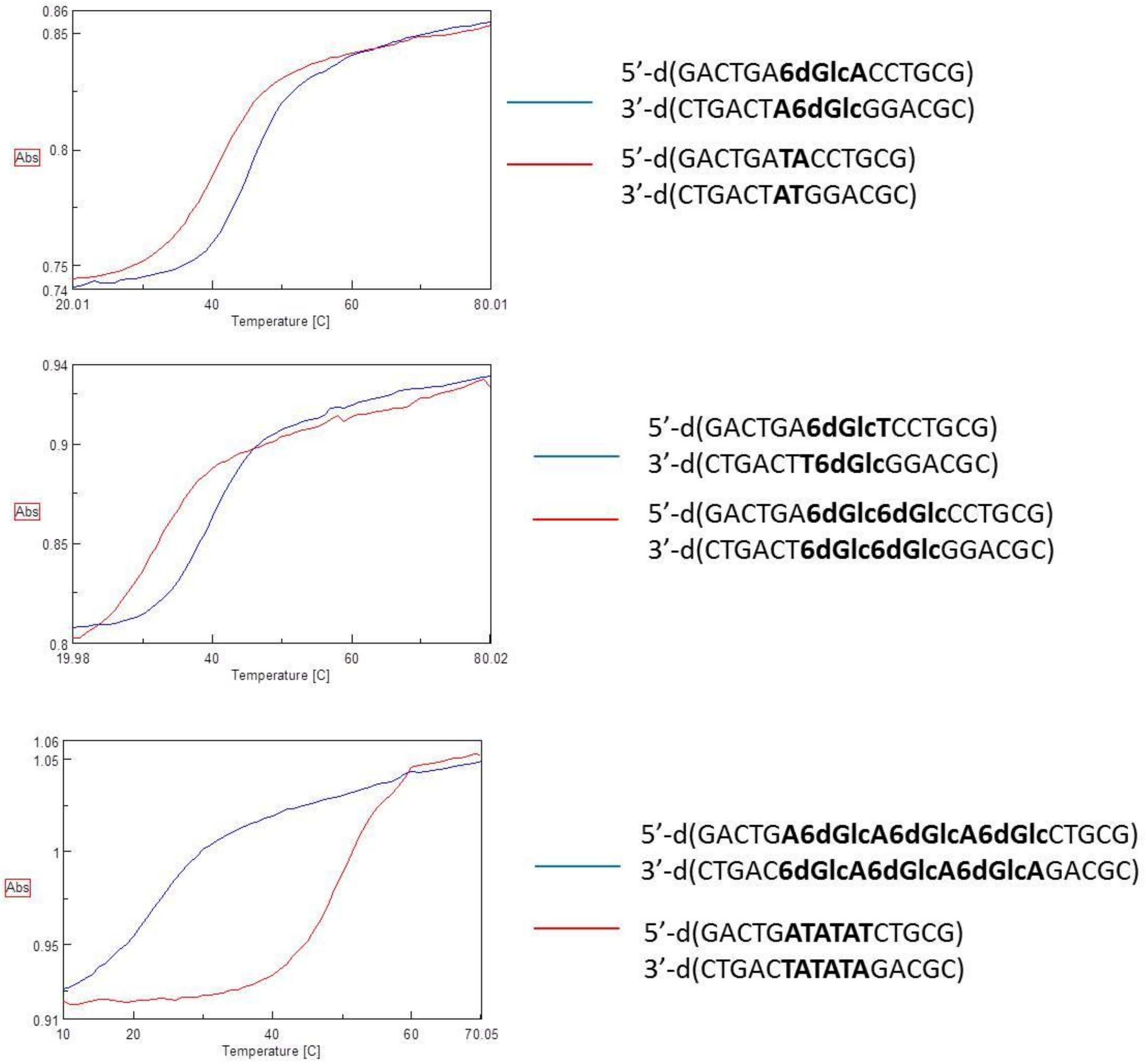

Figure S3. Imino región of the NMR spectra of **helix glc(Me)-G** and **helix glc(Me)-T**

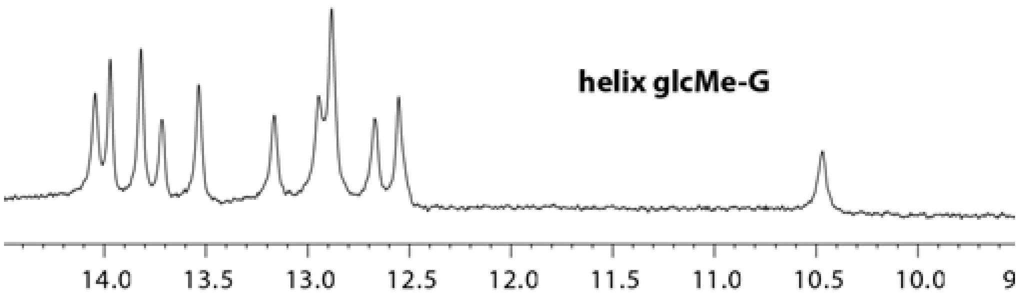

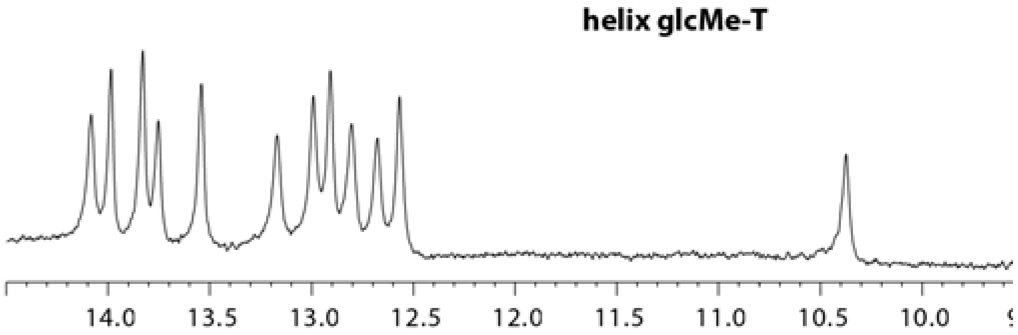

Figure S4. More significant changes in proton chemical shifts along the sequence for **helix glc(Me)-G** and **helix glc(Me)-T** with respect to double helices containing all natural base pairs.

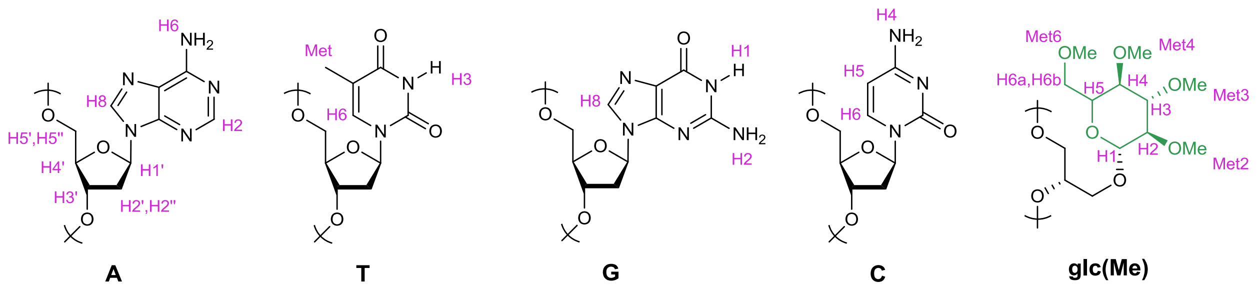

Helix glc(Me)-G 5'-C[1]TAGC**G**GTCATC[12]
3'-G[24]ATCG-**glc(Me)**-CAGTAG[13]

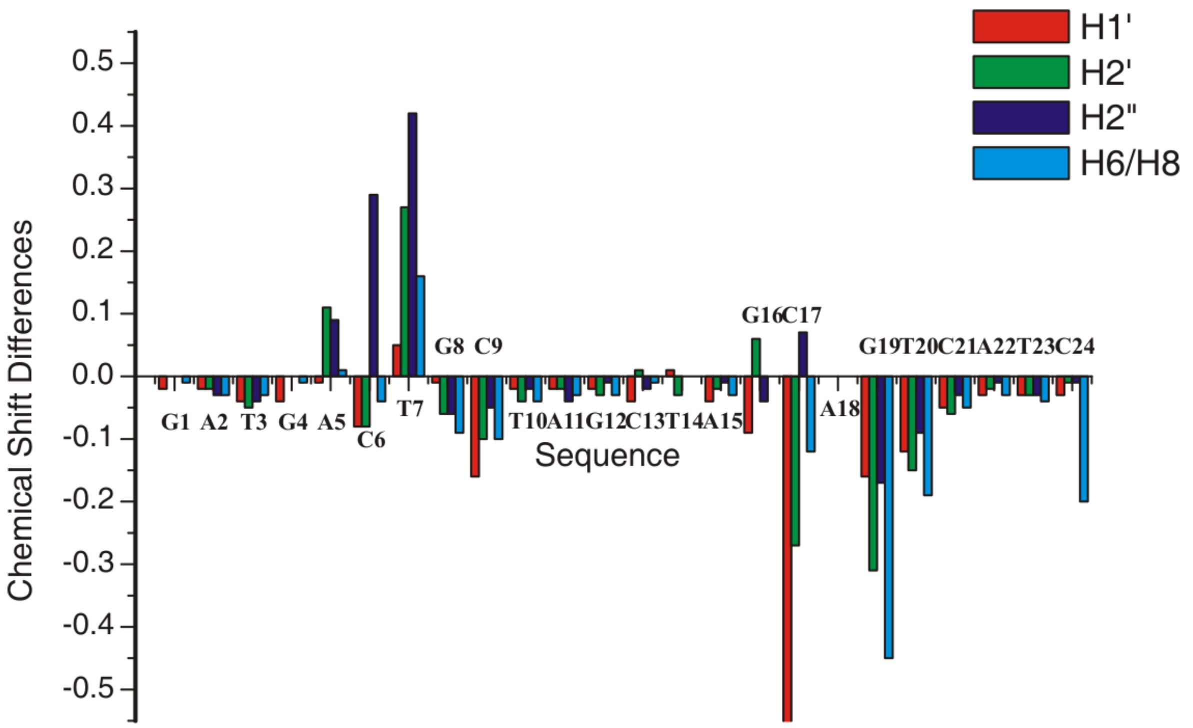

Helix glc(Me)-T 5'-G[1]ATGAC**T**GCTAG[12]
3'-C[24]TACTG-**glc(Me)**-CGATC[13]

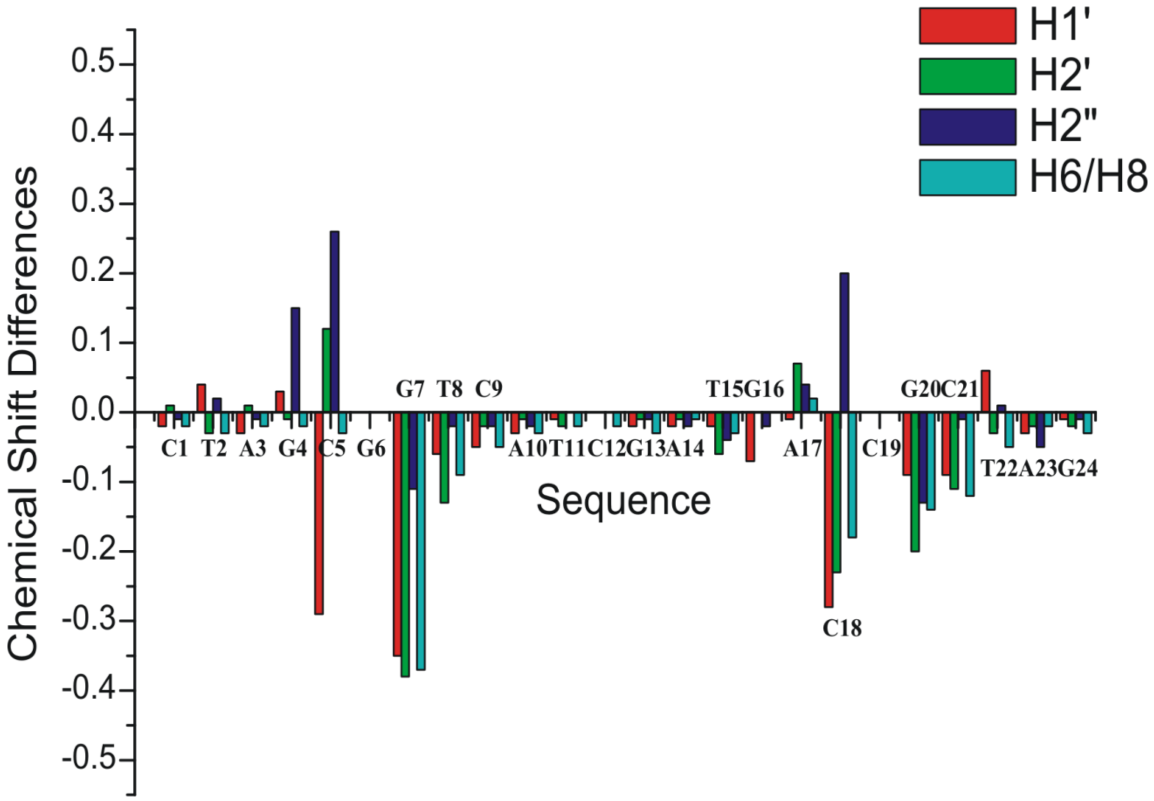

Figure S5.- Regions of NOESY spectra of **helix glc(Me)-T** in D_2O (mixing time 250ms, T= º5C). Sequential assignment pathways are shown in the H1´-base region. DNA carbohydrate contacts are labeled in red.

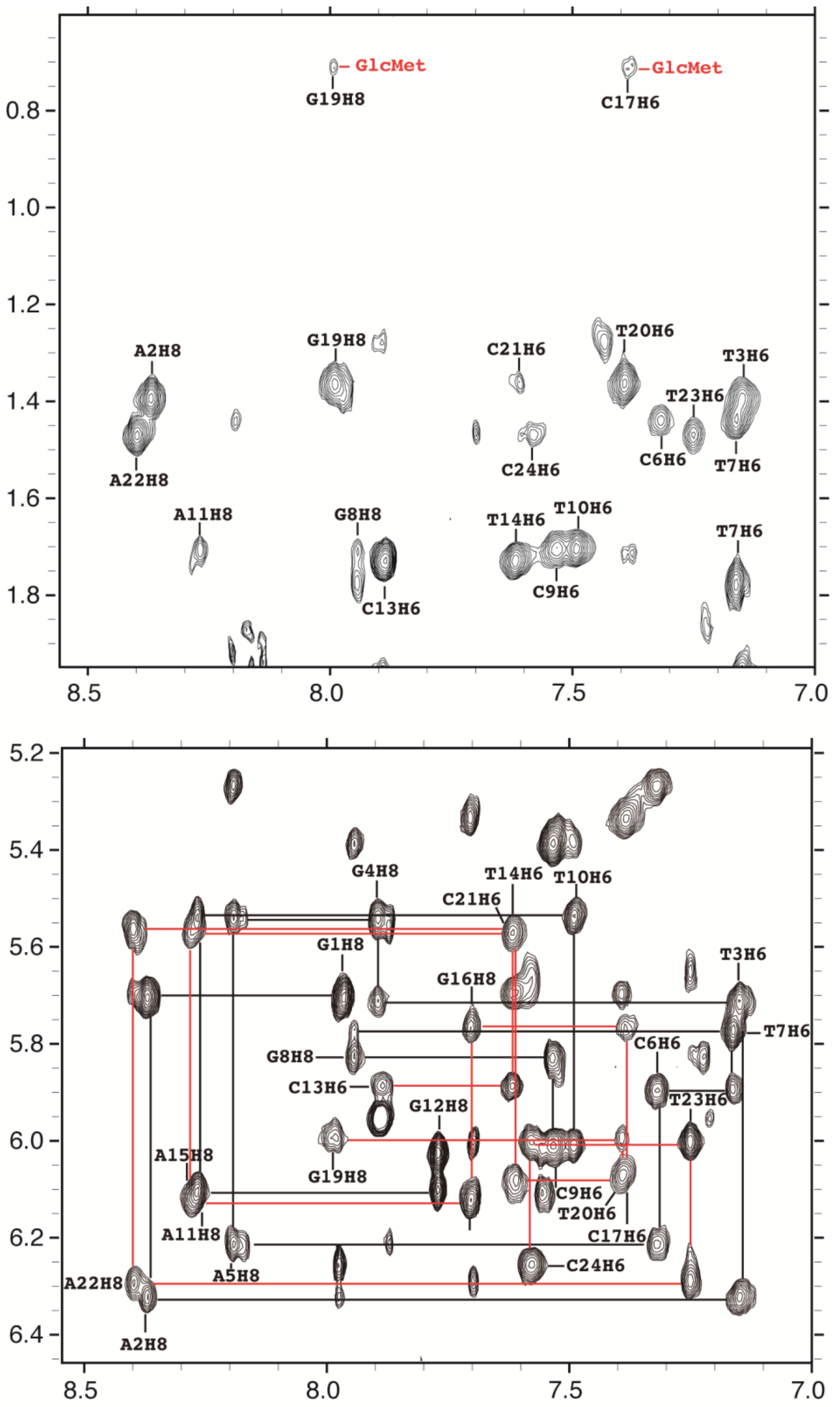

Figure S6. Solution structure of **helix glc(Me)-T.** a) Stereoscopic view of the ensemble of the 10 refined structures, b) Stereoscopic view of a representative structure. Color code: modified strand in green; complementary strand in blue; carbohydrate and linker in magenta; hydrogen atoms are shown in grey. c) Two views showing details of the carbohydrate moiety and the surrounding base-pairs. d) Detail of the interaction between carbohydrate and the opposite thymine.

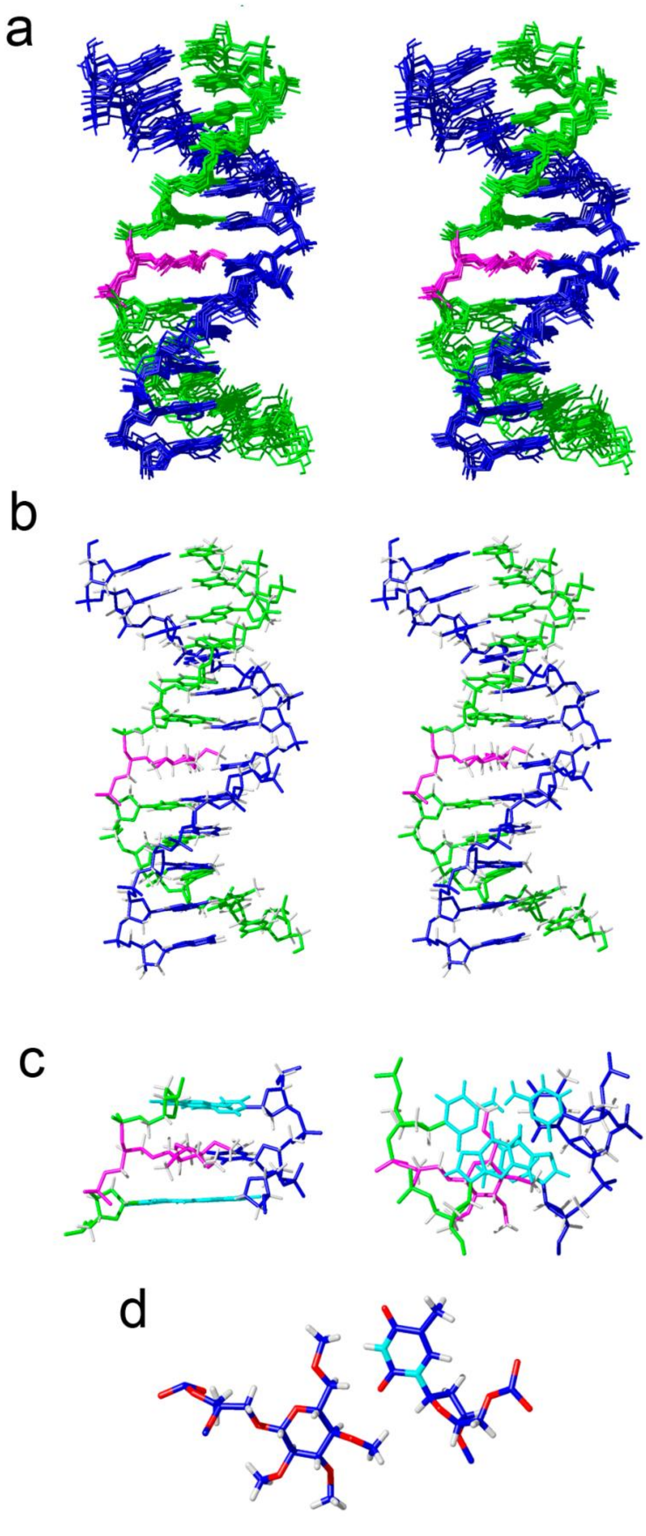

Figure S7. Solution structure of helix **glc(Me)-G.** a) Stereoscopic view of the ensemble of the 10 refined structures, b) Stereoscopic view of a representative structure. Color code: modified strand in green; complementary strand in blue; carbohydrate and linker in magenta; hydrogen atoms are shown in grey. c) Two views showing details of the carbohydrate moiety and the surrounding base-pairs. d) Detail of the interaction between carbohydrate and the opposite guanine.

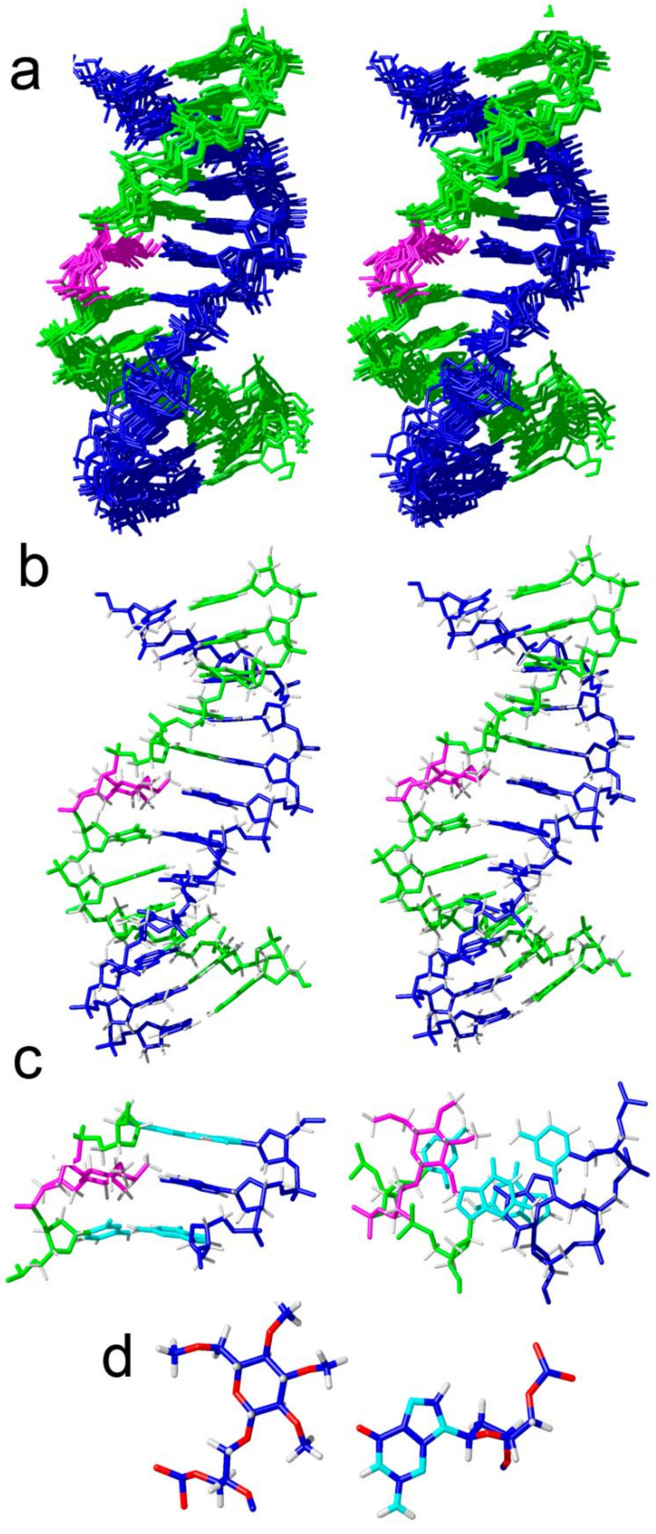

Figure S8. Structures for nucleobase pairs with **glc** and **6dglc,** when attached through different OH group to the skeleton, calculated in *vacuo* and in water and computed at BLYP-D3(BJ)/TZ2P using COSMO to simulate aqueous solution.

Attachment through OH2

X-Y	gas	water	X-Y	gas	water
6dglc-G	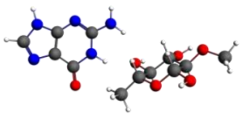	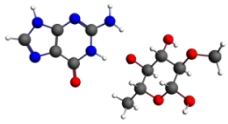	glc-G	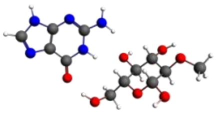	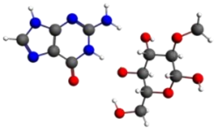
6dglc-T	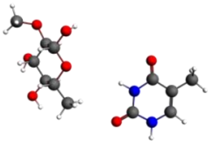	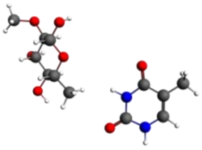	glc-T	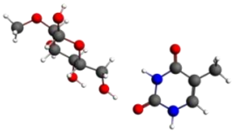	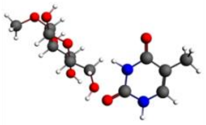
6dglc-A	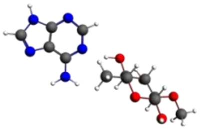	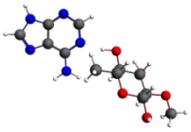	glc-A	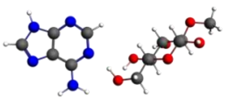	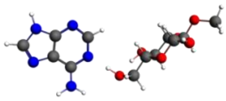
6dglc-C	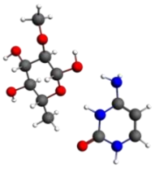	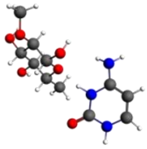	glc-C	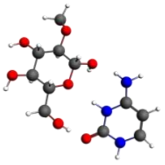	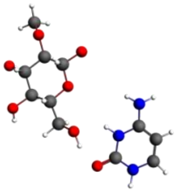

Attachment through OH3

X-Y	gas	water	X-Y	gas	water
6dglc-G	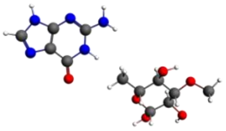	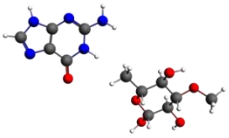	glc-G	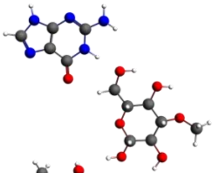	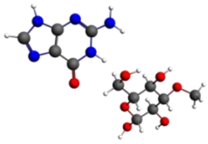
6dglc-T	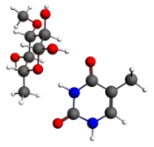	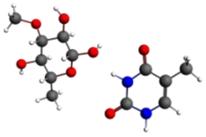	glc-T	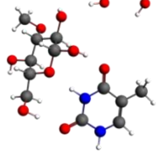	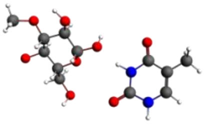
6dglc-A	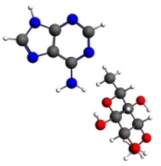	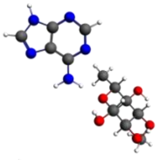	glc-A	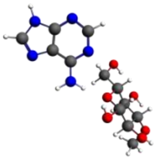	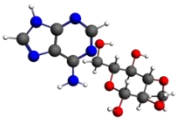
6dglc-C	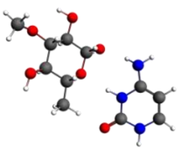	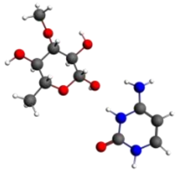	glc-C	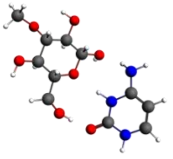	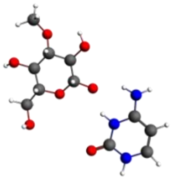

Attachment through OH4

X-Y	gas	water	X-Y	gas	water
6dglc-G	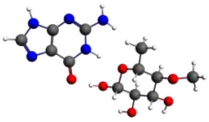	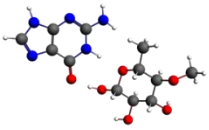	glc-G	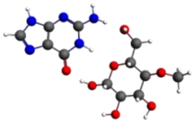	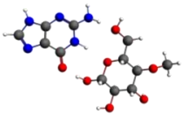
6dglc-T	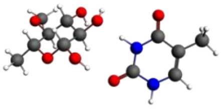	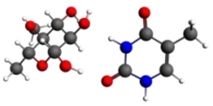	glc-T	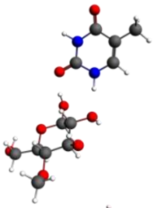	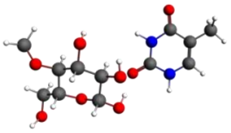
6dglc-A	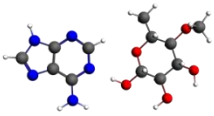	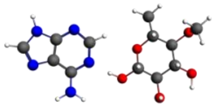	glc-A	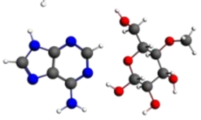	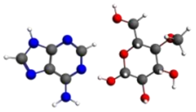
6dglc-C	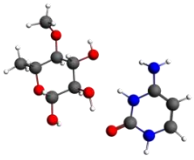	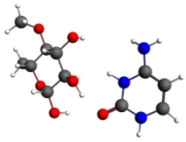	glc-C	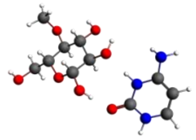	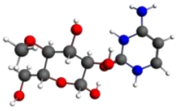

Attachment through OH6

X-Y	gas	water
glc-G	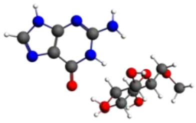	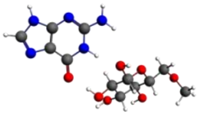
glc-T	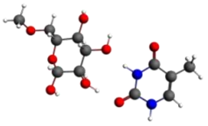	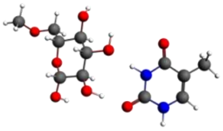
glc-A	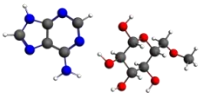	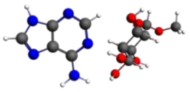
glc-C	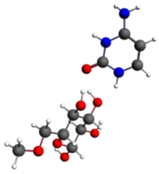	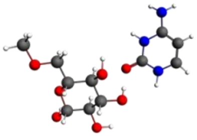

Figure S9. Gels showing DNA polymerase insertion opposite **T** and **glc** (DNA polymerases used: SIII, Bst 2.0 and Therminator). Red circles indicate the best inserted dNTP, except on Therminator where all dNTPs insert with similar efficiency. Primer and templates used:

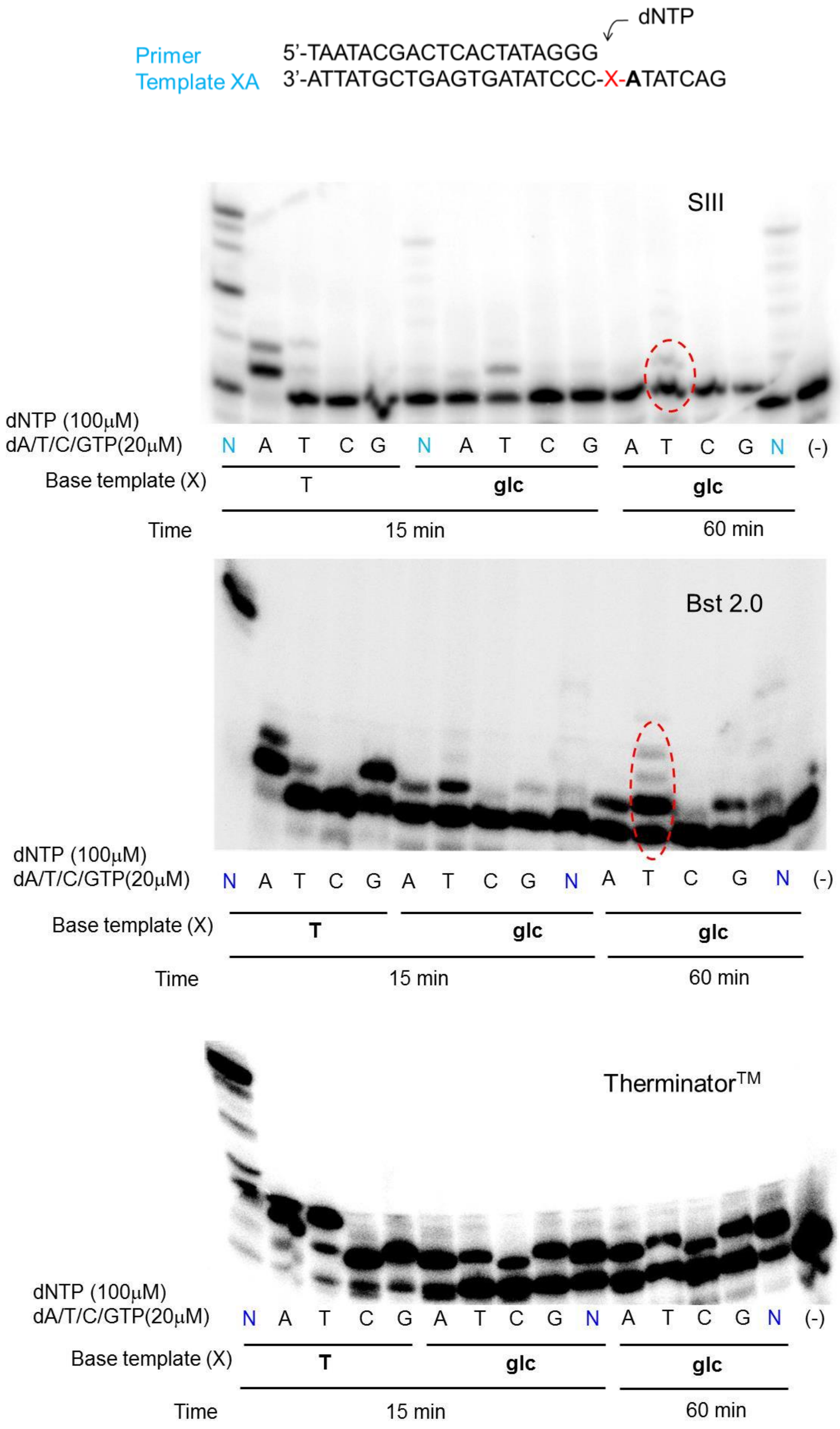

Figure S10. Gels showing DNA polymerase insertion opposite **T**A, **T***A, **glc**A and **glc**C templates (DNA polymerase used: BIOTAQ). Primer and templates used:

```
                                       dNTP
Primer         5'-TAATACGACTCACTATAGGG
Template XA    3'-ATTATGCTGAGTGATATCCC-X-ATATCAG

                                       dNTP
Primer         5'-TAATACGACTCACTATAGGG
Template XC    3'-ATTATGCTGAGTGATATCCC-X-CTATCAG
```

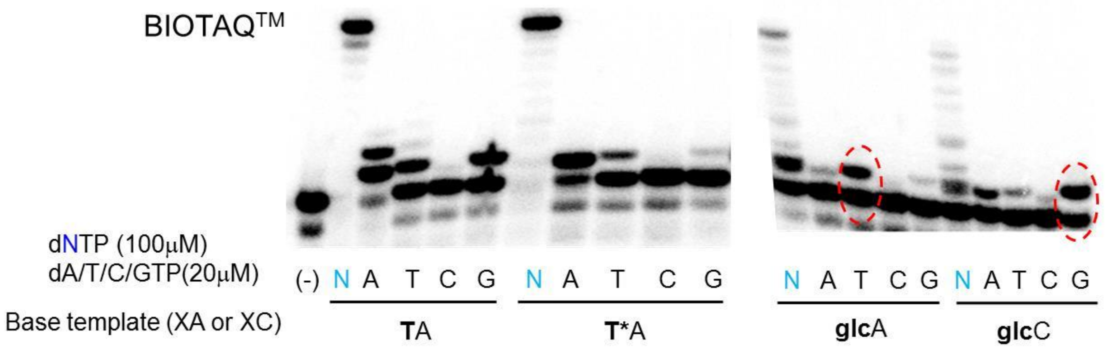

Figure S11. Gel showing DNA polymerase extension opposite **T**C, **T***C, **glcC** and **6dglcC** templates (DNA polymerase used: KF^{-}). Primer and templates used:

```
                                       dNTP
Primer         5'-TAATACGACTCACTATAGGG
Template XC    3'-ATTATGCTGAGTGATATCCC-X-CTATCAG
```

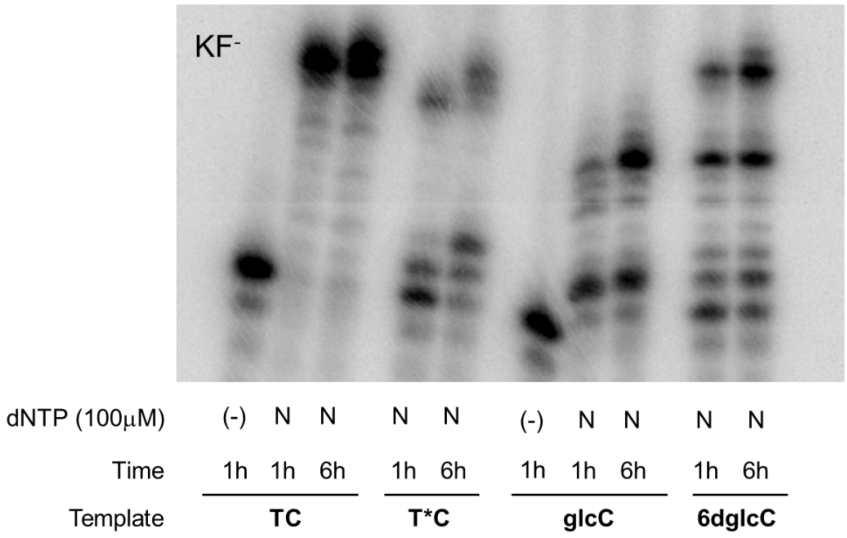

Table S1. ^{1}H-NMR assignments of **helix glc(Me)-T** (10 mM sodium phosphate, 150 mM NaCl, pH 7, T = 5ºC).

Oligonucleotide strands

helix glc(Me)-T	H1’	H2’/H2”	H3’	H4’	H5’/H5”	H5/Met	H6/H8	H1/H3
**G1**	5.71	2.65/2.82	4.88	4.22	3.71	---	7.96	
**A2**	6.33	2.77/3.00	5.08	4.50	4.24/4.18	7.99	8.38	6.28/7.74
**T3**	5.73	2.01/2.40	4.89	4.17	---	1.40	7.16	13.55
**G4**	5.59	2.68/2.76	--	--	---	--	7.89	12.59
**A5**	6.22	2.59/2.81	--	4.44	4.22	7.92	8.17	---
**C6**	5.85	2.00/2.18	4.76	4.12	---	5.29	7.32	6.86/8.25
**T7**	5.74	1.82/2.06	4.75	4.03	3.94	1.49	7.12	10.37
**G8**	5.88	2.73	4.94	4.32	4.03/3.94	---	7.99	12.98
**C9**	6.04	2.14/2.50	4.78	4.25	4.16	5.39	7.53	6.71/8.35
**T10**	5.55	2.14/2.39	4.87	4.16	4.07	1.71	7.49	14.09
**A11**	6.11	2.93/2.77	5.08	4.45	4.15/4.06	7.56	8.27	6.73/8.02
**G12**	6.03	2.48/2.26	4.66	4.20	4.30/4.14	---	7.78	
**C13**	5.91	2.18/2.58	4.70	4.13	3.81	5.97	7.90	---
**T14**	5.59	2.28/2.53	4.90	4.18	4.03	1.74	7.61	14.00
**A15**	6.14	2.80/2.96	5.09	4.45	4.19/4.06	7.58	8.28	6.65/7.98
**G16**	5.78	2.44/2.66	4.97	4.36	4.19	--	7.69	12.87
**C17**	6.10	2.20/2.28	4.80	---	---	5.38	7.40	6.58/8.17
**glc(Me)18**								
**G19**	6.01	2.76/2.88	4.93	4.34	4.00	---	8.04	12.80
**T20**	6.10	2.23/2.56	4.89	4.26	4.20/4.13	1.35	7.38	13.76
**C21**	5.62	2.23/2.49	4.88	4.18	---	5.71	7.40	6.95/8.55
**A22**	6.30	2.73/2.97	4.88	4.46	4.18	7.69	8.40	6.47/7.85
**T23**	6.02	2.05/2.49	4.86	4.16	---	1.49	7.27	13.82
**C24**	6.28	2.25	4.59	4.03	4.20	5.75	7.63	

Glc(Me) 18

H1	H2	H3	H4	H5	H6a/H6b	Met2	Met3	Met4	Met6
3.77	2.46	2.62	2.33	2.62	2.89/2.63	3.28	3.02	2.72	---

Linker

H2’/H2”	H5’/H5”	H3’
3.22/3.57	3.86	4.21

Table S2. ^{1}H-NMR assignments of **helix glc(Me)-G** (10 mM sodium phosphate, 150 mM NaCl, pH 7, T = 5ºC).

Oligonucleotide strands

helix glc(Me)-G	H1’	H2’/H2”	H3’	H4’	H5’/H5”	H5/Met	H6/H8	H1/H3
**C1**	5.89	2.17/2.56	4.69	4.11	3.81	5.96	7.89	
**T2**	5.54	2.27/2.49	4.90	4.18	4.03	1.74	7.62	14.02
**A3**	6.11	2.92/2.79	5.08	4.44	4.16/4.03	7.64	8.27	6.65/7.98
**G4**	5.65	2.52/2.44	4.96	4.33	4.19/4.05	--	7.73	12.81
**C5**	5.96	1.70/2.06	4.70	4.05		5.43	7.22	6.74/8.22
**G6**								10.34
**G7**	6.01	2.88/2.85				--	8.00	
**T8**	6.07	2.22/2.53	4.90	4.26	4.15	1.35	7.34	13.69
**C9**	5.60	2.19/2.47	4.86	4.18		5.70	7.61	6.88/8.49
**A10**	6.29	2.72/2.96		4.45	4.16	7.65	8.39	6.44/7.82
**T11**	6.00	2.03/2.46	4.85	4.17		1.47	7.25	13.81
**C12**	6.25	2.25	4.58	4.00	4.20	5.68	7.58	
**G13**	5.71	2.64/2.82	4.87	4.23	3.72	--	7.97	
**A14**	6.32	2.75/2.98	5.07	4.49	4.23/4.16	7.96	8.36	---/7.78
**T15**	5.72	2.00/2.39	4.88	4.16		1.38	7.14	13.51
**G16**	5.59	2.67/2.76				--	7.87	12.53
**A17**	6.22	2.59/2.86				7.90	8.15	
**C18**	6.04	2.17	4.83			5.34	7.38	6.62/8.13
**glc(Me)19**								
**G20**	6.02	2.86	4.97	4.19	3.88/3.79	--	8.04	12.97
**C21**	5.97	2.10/2.45	4.75	4.21	4.13	5.42	7.51	6.76/8.39
**T22**	5.47	2.13/2.35	4.85	4.14	4.21/4.04	1.71	7.47	14.08
**A23**	6.11	2.92/2.77	5.09	4.44	4.13/4.03	7.62	8.26	6.77/8.04
**G24**	6.02	2.26/2.47	4.64	4.19	4.28/4.13	--	7.77	

Glc(Me) 19

H1	H2	H3	H4	H5	H6a/H6b	Met2	Met3	Met4	Met6
3.74	2.64	2.86	2.58	2.90	---	3.36	3.25	---	---

Linker

H2’/H2”	H5’/H5”	H3’
3.19/3.35	3.88	4.35

Table S3. Structurally relevant carbohydrate-DNA NOE contacts for **helix glc(Me)-G** and **helix glc(Me)-T**.

**Carbohydrate-DNA NOEs**	**helix glc(Me)-G**	**helix glc(Me)-T**
**DNA-Glc**	G20H8-Met2: vw<br>G20H1'-Met2: vw<br>G20H8-H1: vw	T7Met-Met6: w<br>T7H2'-Met4: w<br>T7H2"-Met4: w<br>T7H6-Met4: vw<br>C17H2'-Met2: vw<br>C17H2"-Met2: vw<br>C17H1'-Met2: vw<br>C17H1'-H2: vw<br>G19H8-H1: vw
**DNA- Linker**		C17H2'-LinkH5': vw<br>C17H2"-LinkH5': vw<br>C17H1'-LinkH5': vw<br>G19H8-LinkH2': vw<br>G19H8-LinkH2": vw<br>G19H8-LinkH5': vw
**Linker-Glc**	LinkH2'-Met2: vw<br>LinkH3'-Met2: w<br>LinkH5'-Met2: vw<br>LinkH2'-H1: vw	LinkH5'-Met2: w<br>LinkH2'-H1: m<br>LinkH2"-H1: m
**Linker-Linker**	LinkH2'- LinkH5': w	LinkH2'-LinkH5': w<br>LinkH2"-LinkH5': w<br>LinkH2'-LinkH3': w<br>LinkH2"-LinkH3': w<br>LinkH3'-LinkH5': w

Table S4. NMR structural constraints and calculation statistics.

Experimental distance constraints	helix glc(Me)-G	helix glc(Me)-T
Total number	243	366
Intra-residue	129	197
Sequential	95	123
Inter-strand	11	23
**r.m.s.d.**		
Backbone heavy atoms	1.7 ± 0.8 Å	0.9 ± 0.3 Å
Base heavy atoms	1.5 ± 0.7 Å	0.7 ± 0.2 Å
All heavy atoms	1.2 ± 0.5 Å	0.8 ± 0.3 Å
**Residual violations**		
Sum of violations (Å)	16.9	17.3
Average & range	(17.9 - 16.0)	(17.7- 16.9)
Maximum violation (Å)	0.57	0.46
Average NOE energy (kcal/mol)	95.2	81.0
Range of NOE energies (kcal/mol)	90.7 – 99.4	79.5 - 82.3

Number of NOEs	helix glc(Me)-G	helix glc(Me)-T
**Glc-DNA**	3	9
**Linker-DNA**	0	6
**Glc-Linker**	4	3
**Linker-Linker**	1	5

Table S5. Cartesian coordinates (in Å) and ADF total energies (in kcal/mol) of all stationary points in this study, computed at BLYP-D3(BJ)/TZ2P using COSMO to simulate aqueous solution.

Attachment Position 2

6dglc-G

```
6dglc-G, Bond Energy       -5775.56 kcal/mol

    1.N        -7.765860    3.353794   -3.976238
    2.C        -8.550750    4.365983   -3.530698
    3.N        -9.488333    5.063466   -4.257246
    4.H        -9.718906    4.917186   -5.233862
    5.C        -8.597926    4.927667   -2.243628
    6.N        -9.549040    5.948912   -2.189651
    7.C        -7.735756    4.402716   -1.232341
    8.H       -10.828081    6.673778   -3.750277
    9.N        -6.933191    3.356944   -1.742878
   10.H        -6.310005    2.884360   -1.077821
   11.C       -10.057060    5.997711   -3.410691
   12.O        -7.629414    4.743125   -0.035137
   13.C        -6.959135    2.870317   -3.033171
   14.N        -6.076701    1.875965   -3.326101
```

15.H	-5.645612	1.370462	-2.556166
16.H	-6.229977	1.368760	-4.189215
17.C	-4.850209	3.130477	1.962841
18.H	0.461063	-0.434364	-0.547913
19.H	-4.839710	2.347688	2.730962
20.O	-0.202953	2.819198	1.300907
21.O	-5.259756	1.410983	-0.477498
22.C	-3.982779	1.469075	0.214983
23.O	-2.989126	-0.061382	-1.449580
24.H	1.216317	1.085999	0.032273
25.O	-0.486059	1.330260	-1.188567
26.C	0.709620	0.603467	-0.811099
27.O	-2.497163	2.865205	1.528710
28.H	-1.325506	0.605274	0.585545
29.C	-2.865527	1.222603	-0.809019
30.H	-2.969261	1.952597	-1.621617
31.H	-3.954720	0.687516	0.984814
32.C	-3.805324	2.843852	0.893177
33.H	-3.838743	3.623454	0.113923
34.H	-0.028837	3.751840	1.525757
35.H	-4.644817	4.096331	2.436212
36.C	-1.411113	2.755459	0.578616
37.H	-1.484238	3.581325	-0.146878
38.C	-1.481938	1.403338	-0.156255
39.H	-5.868989	0.859185	0.042524
40.H	-2.912261	-0.753005	-0.765230
41.H	1.362125	0.611527	-1.687627
42.H	-5.847884	3.177197	1.515025

6dglc-T

6dglc-T, Bond Energy -5465.24 kcal/mol

1.H	17.094914	21.425746	28.440088
2.H	26.575613	16.683853	27.142435
3.C	25.524833	18.552769	27.012520
4.C	17.046350	21.594426	26.241568
5.H	17.814422	21.737249	25.468504
6.C	16.375119	20.221710	26.019757
7.H	15.602399	20.056224	26.779665
8.C	17.414167	19.098633	26.152418
9.H	18.124432	19.167967	25.314464
10.C	18.196725	19.221692	27.481966
11.H	17.486685	19.080066	28.312727
12.C	19.341744	18.223053	27.590261
13.H	18.962417	17.196329	27.563421
14.H	19.878001	18.359744	28.534789
15.H	20.038995	18.354804	26.753518
16.O	16.698019	17.842411	26.092018
17.H	17.323838	17.150932	25.813701
18.O	15.672629	20.168650	24.761668
19.H	16.304518	20.388876	24.051497
20.O	16.157887	22.710868	26.069208
21.C	14.962930	22.709559	26.891734
22.O	18.779013	20.553666	27.582120

23.O	18.429896	22.820432	27.873363
24.C	17.782534	21.600853	27.602873
25.C	24.199372	19.152370	27.086724
26.O	23.949852	20.364252	26.948729
27.N	23.148976	18.252086	27.335452
28.H	22.211575	18.644075	27.391165
29.C	23.241059	16.881941	27.513489
30.C	26.713320	19.439527	26.748940
31.H	26.598712	19.973671	25.798174
32.H	27.634178	18.850611	26.710046
33.H	26.816617	20.199007	27.533592
34.C	25.627241	17.207075	27.185003
35.H	24.656751	15.405812	27.548839
36.O	22.258808	16.159631	27.730743
37.N	24.533623	16.405375	27.425458
38.H	14.487028	23.678413	26.725014
39.H	15.202967	22.605620	27.956469
40.H	14.273396	21.912152	26.588924
41.H	18.887837	23.118228	27.063402

6dglc-A

6dglc-A, Bond Energy -5614.37 kcal/mol

1.C	0.100516	0.396488	-0.155712
2.H	4.856960	-4.249509	0.702620
3.H	-0.642052	-0.324187	0.208013
4.H	0.312234	1.118039	0.640502
5.O	0.759836	-0.766345	-2.940054
6.H	-0.239257	-0.644964	-2.941131
7.O	2.274908	-3.143255	-2.957477
8.H	-1.171835	0.940628	-4.650030
9.O	4.407815	-3.192435	-1.066766
10.C	4.794700	-4.407123	-0.379755
11.O	1.887805	-1.022421	0.598621
12.N	-2.844524	-1.523272	-1.404641
13.C	-2.065362	0.722587	-4.070869
14.C	1.377531	-0.318307	-0.574311
15.H	2.129587	0.414735	-0.908607
16.H	-5.853196	2.313643	-4.206231
17.N	-3.162670	1.429461	-4.371866
18.C	-4.209415	1.087783	-3.588040
19.N	-5.492314	1.590996	-3.593569
20.H	-3.638109	-1.818800	-0.851754
21.H	-1.984058	-2.049437	-1.333167
22.N	-5.459212	0.029362	-1.967786
23.O	3.588364	-2.231271	1.579875
24.H	-7.233990	1.147461	-2.412934
25.C	3.183174	-1.623515	0.373306
26.H	3.893624	-0.838530	0.068728
27.C	3.096697	-2.702194	-0.725013
28.H	2.462569	-3.523898	-0.361362
29.C	2.483964	-2.093894	-1.985562
30.H	3.196116	-1.357444	-2.394556
31.N	-1.924253	-0.234701	-3.126964
32.H	1.724476	-2.738747	-3.656971

33.C	-6.194471	0.928304	-2.609720
34.H	3.905405	-1.533235	2.182058
35.C	-4.204309	0.116754	-2.571089
36.C	-2.989181	-0.572005	-2.349915
37.C	1.169773	-1.357499	-1.697074
38.H	0.421077	-2.091182	-1.354829
39.H	5.778852	-4.677729	-0.770906
40.H	4.080374	-5.213943	-0.594646
41.H	-0.326296	0.934754	-1.007982

6dglc-C

6dglc-C, Bond Energy -5260.39 kcal/mol

1.H	23.865578	16.920774	29.138322
2.O	19.769439	21.801986	27.658551
3.C	18.794614	20.800551	27.510066
4.H	18.568821	20.441333	28.522794
5.C	17.511574	21.290078	26.810808
6.H	17.798173	21.686059	25.824225
7.C	16.523957	20.120829	26.626613
8.H	16.187185	19.793437	27.617964
9.C	17.197800	18.935708	25.923811
10.H	17.430777	19.217316	24.885234
11.C	18.512742	18.547080	26.638599
12.H	18.269128	18.214641	27.659236
13.C	19.296187	17.468884	25.901785
14.H	18.685368	16.565907	25.797290
15.H	20.197891	17.200953	26.460295
16.H	19.581645	17.819824	24.902779
17.O	16.261213	17.834508	25.941881
18.H	16.519909	17.204914	25.245797
19.O	15.321153	20.545439	25.951655
20.H	15.558826	20.809824	25.042807
21.O	16.915440	22.322261	27.612276
22.C	16.575609	23.523176	26.878627
23.O	19.374838	19.726710	26.728229
24.N	23.673757	17.683210	28.498326
25.H	25.200225	19.787169	26.301136
26.C	24.415736	19.309749	26.880306
27.H	23.318360	21.053221	25.217972
28.C	23.098854	19.704598	26.713456
29.H	21.739392	20.557218	25.450768
30.H	25.728673	17.922405	27.990137
31.N	22.103341	19.083867	27.503209
32.H	21.117744	19.323085	27.349480
33.C	22.356308	18.035351	28.371330
34.O	21.435675	17.465838	28.994476
35.C	24.737415	18.301245	27.795137
36.H	19.952011	22.199317	26.784910
37.H	17.467970	23.958702	26.408375
38.H	16.169374	24.226472	27.609300
39.H	15.820337	23.313954	26.110632
40.N	22.633113	20.749887	25.906052

glc-G

glc-G, Bond Energy -5920.09 kcal/mol

1.N	-7.528840	3.112355	-4.093958
2.C	-8.365404	4.125253	-3.766782
3.N	-9.270119	4.749046	-4.596317
4.H	-9.431667	4.535964	-5.574381
5.C	-8.511018	4.765020	-2.524710
6.N	-9.489525	5.760265	-2.597845
7.C	-7.699051	4.334292	-1.432406
8.H	-10.680354	6.348572	-4.281657
9.N	-6.834446	3.279829	-1.818321
10.H	-6.238752	2.912860	-1.063787
11.C	-9.916969	5.717685	-3.849645
12.O	-7.675771	4.759654	-0.257547
13.C	-6.767578	2.709066	-3.073115
14.N	-5.871697	1.713842	-3.261716
15.H	-5.325530	1.296458	-2.507596
16.H	-5.866856	1.254373	-4.163160
17.C	-4.071426	3.302603	2.841216
18.O	-5.331873	3.992025	2.668436
19.H	-4.213741	2.362398	3.393464
20.O	0.066005	2.150110	0.919748
21.O	-5.456629	2.188309	0.474305
22.C	-4.056290	1.864361	0.716072
23.O	-3.920105	0.592692	-1.394669
24.H	-5.825857	3.494234	1.983595
25.O	-1.150319	1.359113	-1.620963
26.C	-0.107738	0.358082	-1.727544
27.O	-2.025664	2.636465	1.759917
28.H	-1.697588	0.426674	0.171190
29.C	-3.316267	1.641342	-0.608657
30.H	-3.415637	2.542763	-1.225894
31.H	-4.002732	0.956239	1.330414
32.C	-3.395833	3.017456	1.493994
33.H	-3.421624	3.935600	0.884392
34.H	0.530724	2.955787	1.211976
35.H	-3.427787	3.961586	3.429738
36.C	-1.241588	2.501694	0.543853
37.H	-1.260632	3.461842	0.005998
38.C	-1.820073	1.386420	-0.352950
39.H	-5.982943	1.380451	0.610071
40.H	-3.940234	-0.230509	-0.870591
41.H	0.265851	0.416152	-2.752447
42.H	-0.517563	-0.644226	-1.542114
43.H	0.707486	0.559589	-1.023659

glc-T

glc-T, Bond Energy -5609.92 kcal/mol

1.H	17.149912	21.364299	28.304580
2.H	25.620131	16.110267	27.339234
3.C	25.288200	18.201055	26.977125

4.C	16.929710	21.593233	26.121596
5.H	17.612723	21.875444	25.308536
6.C	16.464373	20.136432	25.893961
7.H	15.789550	19.827743	26.700092
8.C	17.674430	19.192605	25.904385
9.H	18.306274	19.417289	25.027481
10.C	18.519487	19.405736	27.181666
11.H	17.924834	19.090703	28.054993
12.C	19.849400	18.638917	27.153246
13.O	19.647767	17.207102	26.998365
14.H	20.410536	18.843675	28.069331
15.H	20.436855	18.961912	26.288134
16.O	17.187050	17.834161	25.825666
17.H	17.948048	17.266131	26.084731
18.O	15.683079	20.002931	24.689608
19.H	16.233439	20.289219	23.936054
20.O	15.868914	22.558490	26.043712
21.C	14.796450	22.411392	27.008568
22.O	18.890914	20.799674	27.314848
23.O	18.209302	22.970787	27.718014
24.C	17.742666	21.677139	27.436241
25.C	24.413011	19.316510	27.308248
26.O	24.532843	20.478845	26.879832
27.N	23.360554	19.008873	28.189558
28.H	22.752511	19.779390	28.457310
29.C	23.076643	17.780544	28.763295
30.C	26.449959	18.436617	26.048068
31.H	26.102863	18.791467	25.069891
32.H	27.022394	17.515528	25.902988
33.H	27.120606	19.206090	26.449913
34.C	25.016336	16.989125	27.532646
35.H	23.791860	15.864703	28.779273
36.O	22.124829	17.596622	29.534528
37.N	23.953530	16.785566	28.384770
38.H	14.097816	23.224563	26.799321
39.H	15.164814	22.509942	28.036828
40.H	14.278645	21.452002	26.892765
41.H	18.547243	23.369970	26.893073
42.H	19.457007	16.835820	27.879039

<u>glc-A</u>

glc-A, Bond Energy -5758.76 kcal/mol

1.C	-0.441888	-1.336461	0.254281
2.O	-1.635627	-1.880125	-0.354241
3.H	-0.316874	-1.818807	1.230045
4.H	-0.544662	-0.254865	0.407642
5.O	-0.040549	-3.667326	-1.695761
6.H	-0.871032	-3.257113	-1.348824
7.O	2.617030	-4.766268	-1.732957
8.H	-0.907266	-0.099041	-3.302017
9.O	4.679761	-2.819157	-1.688185
10.C	5.903295	-3.071662	-0.955105
11.O	1.933471	-1.100255	0.138008

12.N	-4.440866	0.178610	-0.691763
13.C	-1.874199	0.378626	-3.166114
14.C	0.801872	-1.613918	-0.606203
15.H	0.713973	-1.084888	-1.569942
16.H	-3.890356	3.149274	-5.438499
17.N	-2.246648	1.260962	-4.102603
18.C	-3.463048	1.789215	-3.839344
19.N	-4.177935	2.714937	-4.568548
20.H	-5.365810	0.540074	-0.502281
21.H	-4.059287	-0.531083	-0.078817
22.N	-5.465650	2.221914	-2.784921
23.O	4.196160	-0.668033	0.216477
24.H	-6.102989	3.627415	-4.273174
25.C	3.182356	-1.217624	-0.590061
26.H	3.090953	-0.671153	-1.541630
27.C	3.507557	-2.700311	-0.863719
28.H	3.661882	-3.207241	0.099553
29.C	2.338084	-3.352929	-1.604925
30.H	2.263247	-2.902401	-2.607590
31.N	-2.557272	0.003284	-2.063751
32.H	1.851026	-5.162821	-2.187940
33.C	-5.361860	2.936435	-3.898295
34.H	-1.967273	-1.196546	-1.027247
35.C	-4.278082	1.492550	-2.732382
36.C	-3.777786	0.549357	-1.805778
37.C	1.014570	-3.116692	-0.872695
38.H	1.047898	-3.640025	0.096987
39.H	6.692452	-3.171503	-1.704256
40.H	5.821625	-4.005672	-0.381738
41.H	6.138611	-2.242923	-0.277857
42.H	4.151319	0.303618	0.147776

<u>glc-C</u>

glc-C, Bond Energy -5403.99 kcal/mol

1.H	24.889795	15.822606	26.727176
2.O	18.767969	22.207384	27.701839
3.C	17.998704	21.027966	27.681551
4.H	17.706056	20.835136	28.722922
5.C	16.746633	21.137350	26.784711
6.H	17.083676	21.406491	25.770796
7.C	16.003402	19.786351	26.739023
8.H	15.593024	19.586233	27.736605
9.C	16.949054	18.635255	26.366401
10.H	17.264494	18.754861	25.320150
11.C	18.199332	18.657211	27.276947
12.H	17.882798	18.463972	28.314579
13.C	19.228622	17.617932	26.864844
14.H	18.723376	16.653537	26.723908
15.O	20.234170	17.506898	27.894387
16.H	19.682371	17.920265	25.908136
17.O	16.215457	17.399151	26.526758
18.H	16.547520	16.760528	25.872773
19.O	14.851179	19.836181	25.871738

20.H	15.164383	19.971315	24.957215
21.O	15.905387	22.171300	27.320440
22.C	15.242528	22.987815	26.324106
23.O	18.838252	19.958302	27.201315
24.N	24.634550	16.801733	26.796068
25.H	25.983564	19.922337	26.873592
26.C	25.241730	19.130348	26.895450
27.H	24.037716	21.462714	27.179713
28.C	23.904234	19.448462	27.050472
29.H	20.922537	16.859959	27.583173
30.H	26.654706	17.451417	26.636993
31.N	22.952572	18.396965	27.034339
32.H	22.005436	18.568363	27.380123
33.C	23.310167	17.058403	26.968032
34.O	22.446791	16.147324	27.057169
35.C	25.640813	17.796576	26.765370
36.H	18.954118	22.471858	26.780093
37.H	15.976074	23.429085	25.635693
38.H	14.731910	23.783927	26.871082
39.H	14.511225	22.402267	25.755031
40.N	23.356758	20.713480	27.282999
41.H	22.529034	20.917579	26.717811

Attachment Position 3

6dglc-G

6dglc-G, Bond Energy -5772.69 kcal/mol

1.N	-9.387996	2.936418	-4.007731
2.C	-9.921997	4.134202	-3.661003
3.N	-11.160125	4.617619	-4.013319
4.H	-11.851127	4.138402	-4.579591
5.C	-9.345130	5.149025	-2.879116
6.N	-10.219878	6.230227	-2.757037
7.C	-8.034758	4.948062	-2.350144
8.H	-12.184807	6.463749	-3.580884
9.N	-7.522444	3.682856	-2.737127
10.H	-6.598758	3.467693	-2.372468
11.C	-11.290475	5.872907	-3.447577
12.O	-7.343562	5.704239	-1.638694
13.C	-8.168638	2.745628	-3.513837
14.N	-7.523591	1.563749	-3.724961
15.H	-6.517170	1.521618	-3.619256
16.H	-7.911501	0.964822	-4.445052
17.C	-4.372951	3.105563	-1.461034
18.H	-4.006199	3.098489	-2.491944
19.H	-5.101770	2.293731	-1.333547
20.O	-3.387125	2.882932	3.134253
21.O	-1.897774	1.551593	-2.014606
22.C	-2.480231	1.569548	-0.693475
23.O	-0.794212	0.090299	0.128712
24.C	0.625147	0.014273	0.418145
25.O	-0.928260	1.459514	2.747214
26.H	-1.342758	1.590015	3.621041

27.O	-3.771414	2.921367	0.854417
28.H	-2.707050	0.697978	1.939904
29.C	-1.387040	1.383684	0.369389
30.H	-0.623094	2.165239	0.232080
31.H	-3.210610	0.750902	-0.596289
32.C	-3.216084	2.904891	-0.491787
33.H	-2.491969	3.728409	-0.594322
34.H	-3.563621	3.811938	3.370193
35.H	-4.857748	4.068180	-1.264584
36.C	-2.752091	2.847358	1.874744
37.H	-2.059215	3.695214	1.757507
38.C	-1.989456	1.514731	1.771088
39.H	-1.300209	0.778620	-2.027898
40.H	0.947895	-0.980597	0.103488
41.H	0.815636	0.148997	1.487386
42.H	1.174231	0.775841	-0.151645

6dglc-T

6dglc-T, Bond Energy -5469.19 kcal/mol

1.H	18.786527	20.028087	28.359360
2.H	25.852760	16.344198	27.218089
3.C	25.086580	18.351371	27.214205
4.C	18.414953	21.474413	26.766790
5.H	18.804449	21.639779	25.748508
6.C	16.895491	21.264714	26.684760
7.H	16.496205	21.164868	27.706476
8.C	16.569545	19.990354	25.898253
9.H	16.866164	20.129403	24.848567
10.C	17.352797	18.792039	26.490234
11.H	17.022848	18.645936	27.531564
12.C	17.170716	17.505450	25.696295
13.H	16.116953	17.206547	25.693797
14.H	17.752060	16.695734	26.149823
15.H	17.500798	17.644024	24.659167
16.O	15.141269	19.770365	25.984085
17.H	14.886289	19.170510	25.261043
18.O	16.360835	22.459273	26.075306
19.N	23.777541	16.347700	27.375232
20.O	18.729077	22.602136	27.607172
21.H	20.812490	20.771927	26.531503
22.O	18.779321	19.101647	26.489867
23.O	20.510148	20.356784	27.363170
24.C	19.103386	20.224489	27.326496
25.C	23.854643	19.126987	27.272509
26.O	23.790883	20.370614	27.215299
27.N	22.673473	18.379367	27.402895
28.H	21.797139	18.915781	27.446680
29.C	22.563686	17.003428	27.456265
30.C	26.400708	19.078371	27.101726
31.H	26.412882	19.729382	26.219694
32.H	27.231277	18.370692	27.027342
33.H	26.566837	19.720562	27.975604
34.C	24.987681	16.995583	27.264241
35.H	23.747521	15.334140	27.409285

36.O	21.480235	16.409485	27.566246
37.H	18.142052	23.324477	27.309067
38.C	15.072719	22.880168	26.593115
39.H	15.140119	23.085666	27.670415
40.H	14.308975	22.118024	26.408557
41.H	14.817908	23.799963	26.061009

6dglc-A

6dglc-A, Bond Energy -5608.65 kcal/mol

1.C	-0.184152	-2.522039	-2.988857
2.H	2.877935	-5.191455	-0.111793
3.H	-0.740000	-3.294600	-2.443669
4.H	-0.845851	-1.678331	-3.204044
5.O	2.569182	-3.666893	-3.114594
6.H	3.396520	-4.130893	-2.878419
7.O	4.171276	-3.644714	-0.765264
8.H	-1.035081	1.677696	-4.878845
9.O	3.621482	-1.483268	1.157382
10.C	3.831108	-4.717559	0.151577
11.O	0.512233	-1.559711	-0.891137
12.N	-1.994022	-0.015577	-0.929795
13.C	-1.826438	1.489929	-4.156769
14.C	1.012557	-2.056395	-2.172313
15.H	1.532968	-1.236628	-2.691871
16.H	-5.857336	2.340076	-4.284136
17.N	-3.058019	1.903297	-4.511816
18.C	-3.969173	1.631054	-3.553637
19.N	-5.325108	1.890665	-3.547401
20.H	-2.715099	-0.361223	-0.307905
21.H	-1.095172	-0.503121	-0.920150
22.N	-4.892336	0.870957	-1.583184
23.O	0.917770	-0.490438	1.110546
24.H	-6.872955	1.504552	-2.105010
25.C	1.540176	-0.957777	-0.061358
26.H	2.010919	-0.133120	-0.619394
27.C	2.585584	-2.025058	0.318746
28.H	2.073750	-2.778534	0.925726
29.C	3.149280	-2.643430	-0.972726
30.H	3.672299	-1.853446	-1.531221
31.N	-1.443345	0.870736	-3.022869
32.H	0.551000	0.396409	0.940996
33.C	-5.824715	1.418714	-2.352569
34.H	3.786536	-4.351385	1.184287
35.C	-3.715117	0.996646	-2.324310
36.C	-2.380655	0.600442	-2.074512
37.C	2.019873	-3.177380	-1.870560
38.H	1.482130	-3.988942	-1.357826
39.H	4.204196	-0.927521	0.606077
40.H	4.634542	-5.451379	0.060733
41.H	0.161260	-2.944772	-3.937781

6dglc-C

6dglc-C, Bond Energy -5261.02 kcal/mol

1.H	24.424364	15.742869	26.402659
2.O	20.464004	20.235228	26.238259
3.C	19.131103	20.203712	26.709186
4.H	19.197171	20.090707	27.799786
5.C	18.352806	21.469835	26.335579
6.H	18.380236	21.565623	25.237818
7.C	16.887696	21.358587	26.783499
8.H	16.853307	21.317132	27.884119
9.C	16.255848	20.081651	26.219364
10.H	16.205845	20.158161	25.123662
11.C	17.123812	18.857243	26.602689
12.H	17.143640	18.779682	27.701325
13.C	16.626949	17.554429	25.991040
14.H	15.614559	17.330007	26.343509
15.H	17.279894	16.725836	26.284507
16.H	16.611976	17.626261	24.896538
17.O	14.923006	19.962596	26.769549
18.H	14.419211	19.350954	26.203849
19.O	16.241923	22.566793	26.328838
20.H	22.233402	20.795822	27.393828
21.O	18.989389	22.606680	26.949541
22.N	22.991710	20.441031	27.977931
23.O	18.488410	19.061282	26.123397
24.N	24.188397	16.673064	26.729450
25.H	25.546804	19.418076	28.216361
26.C	24.815662	18.768562	27.744502
27.H	23.695899	21.155118	28.143761
28.C	23.523452	19.222767	27.544664
29.H	14.873253	24.017340	26.775253
30.H	26.157693	17.036149	27.457954
31.N	22.578781	18.339668	26.963436
32.H	21.684870	18.725466	26.652701
33.C	22.904821	17.077818	26.487384
34.O	22.064975	16.366312	25.894739
35.C	25.176505	17.472970	27.356746
36.H	20.458413	20.458819	25.286582
37.H	18.365097	23.349834	26.833975
38.C	15.207210	23.072324	27.210392
39.H	15.612550	23.253605	28.215020
40.H	14.368769	22.371724	27.272313

glc-G

glc-G, Bond Energy -5920.01 kcal/mol

1.N	-9.086505	2.739935	-4.395108
2.C	-9.774918	3.794595	-3.892934
3.N	-11.097848	4.099814	-4.116667
4.H	-11.745163	3.573246	-4.692705
5.C	-9.307569	4.806390	-3.038674
6.N	-10.330492	5.711156	-2.746502

7.C	-7.942840	4.773525	-2.616498
8.H	-12.365056	5.699313	-3.423117
9.N	-7.261280	3.664659	-3.171370
10.H	-6.266934	3.565837	-2.903674
11.C	-11.380952	5.253895	-3.409363
12.O	-7.336813	5.566005	-1.864407
13.C	-7.817009	2.712188	-3.994128
14.N	-7.013790	1.671500	-4.360176
15.H	-6.008930	1.782764	-4.295633
16.H	-7.343970	1.097977	-5.127988
17.C	-4.831726	3.179124	-0.788876
18.O	-4.611284	3.381968	-2.210593
19.H	-5.471785	2.301256	-0.622868
20.O	-3.054545	2.898528	3.545578
21.O	-2.453905	1.737135	-1.816687
22.C	-2.794073	1.684304	-0.407591
23.O	-1.001486	0.188283	0.088169
24.C	0.446152	0.104309	0.129989
25.O	-0.682233	1.522587	2.711182
26.H	-0.941822	1.619884	3.647031
27.O	-3.830535	2.972446	1.368978
28.H	-2.564051	0.749655	2.203657
29.C	-1.535756	1.477727	0.445394
30.H	-0.804098	2.262026	0.195926
31.H	-3.486841	0.849325	-0.227738
32.C	-3.507457	2.998540	-0.041182
33.H	-2.844440	3.849928	-0.262698
34.H	-3.198322	3.819544	3.829679
35.H	-5.348848	4.069626	-0.423712
36.C	-2.644791	2.897704	2.198594
37.H	-1.999019	3.761053	1.977792
38.C	-1.894828	1.580067	1.933079
39.H	-2.063908	0.873075	-2.047975
40.H	0.703910	-0.893383	-0.233083
41.H	0.817617	0.237877	1.151044
42.H	0.894141	0.861687	-0.527635
43.H	-3.955736	2.704137	-2.490612

glc-T

glc-T, Bond Energy -5613.13 kcal/mol

1.H	18.985319	19.839019	28.030302
2.H	25.851145	16.459729	28.023838
3.C	25.172258	18.337403	27.229391
4.C	18.386832	21.465505	26.701006
5.H	18.641324	21.775479	25.674193
6.C	16.874934	21.208162	26.780470
7.H	16.611610	20.960047	27.821105
8.C	16.467800	20.035586	25.878485
9.H	16.596655	20.331296	24.829125
10.C	17.365476	18.806237	26.178785
11.H	17.161970	18.467410	27.208238
12.C	17.105210	17.663518	25.201370
13.H	16.025976	17.473088	25.157526

14.O	17.820089	16.451751	25.534150
15.H	17.452482	17.954332	24.204330
16.O	15.079366	19.722604	26.141971
17.H	14.659400	19.466285	25.303252
18.O	16.242680	22.449995	26.407992
19.N	23.785239	16.637755	28.200955
20.O	18.779981	22.484171	27.639742
21.H	20.759750	20.791799	26.034194
22.O	18.766488	19.169666	26.061822
23.O	20.561994	20.360937	26.889083
24.C	19.166549	20.181151	27.002637
25.C	23.978530	19.140760	27.000082
26.O	23.967674	20.268740	26.468238
27.N	22.771738	18.565854	27.424651
28.H	21.918077	19.119697	27.268438
29.C	22.605000	17.335275	28.028054
30.C	26.511834	18.883207	26.809466
31.H	26.529360	19.092618	25.732784
32.H	27.310052	18.171677	27.040374
33.H	26.726681	19.828654	27.322900
34.C	25.016941	17.120769	27.818656
35.H	23.712540	15.725730	28.639737
36.O	21.500130	16.895566	28.382521
37.H	18.148914	23.219143	27.509827
38.C	15.003534	22.740798	27.103995
39.H	15.156062	22.705044	28.191187
40.H	14.218922	22.032586	26.819504
41.H	14.720448	23.753556	26.807742
42.H	17.465823	16.118401	26.378637

glc-A

glc-A, Bond Energy -5752.58 kcal/mol

1.C	-0.563306	-2.986183	-2.628821
2.O	-0.374117	-3.144844	-4.053869
3.H	-0.663426	-3.964671	-2.136691
4.H	-1.494842	-2.430583	-2.496762
5.O	2.324449	-3.196443	-3.357215
6.H	3.204883	-3.617766	-3.333002
7.O	4.270379	-2.878727	-1.268985
8.H	-0.279811	1.596540	-3.876093
9.O	3.495444	-1.031221	0.898379
10.C	4.405622	-4.027371	-0.390823
11.O	0.216808	-1.968558	-0.600052
12.N	-2.337769	-0.461739	-0.575261
13.C	-1.244461	1.388302	-3.419170
14.C	0.591147	-2.207352	-1.984887
15.H	0.709696	-1.244612	-2.502529
16.H	-5.050911	2.545327	-4.495660
17.N	-2.316008	1.932795	-4.028247
18.C	-3.463082	1.622500	-3.388719
19.N	-4.758050	1.975600	-3.709974
20.H	-3.211460	-0.833347	-0.221485
21.H	-1.502625	-1.034596	-0.443785

22.N	-4.917484	0.707305	-1.851751
23.O	0.661934	-0.876237	1.382621
24.H	-6.654546	1.537108	-2.795581
25.C	1.163597	-1.102107	0.090494
26.H	1.250915	-0.162788	-0.476326
27.C	2.522729	-1.821232	0.193215
28.H	2.367906	-2.716784	0.803174
29.C	2.999349	-2.193932	-1.223863
30.H	3.183813	-1.263499	-1.780412
31.N	-1.210835	0.616714	-2.315796
32.H	0.047222	-0.120207	1.355278
33.C	-5.582633	1.405319	-2.763671
34.H	0.553604	-3.434683	-4.182103
35.C	-3.578776	0.833524	-2.228993
36.C	-2.379853	0.311938	-1.692996
37.C	1.918229	-2.983118	-1.981902
38.H	1.748102	-3.953866	-1.494091
39.H	3.762726	-0.288182	0.325158
40.H	5.331804	-4.522638	-0.689687
41.H	3.570396	-4.728421	-0.505647
42.H	4.482134	-3.714181	0.657174

glc-C

glc-C, Bond Energy -5403.39 kcal/mol

1.H	24.085210	15.543410	27.515665
2.O	20.443575	20.116691	26.259477
3.C	19.101320	20.170064	26.698640
4.H	19.137943	20.117824	27.795533
5.C	18.378560	21.438712	26.234391
6.H	18.417429	21.463797	25.132978
7.C	16.908783	21.407576	26.680455
8.H	16.870641	21.433251	27.781458
9.C	16.218008	20.125853	26.195484
10.H	16.138167	20.154916	25.100971
11.C	17.053921	18.888608	26.616541
12.H	17.043503	18.819185	27.717264
13.C	16.500396	17.601895	26.008717
14.H	15.426903	17.538322	26.225895
15.O	17.181801	16.411419	26.466361
16.H	16.639808	17.628643	24.922752
17.O	14.899948	20.066099	26.790276
18.H	14.290327	19.675764	26.140671
19.O	16.310078	22.608487	26.152789
20.H	22.402215	20.849966	26.899945
21.O	19.052269	22.587923	26.782464
22.H	17.007597	16.313145	27.420453
23.O	18.424439	19.024452	26.157464
24.N	23.975856	16.550879	27.482881
25.H	25.770682	19.443017	27.453748
26.C	24.920078	18.768999	27.430893
27.H	24.025412	21.262513	27.071964
28.C	23.638870	19.284400	27.335816
29.N	23.282288	20.635832	27.369550

30.H	26.076415	16.891284	27.582181
31.N	22.542528	18.384240	27.348289
32.H	21.634554	18.733323	27.033778
33.C	22.691873	17.002698	27.354855
34.O	21.704545	16.241307	27.267333
35.C	25.119821	17.386292	27.519809
36.H	20.457900	20.204507	25.285749
37.H	18.449852	23.342154	26.627756
38.C	15.271640	23.190413	26.982425
39.H	15.644638	23.359240	28.001393
40.H	14.388924	22.544161	27.016677
41.H	15.018557	24.148593	26.523024

Attachment Position 4

6dglc-G

6dglc-G, Bond Energy -5780.22 kcal/mol

1.C	2.757810	-1.795150	-1.763362
2.H	3.744285	-1.877940	-2.228966
3.H	2.286966	-2.786058	-1.769153
4.H	2.154035	-1.105094	-2.360603
5.O	5.149024	-2.158801	-0.015174
6.C	5.709700	-3.465235	-0.281585
7.O	4.647173	-2.570316	2.826394
8.H	4.204841	-3.439055	2.868840
9.O	2.521526	-0.925135	3.850948
10.H	1.606327	-0.740647	4.136369
11.O	1.577144	-1.237237	0.269126
12.H	1.928433	-2.497477	2.596157
13.C	3.850928	-1.701339	1.991105
14.H	4.367838	-0.734464	2.022027
15.H	3.415483	-3.211055	0.498498
16.C	2.907862	-1.285410	-0.338143
17.H	3.327641	-0.267500	-0.342983
18.C	3.815502	-2.186837	0.529703
19.H	-4.077075	2.046072	-3.745840
20.C	1.609233	-0.629144	1.598534
21.H	2.068951	0.368419	1.505635
22.C	2.433024	-1.519102	2.539434
23.O	0.302155	-0.544274	2.080169
24.O	-0.743434	1.498566	0.715966
25.C	-1.188911	-0.442970	-2.282705
26.N	-0.596595	-1.564743	-2.773956
27.H	-0.107280	-2.185196	-2.141257
28.H	-1.051612	-2.006705	-3.564031
29.N	-2.138824	0.167312	-2.987710
30.C	-2.598554	1.294731	-2.398018
31.N	-3.572709	2.141379	-2.871145
32.N	-2.933484	3.005747	-0.892481
33.H	-4.447217	3.946302	-2.082129
34.N	-0.723119	0.029661	-1.072749
35.H	0.055095	-0.467736	-0.614703
36.C	-3.734116	3.148282	-1.935705

37.C	-2.207103	1.844356	-1.164527
38.C	-1.204350	1.176004	-0.412420
39.H	6.699317	-3.294659	-0.712608
40.H	5.089554	-4.020188	-0.999517
41.H	5.808179	-4.046287	0.644719
42.H	-0.125985	0.251938	1.642753

6dglc-T

6dglc-T, Bond Energy -5475.54 kcal/mol

1.H	17.588137	18.661628	28.555115
2.H	25.408966	16.996238	25.818166
3.C	24.746073	18.592424	27.095187
4.C	18.886145	19.703144	27.149833
5.H	19.343407	19.478421	26.173414
6.C	17.941872	20.903415	27.010178
7.H	17.518170	21.137491	27.994640
8.C	16.789780	20.580995	26.047654
9.H	17.187014	20.455479	25.029712
10.C	16.097738	19.263612	26.475628
11.H	15.646939	19.424421	27.467900
12.C	15.035424	18.805135	25.486219
13.H	14.262234	19.573982	25.389632
14.H	14.930492	23.131538	24.986992
15.H	15.480528	18.627173	24.499636
16.O	15.828981	21.654914	26.078060
17.H	19.046209	21.959770	25.744609
18.O	18.684605	22.090335	26.642555
19.N	23.331199	17.036834	25.941635
20.O	19.926866	19.985196	28.119044
21.H	19.618235	17.299189	27.097151
22.O	17.091884	18.201127	26.582340
23.O	18.879630	17.321231	27.765721
24.C	18.094369	18.465876	27.599098
25.C	23.552936	19.229030	27.638004
26.O	23.550043	20.218362	28.394059
27.N	22.330606	18.651176	27.255168
28.H	21.474054	19.105080	27.616857
29.C	22.160463	17.576668	26.417001
30.C	26.098660	19.142932	27.463015
31.H	26.199331	20.184029	27.133231
32.H	26.896127	18.552140	27.003626
33.H	26.238839	19.136737	28.550463
34.C	24.577924	17.525635	26.268949
35.H	23.246848	16.238060	25.321673
36.O	21.039773	17.118750	26.103368
37.H	20.167868	20.925718	27.995176
38.H	15.288778	21.657480	24.040552
39.C	15.663635	22.340058	24.815399
40.H	16.610636	22.785457	24.483983
41.H	14.563855	17.880436	25.835646

6dglc-A

6dglc-A, Bond Energy -5613.27 kcal/mol

1.C	1.481204	-3.688366	-3.441232
2.H	4.422334	-2.264468	-3.192703
3.H	1.035729	-4.611060	-3.049787
4.H	2.388014	-3.949532	-3.995617
5.O	4.014544	-3.844105	-1.841308
6.C	4.906216	-2.847788	-2.399107
7.O	3.847131	-2.975421	0.885465
8.H	-0.889492	-0.996342	-3.363531
9.O	1.887296	-0.964492	1.557840
10.H	2.289644	-0.162509	1.171313
11.O	0.572063	-2.408720	-1.620841
12.N	-3.329517	1.298859	-0.509776
13.C	-1.790632	-0.399361	-3.251211
14.C	1.817068	-2.736462	-2.302396
15.H	2.246982	-1.806083	-2.703710
16.H	-4.746535	0.646248	-5.915176
17.N	-2.549155	-0.228365	-4.342317
18.C	-3.653692	0.505094	-4.076643
19.N	-4.672521	0.872903	-4.929607
20.H	-4.201315	1.758070	-0.281329
21.H	-2.714361	1.030165	0.247935
22.N	-5.219245	1.740661	-2.922280
23.O	-0.524877	-1.106716	-0.061306
24.H	-6.476139	2.008215	-4.637889
25.C	0.729908	-1.380398	-0.601971
26.H	1.167782	-0.486372	-1.084581
27.C	1.666651	-1.917214	0.491566
28.H	1.175673	-2.774250	0.965760
29.C	3.009015	-2.358183	-0.116217
30.H	3.511691	-1.458403	-0.507878
31.N	-2.014524	0.071716	-2.004568
32.H	-1.069204	-0.619963	-0.766230
33.C	-5.578500	1.604742	-4.192409
34.H	3.845637	-2.373904	1.654626
35.C	-4.009106	1.053510	-2.831393
36.C	-3.125996	0.816012	-1.753384
37.C	2.785768	-3.353938	-1.261965
38.H	2.309083	-4.253701	-0.853405
39.H	5.743353	-3.406429	-2.824544
40.H	5.284843	-2.166959	-1.626454
41.H	0.773208	-3.217248	-4.130567

6dglc-C

6dglc-C, Bond Energy -5261.68 kcal/mol

1.H	23.330723	16.027922	27.173298
2.O	17.805785	16.836671	26.944555
3.C	17.314526	18.155372	27.039543
4.H	16.769926	18.212252	27.992451
5.C	18.438722	19.212981	27.004066
6.H	18.982858	19.090080	26.050753

7.C	17.837346	20.626593	27.058035
8.H	17.396550	20.763695	28.052059
9.C	16.754613	20.792599	25.969955
10.H	17.242808	20.717187	24.988570
11.C	15.706645	19.654457	26.054404
12.H	15.206923	19.682793	27.033981
13.C	14.674400	19.713871	24.937813
14.H	14.108349	20.648571	24.996568
15.H	13.975233	18.875377	25.024662
16.H	15.167045	19.668352	23.958843
17.O	16.162638	22.108932	25.962329
18.H	19.887206	18.238842	27.937885
19.O	18.856419	21.645145	26.963864
20.H	22.619716	21.573178	26.573384
21.O	19.333290	19.050502	28.113631
22.N	23.366983	21.181963	27.152201
23.O	16.403879	18.377458	25.943381
24.N	23.383748	17.039314	27.119482
25.H	25.607571	19.590027	26.793761
26.C	24.664483	19.068648	26.922952
27.H	24.240123	21.669976	26.965292
28.C	23.494507	19.794092	27.060028
29.H	15.068578	23.505156	26.963840
30.H	25.492873	17.021878	26.843497
31.N	22.270374	19.085567	27.180408
32.H	21.438062	19.578952	27.505409
33.C	22.208366	17.702911	27.280314
34.O	21.119641	17.111175	27.499125
35.C	24.637533	17.670837	26.946852
36.H	18.444726	16.791613	26.206334
37.H	19.216736	21.628397	26.057422
38.C	15.417439	22.486189	27.146454
39.H	16.048523	22.478703	28.043365
40.H	14.549572	21.834467	27.306510

glc-G

glc-G, Bond Energy -5925.95 kcal/mol

1.N	-8.597259	2.830842	-4.094977
2.C	-9.222911	3.909967	-3.572667
3.N	-10.437832	4.430205	-3.951093
4.H	-11.043766	4.070308	-4.680424
5.C	-8.776884	4.747050	-2.534700
6.N	-9.707759	5.757929	-2.284786
7.C	-7.539833	4.446082	-1.906029
8.H	-11.582196	6.120065	-3.258391
9.N	-6.914092	3.322451	-2.476292
10.H	-6.029200	3.026549	-2.046394
11.C	-10.681367	5.533547	-3.151930
12.O	-7.000408	5.050874	-0.939090
13.C	-7.426159	2.567450	-3.517194
14.N	-6.686765	1.495572	-3.910222
15.H	-5.667331	1.566239	-3.796834
16.H	-6.997356	1.059862	-4.772458
17.C	-3.168262	1.375184	-2.587390

18.H	-2.148526	1.156159	-2.914316
19.H	-3.685673	0.433422	-2.358325
20.O	-3.886439	2.088649	-3.631890
21.O	-0.844184	1.573802	-0.772405
22.C	-0.200655	0.280305	-0.680318
23.O	-1.443423	1.792363	2.076655
24.H	-1.856090	0.939119	2.309407
25.O	-3.677544	3.509381	2.634141
26.H	-4.611514	3.712527	2.833520
27.O	-4.457967	2.385220	-0.824595
28.H	-4.153277	1.674929	1.736163
29.C	-2.238058	2.408992	1.041374
30.H	-1.761801	3.380841	0.863914
31.H	-2.545326	0.577922	-0.071989
32.C	-3.105168	2.239939	-1.341870
33.H	-2.708238	3.232308	-1.605845
34.H	-6.277649	4.056118	0.169038
35.H	-0.103609	-0.035682	0.366082
36.C	-4.501633	3.264689	0.343436
37.H	-4.080457	4.240768	0.051833
38.C	-3.683812	2.639801	1.483862
39.O	-5.826072	3.382808	0.761861
40.C	-2.192425	1.600715	-0.268180
41.H	0.791767	0.398796	-1.121869
42.H	-0.763225	-0.477811	-1.243182
43.H	-3.508840	1.825028	-4.489083

glc-T

glc-T, Bond Energy -5615.62 kcal/mol

1.H	17.159203	18.011319	28.231170
2.H	24.714197	16.564546	27.868476
3.C	24.965777	18.512857	26.999728
4.C	18.533721	19.617513	27.699847
5.H	19.316138	19.771925	26.939506
6.C	17.453101	20.697983	27.544674
7.H	16.708689	20.573413	28.340866
8.C	16.739026	20.583893	26.190571
9.H	17.446482	20.822013	25.382644
10.C	16.230335	19.132196	26.000805
11.H	15.465438	18.930874	26.769627
12.C	15.629318	18.914078	24.614802
13.H	14.904667	19.710439	24.411754
14.O	15.008562	17.614522	24.466152
15.H	16.424900	18.956984	23.863623
16.O	15.626078	21.499193	26.163975
17.H	18.659788	22.195879	27.062595
18.O	18.017024	22.012005	27.774403
19.N	22.936680	17.229812	27.013464
20.O	19.113221	19.659837	29.021235
21.H	19.668800	17.440772	27.007741
22.O	17.327408	18.197342	26.154428
23.O	18.871781	17.187187	27.544283
24.C	17.929750	18.221161	27.475624
25.C	24.266022	19.566948	26.278228

26.O	24.766663	20.644492	25.911771
27.N	22.913914	19.298849	25.994099
28.H	22.400154	20.017030	25.487527
29.C	22.205107	18.164464	26.322996
30.C	26.419793	18.706459	27.338444
31.H	27.012739	18.865421	26.429861
32.H	26.814138	17.833339	27.865764
33.H	26.558361	19.591324	27.971387
34.C	24.267365	17.395187	27.334964
35.H	22.458172	16.372149	27.268143
36.O	21.008555	18.007831	26.012265
37.H	19.295174	20.600864	29.210505
38.H	14.186095	17.616134	24.988900
39.C	15.668526	22.458156	25.081086
40.H	16.559556	23.095311	25.157280
41.H	14.770162	23.072497	25.177807
42.H	15.661494	21.955748	24.104165

glc-A

glc-A, Bond Energy -5756.53 kcal/mol

1.C	1.335117	-3.704052	-3.433088
2.H	4.230497	-2.025092	-3.241284
3.H	0.838306	-4.593674	-3.020312
4.O	0.450318	-3.003777	-4.345309
5.O	3.984295	-3.761782	-2.050848
6.C	4.802284	-2.688623	-2.581699
7.O	3.949893	-3.026197	0.726042
8.H	-1.184697	-1.518487	-3.083284
9.O	1.973009	-1.113199	1.599814
10.H	2.309709	-0.278346	1.220120
11.O	0.505700	-2.510893	-1.525179
12.N	-3.070963	1.500572	-0.487797
13.C	-1.961507	-0.760374	-3.024777
14.C	1.703054	-2.761316	-2.302433
15.H	2.057948	-1.810128	-2.727276
16.H	-4.751950	0.514738	-5.764599
17.N	-2.717133	-0.578274	-4.117231
18.C	-3.648958	0.383024	-3.932757
19.N	-4.599784	0.844382	-4.817936
20.H	-3.724923	2.261717	-0.362430
21.H	-2.385802	1.325683	0.236413
22.N	-4.893158	2.044365	-2.932139
23.O	-0.529569	-1.247255	0.105158
24.H	-6.122589	2.353797	-4.661687
25.C	0.698517	-1.490114	-0.503863
26.H	1.090229	-0.582416	-1.000199
27.C	1.712067	-2.032696	0.514278
28.H	1.277894	-2.921415	0.984913
29.C	3.027489	-2.405079	-0.194773
30.H	3.470986	-1.473427	-0.582995
31.N	-2.048618	-0.107710	-1.842868
32.H	-1.113578	-0.773900	-0.579594
33.C	-5.313214	1.831475	-4.172741
34.H	3.974561	-2.455581	1.518159

35.C	-3.844792	1.139313	-2.763915
36.C	-2.987925	0.862782	-1.673733
37.C	2.778511	-3.368728	-1.364465
38.H	2.382264	-4.308322	-0.961055
39.H	5.589433	-3.175774	-3.161371
40.H	5.259961	-2.099892	-1.777009
41.H	2.252285	-4.016922	-3.945998
42.H	0.222743	-3.620645	-5.062768

glc-C

glc-C, Bond Energy -5406.00 kcal/mol

1.H	21.680395	16.142231	27.608878
2.O	18.431938	17.237058	27.527452
3.C	17.634202	18.382622	27.449938
4.H	16.867167	18.302301	28.233938
5.C	18.418267	19.697119	27.606055
6.H	19.184940	19.731212	26.815634
7.C	17.473194	20.901285	27.452405
8.H	16.754274	20.877399	28.279377
9.C	16.730966	20.829927	26.105784
10.H	17.470404	20.883261	25.295741
11.C	16.012349	19.457091	25.995414
12.H	15.258803	19.361777	26.793653
13.C	15.342202	19.280074	24.635732
14.H	14.707775	20.150503	24.434256
15.O	14.580016	18.053578	24.535617
16.H	16.111932	19.221477	23.859324
17.O	15.866582	21.957281	25.862186
18.H	19.382517	20.611554	29.041462
19.O	18.197165	22.140267	27.633424
20.H	24.425593	20.404016	25.177840
21.O	19.045744	19.704815	28.905130
22.N	24.445490	20.257044	26.189445
23.O	16.989347	18.396500	26.147075
24.N	22.263063	16.932726	27.355535
25.H	25.303107	18.323103	27.982399
26.C	24.317403	18.175544	27.552592
27.H	25.395629	20.362349	26.537654
28.C	23.842479	19.069697	26.610202
29.H	13.794744	18.141006	25.105994
30.H	23.828201	16.330884	28.666267
31.N	22.567561	18.823392	26.035839
32.H	22.120110	19.552271	25.488578
33.C	21.746204	17.780106	26.426103
34.O	20.597459	17.645677	25.934647
35.C	23.538710	17.084431	27.950767
36.H	19.236502	17.360107	26.948229
37.H	18.764467	22.278469	26.850792
38.C	14.808778	22.182092	26.828716
39.H	15.213137	22.418231	27.820741
40.H	14.134173	21.320231	26.904274
41.H	14.247997	23.042188	26.456162

Attachment Position 6

glc-G

glc-G, Bond Energy -5922.41 kcal/mol

1.N	-8.867112	2.657326	-4.193017
2.C	-9.536005	3.645262	-3.552334
3.N	-10.875661	3.937259	-3.651750
4.H	-11.553916	3.450103	-4.227035
5.C	-9.026204	4.588361	-2.641159
6.N	-10.041458	5.436913	-2.194504
7.C	-7.639626	4.546408	-2.328184
8.H	-12.117076	5.438013	-2.729308
9.N	-6.975544	3.528785	-3.034015
10.H	-5.952888	3.490913	-2.928422
11.C	-11.127017	5.015999	-2.822261
12.O	-6.996278	5.283394	-1.536039
13.C	-7.568599	2.633274	-3.898897
14.N	-6.765163	1.670255	-4.420874
15.H	-5.758732	1.760849	-4.347857
16.H	-7.124685	1.133832	-5.200683
17.C	-1.422508	0.046490	-1.088966
18.O	-0.450385	-0.494364	-0.184609
19.H	-2.253698	-0.666157	-1.103985
20.O	-4.137616	3.326427	-2.926531
21.O	-1.937547	1.232551	1.708345
22.H	-0.998184	0.118387	-2.102133
23.O	-4.231144	2.898929	1.902505
24.H	-4.628585	3.787672	1.972753
25.O	-4.630700	4.666562	-0.344118
26.H	-5.482303	4.832567	-0.834163
27.O	-2.831923	1.782309	-1.836019
28.H	-5.091528	2.639762	-0.624174
29.C	-3.410087	2.902271	0.712519
30.H	-2.592114	3.630570	0.836215
31.H	-3.597836	0.757869	0.521430
32.C	-1.965245	1.440486	-0.719782
33.H	-1.142084	2.167753	-0.658242
34.H	-3.578615	3.617490	-3.669290
35.C	-2.792945	1.507699	0.575719
36.C	-3.339843	3.130494	-1.774556
37.H	-2.501235	3.841507	-1.760029
38.C	-4.220125	3.304291	-0.524678
39.C	0.799090	0.232149	-0.168685
40.H	-2.453214	1.462971	2.503982
41.H	1.518993	-0.396042	0.361858
42.H	0.698636	1.190558	0.357718
43.H	1.155912	0.413699	-1.193117

glc-T

glc-T, Bond Energy -5619.38 kcal/mol

1.H	15.950327	18.158866	26.190895
2.H	25.372738	16.693728	26.541362
3.C	24.446967	18.520393	27.193393
4.C	18.117278	18.389594	26.059588
5.H	18.846618	18.585228	25.258526
6.C	18.179697	19.522058	27.091435
7.H	17.465881	19.297442	27.899354
8.C	17.801947	20.868342	26.471551
9.H	18.551070	21.145327	25.717800
10.C	16.416234	20.759352	25.798093
11.H	15.664307	20.545650	26.573136
12.C	16.060737	22.037882	25.038375
13.H	16.142343	22.892189	25.726645
14.O	14.758564	22.019702	24.428082
15.H	16.776044	22.173441	24.219404
16.O	17.806422	21.834406	27.552732
17.H	19.567124	20.389682	28.181562
18.O	19.512967	19.572217	27.649731
19.N	23.299296	16.565104	26.410687
20.O	18.372161	17.116518	26.667170
21.H	17.318572	17.465166	23.816000
22.O	16.441210	19.673255	24.835601
23.O	16.557103	17.407074	24.425272
24.C	16.709332	18.380067	25.427830
25.C	23.154786	19.164553	27.389900
26.O	22.988542	20.319365	27.825532
27.N	22.033684	18.387660	27.051707
28.H	21.109552	18.823192	27.200747
29.C	22.043738	17.101520	26.570877
30.C	25.701331	19.281775	27.531946
31.H	25.770569	20.202562	26.939940
32.H	26.588900	18.673036	27.337179
33.H	25.703977	19.578293	28.588055
34.C	24.457530	17.247830	26.713535
35.H	23.347565	15.612964	26.062905
36.O	21.006500	16.460694	26.295859
37.H	19.338853	16.916714	26.567089
38.H	18.113399	22.687635	27.200443
39.C	13.673750	22.157243	25.373757
40.H	13.819642	23.046592	26.003838
41.H	13.579939	21.271067	26.015836
42.H	12.760461	22.271205	24.784844

glc-A

glc-A, Bond Energy -5756.75 kcal/mol

1.C	3.104193	-4.792494	-2.065950
2.O	4.212697	-5.318583	-1.312465
3.H	2.246950	-5.432652	-1.835988
4.H	3.314071	-4.856254	-3.143071

5.O	2.230337	-3.798822	0.644086
6.H	0.128904	-0.616949	0.582934
7.O	0.432473	-1.543965	0.650728
8.H	-0.819401	0.031119	-4.520668
9.O	0.734591	0.342878	-1.446753
10.H	-0.090256	0.593157	-1.974023
11.O	2.240891	-2.717368	-2.925829
12.N	-2.541468	1.597330	-0.788915
13.C	-1.745297	0.293820	-4.017697
14.C	2.781580	-3.325103	-1.722515
15.H	3.713953	-2.807476	-1.443588
16.H	-5.678500	0.016478	-5.183735
17.N	-2.886174	0.083756	-4.688960
18.C	-3.970145	0.428758	-3.959096
19.N	-5.302397	0.352276	-4.303912
20.H	-3.341588	1.906666	-0.253410
21.H	-1.615379	1.782590	-0.425777
22.N	-5.264486	1.185743	-2.209564
23.O	1.501835	-0.816245	-4.008000
24.H	-7.109072	0.854488	-3.250835
25.C	2.012108	-1.290158	-2.784437
26.H	2.965931	-0.799839	-2.533797
27.C	0.968156	-1.051903	-1.683254
28.H	0.035610	-1.541463	-2.002373
29.C	1.444183	-1.688593	-0.369455
30.H	2.363562	-1.170386	-0.045595
31.N	-1.599049	0.788862	-2.768894
32.H	2.242693	-0.712563	-4.632874
33.C	-6.029786	0.814131	-3.227830
34.C	5.494907	-4.901624	-1.833887
35.C	-3.963180	0.949344	-2.652802
36.C	-2.700071	1.128295	-2.044262
37.C	1.763839	-3.171633	-0.569531
38.H	0.844128	-3.707079	-0.832915
39.H	6.256306	-5.357671	-1.196681
40.H	5.607771	-3.809236	-1.805166
41.H	5.621041	-5.248741	-2.869867
42.H	3.095601	-3.411159	0.876143

glc-C

glc-C, Bond Energy -5407.76 kcal/mol

1.H	21.685993	16.835536	27.492257
2.O	14.975997	17.524851	24.551965
3.C	15.475040	18.227467	25.665158
4.H	14.688119	18.192840	26.431030
5.C	16.793083	17.663908	26.207205
6.H	17.541957	17.722509	25.399724
7.C	17.271157	18.502772	27.406839
8.H	16.524664	18.415147	28.207856
9.C	17.391379	19.985037	27.025202
10.H	18.215227	20.111669	26.310381
11.C	16.066241	20.462857	26.376193
12.H	15.271155	20.425108	27.137527
13.C	16.181399	21.876420	25.831529

14.H	16.584708	22.534307	26.616197
15.O	14.882651	22.340479	25.424564
16.H	16.875876	21.887842	24.975136
17.O	17.665953	20.727885	28.236909
18.H	17.370597	16.064846	27.158893
19.O	18.485187	17.949959	27.945579
20.H	24.318344	21.485697	25.755666
21.O	16.598332	16.290450	26.602813
22.N	24.402378	21.146490	26.716332
23.O	15.708971	19.597216	25.264725
24.N	22.265298	17.654852	27.347300
25.H	25.351204	18.903239	28.041671
26.C	24.340142	18.839184	27.652390
27.H	25.374182	21.183304	27.015158
28.C	23.814836	19.898535	26.935081
29.H	15.611955	23.770739	24.067162
30.H	23.905614	16.808144	28.405882
31.N	22.509494	19.763340	26.391521
32.H	22.023026	20.597795	26.075650
33.C	21.693897	18.671182	26.646274
34.O	20.508317	18.642321	26.234255
35.C	23.578591	17.687520	27.873660
36.H	15.708602	17.378160	23.922592
37.H	19.227492	18.148693	27.311417
38.H	18.205808	21.503651	28.007619
39.C	14.931967	23.694198	24.927767
40.H	15.267765	24.386659	25.713838
41.H	13.917868	23.956205	24.617313

Table S6. Steady-State Kinetics for Single Nucleotide Insertions with KF^- Polymerase opposite **T**, **T***, **glc** and **6dglc** in the DNA template.

Template	dNTP	Km (μM)	Vmax (%)	Eff (Vmax/Km)	Fidelity
T	dATP	2.5 (4.0)	13.4 (15.4)	2.4 10^7 (2.1 x 10^7)	1
T	dTTP[8]	180 (20)	0.079 (0,002)	4.4 x 10^2	0.00002
T*	dATP	5.7 (3.5)	0.8 (0.4)	2.1 10^5 (1.5 x 10^5)	1
T*	dTTP	8.5 (3.7)	0.018 (0.004)	2.4 10^3 (1.2 x 10^3)	0.011
glc	dATP	1.3 (0,5)	0.012 (0,005)	8.7 10^3 (3.3 x 10^3)	1
glc	dTTP	91.3 (22.0)	0.0013 (0.0004)	13.8 (1.4)	0.0016
glc	dCTP	587.1	0.0019	3.30	0.0004
glc	dGTP	7.8 (4.7)	0,011 (0.006)	1.4 10^3 (0.5 x 10^3)	0.16
6dglc	dATP	1.2 (0,5)	0.013 (0,009)	1.2 10^4 (5.2 x 10^3)	1
6dglc	dTTP	70.1 (40,7)	0.0037 (0,0006)	67.8 (44.2)	0.006
6dglc	dCTP	512.1 (318.8)	0.0035 (0.0005)	8.2 (3.5)	0.0007
6dglc	dGTP	244.7 (241.2)	0,009 (0.008)	36.7 (11.7)	0.003

References

[1] E. Vengut-Climent, I. Gomez-Pinto, R. Lucas, P. Penalver, A. Avino, C. Fonseca Guerra, F. M. Bickelhaupt, R. Eritja, C. Gonzalez, J. C. Morales, *Angew Chem Int Ed Engl* **2016**, *55*, 8643-8647.

[2] L. J. Liu, J. H. Hong, *Bull Korean Chem Soc* **2011**, *32*, 411-416.

[3] M. K. Schlegel, E. Meggers, *J Org Chem* **2009**, *74*, 4615-4618.

[4] a) G. te Velde, F. M. Bickelhaupt, E. J. Baerends, C. Fonseca Guerra, S. J. A. van Gisbergen, J. G. Snijders, T. Ziegler, *J. Comput. Chem.* **2001**, *22*, 931-967; b) C. Fonseca Guerra, J. G. Snijders, G. te Velde, E. J. Baerends, *Theor. Chem. Acc.* **1998**, *99*, 391-403; cA. Version, *Theoretical Chemistry*, SCM, Vrije Universiteit: Amsterdam, The Netherlands,.

[5] C. Fonseca Guerra, T. van der Wijst, J. Poater, M. Swart, F. M. Bickelhaupt, *Theor. Chem. Acc.* **2010**, *125*, 245-252.

[6] aA. Klamt, G. Schuurmann, *J. Chem. Soc., Perkin Trans. 2* **1993**, 799-805; bA. Klamt, *J Phys Chem* **1995**, *99*, 2224-2235; cC. C. Pye, T. Ziegler, *Theor. Chem. Acc.* **1999**, *101*, 396-408.

[7] D. K. Fygenson, M. F. Goodman, *J Biol Chem* **1997**, *272*, 27931-27935.

[8] S. Moran, R. X. Ren, E. T. Kool, *Proc Natl Acad Sci U S A* **1997**, *94*, 10506-10511.
